# The genomic architecture of EBV and infected gastric tissue from precursor lesions to carcinoma

**DOI:** 10.1186/s13073-021-00963-2

**Published:** 2021-09-07

**Authors:** Zhang-Hua Chen, Shu-Mei Yan, Xi-Xi Chen, Qi Zhang, Shang-Xin Liu, Yang Liu, Yi-Ling Luo, Chao Zhang, Miao Xu, Yi-Fan Zhao, Li-Yun Huang, Bin-Liu Liu, Tian-Liang Xia, Da-Zhi Xu, Yao Liang, Yong-Ming Chen, Wei Wang, Shu-Qiang Yuan, Hui-Zhong Zhang, Jing-Ping Yun, Wei-Wei Zhai, Mu-Sheng Zeng, Fan Bai, Qian Zhong

**Affiliations:** 1 Biomedical Pioneering Innovation Center (BIOPIC), Integrated Research Building Room 330, School of Life Sciences, Peking University, Yiheyuan Road No.5, Haidian District, Beijing, 100871 China; 2 State Key Laboratory of Oncology in South China, Collaborative Innovation Center for Cancer Medicine, Sun Yat-sen University Cancer Center, Guangzhou, 510060 China; 3 Department of Pathology, Sun Yat-sen University Cancer Centre, Guangzhou, China; 4 Department of Ultrasound, The Fifth Affiliated Hospital of Sun Yat-sen University, Zhuhai, China; 5 Department of Oncology, Second Affiliated Hospital, Guangzhou Medical University, Guangzhou, China; 6 Institute for Computational Biomedicine, Weill Cornell Medicine, New York, USA; 7 Division of Hematology/Oncology, Department of Medicine, Weill Cornell Medicine, New York, USA; 8 Department of Gastric Surgery, Sun Yat-sen University Cancer Center, Guangzhou, China; 9 Key Laboratory of Zoological Systematics and Evolution, Institute of Zoology, Chinese Academy of Sciences, Beijing, China; 10 Center for Excellence in Animal Evolution and Genetics, Chinese Academy of Sciences, Kunming, China; 11 Beijing Advanced Innovation Center for Genomics (ICG), Peking University, Beijing, China

## Abstract

**Background:**

Epstein-Barr virus (EBV)-associated gastric carcinomas (EBVaGCs) present unique molecular signatures, but the tumorigenesis of EBVaGCs and the role EBV plays during this process remain poorly understood.

**Methods:**

We applied whole-exome sequencing, EBV genome sequencing, and whole-genome bisulfite sequencing to multiple samples (*n* = 123) derived from the same patients (*n* = 25), which covered saliva samples and different histological stages from morphologically normal epithelial tissues to dysplasia and EBVaGCs. We compared the genomic landscape between EBVaGCs and their precursor lesions and traced the clonal evolution for each patient. We also analyzed genome sequences of EBV from samples of different histological types. Finally, the key molecular events promoting the tumor evolution were demonstrated by MTT, IC50, and colony formation assay *in vitro* experiments and *in vivo* xenograft experiments.

**Results:**

Our analysis revealed increasing mutational burden and EBV load from normal tissues and low-grade dysplasia (LD) to high-grade dysplasia (HD) and EBVaGCs, and oncogenic amplifications occurred late in EBVaGCs. Interestingly, within each patient, EBVaGCs and HDs were monoclonal and harbored single-strain-originated EBV, but saliva or normal tissues/LDs had different EBV strains from that in EBVaGCs. Compared with precursor lesions, tumor cells showed incremental methylation in promotor regions, whereas EBV presented consistent hypermethylation. Dominant alterations targeting the PI3K-Akt and Wnt pathways were found in EBV-infected cells. The combinational inhibition of these two pathways in EBV-positive tumor cells confirmed their synergistic function.

**Conclusions:**

We portrayed the (epi) genomic evolution process of EBVaGCs, revealed the extensive genomic diversity of EBV between tumors and normal tissue sites, and demonstrated the synergistic activation of the PI3K and Wnt pathways in EBVaGCs, offering a new potential treatment strategy for this disease.

**Supplementary Information:**

The online version contains supplementary material available at 10.1186/s13073-021-00963-2.

## Background

Gastric cancer is the 3rd most lethal cancer worldwide and has a 5-year survival rate lower than 40% [1]. A notably high incidence of gastric cancer is observed in eastern Asia. Of all gastric carcinomas (GCs), approximately 10%, are associated with Epstein-Barr virus (EBV) [2]. EBV is linked with a variety of malignancies, including some lymphomas, undifferentiated nasopharyngeal carcinomas (NPCs), and EBV-associated GCs (EBVaGCs). Among these EBV-linked malignancies, EBVaGCs are most prevalent and account for more than 50,000 new cases each year worldwide [3].

Recently, several large-scale studies have revealed the distinct genomic profile of EBVaGCs in comparison with that of EBV-negative GCs. EBVaGCs frequently possess somatic mutations in *PIK3CA*, loss of *CDKN2A* expression, extreme DNA hypermethylation, and focal amplification of 9p24.1 (encompassing *JAK2*, *PD*-*L1*, and *PD*-*L2*), which suggest that EBV has an important function in tumorigenesis of EBVaGCs [4–6]. It has been proposed that the emergence of premalignant genetic changes is coupled with the aberrant establishment of EBV infection [7]. However, little is known about the molecular sequential order of EBVaGC tumorigenesis and the genetic alterations mediating the development of EBVaGC.

A systematic study of the genomic heterogeneity and temporal evolution of cancer cells and EBV is pivotal to illuminating the oncogenic processes of EBVaGCs and the role of EBV in these processes. Furthermore, an integrated investigation of the genomic and epigenomic evolutionary trajectories of EBVaGCs and EBV is of clinical importance in molecular-based diagnosis and personalized treatment. In this study, we applied whole-exome sequencing, EBV genome sequencing, and whole-genome bisulfite sequencing to 123 samples, including saliva (*n* = 10), normal epithelial tissues (*n* = 17), dysplasia (*n* = 28), and EBVaGCs (*n* = 68), from 25 patients. Through integrative analysis, we portrayed the (epi) genomic evolution process of EBVaGCs and revealed the extensive genomic diversity of EBV between tumors and normal tissue sites. We also validated the key driver events during the tumor evolution process by MTT, IC50, and colony formation assay *in vitro* experiments and *in vivo* xenograft experiments.

## Methods

### Patient enrollment

Collection and publication of human genetic data in this study were approved by the Ministry of Science and Technology of China. This study was approved by the institutional review board of the Sun Yat-Sen University Cancer Center. All samples collected in this study were obtained with informed patient consent. RNA in situ hybridization (RISH) analysis was performed on primary gastric tumors using Epstein-Barr-encoded RNA (*EBER*) probe to identify Epstein-Barr virus (EBV)-positive tumors. Formalin-fixed paraffin-embedded (FFPE) tissue blocks from patients who underwent resection of the primary gastric tumor during the period from December 2013 to December 2016 and received no previous treatment at Sun Yat-Sen University Cancer Center were retrieved from the Tumor Resource Bank of Sun Yat-Sen University Cancer Center and subjected to *EBER* RISH examination. Consequently, FFPE blocks from 20 patients were identified with the presence of both EBV-associated gastric carcinomas (EBVaGCs) and precursor lesions. Clinical information, including tumor stage, histological features (Lauren classification), and immunohistochemical results, are summarized in Additional file 1: Table S1.

### Sample collection

EBV-positive tumors were identified by RNA in situ hybridization (RISH) analysis using *EBER* probe. Formalin-fixed paraffin-embedded (FFPE) tissue blocks from 20 patients with the presence of both EBVaGCs and precursor lesions were obtained. Through independent pathological review by 3 pathologists, the consensus areas of EBVaGC, high-grade dysplasia (HD), low-grade dysplasia (LD), and morphologically normal epithelial tissue (N) neighboring tumors or dysplasia were identified. Moderately graded dysplasia samples were categorized as LD in this work due to their limited number. In 20 patients, normal epithelial tissues and dysplasia samples neighboring tumor nests and 2–5 spatially separated regions (at least ≥ 0.5 cm away from each other) within each independent tumor nest from each individual were marked. Consequently, a total of 109 distinct marked areas of interest were isolated via laser capture microdissection (LCM) using a Leica LMD7000 Microsystem (Wetzlar). Meanwhile, matched control samples were collected, including 2 distant normal gastric tissues (≥ 5 cm from tumor site; P5 and P13) and 18 blood samples. To investigate the diversity of EBV genomes within each individual, saliva samples (2 mL) from 5 of these 20 patients were also included in this study. Paired EBVaGCs and saliva samples from 5 additional patients without precursor lesions were added to the EBV genome analyses (Additional file 1: Table S1). The number and histological type of all collected samples are listed in Additional file 1: Table S2.

### Library construction and sequencing

#### Whole-exome sequencing

For 107 samples and 20 matched control samples, 50–200 ng DNA was sheared into 300–500-bp fragments using the Covaris ultrasonic system (Covaris). The fragmented DNA was end-repaired, 5′-phosphorylated, and ligated to sequencing adaptors using the Sure Select library preparation kit (Agilent Technologies) following the manufacturer’s instructions. The whole exonic regions of each sample were captured using the Sure Select V6 whole exon kit (Agilent Technologies).

#### EBV genome sequencing

Seven normal tissues, 7 LDs, 5 HDs, 65 EBVaGCs, and 10 saliva samples with a minimum amount of 50 ng DNA were subjected to whole EBV genome capture. DNA was sheared into 300–500-bp fragments, followed by end-repair, 5′-phosphorylation, and adaptor ligation using the VAHTS Universal DNA Library Prep Kit (Vazyme). The EBV Enrichment Assay Kit2 (MyGenostics) was used to capture the EBV genomic regions. To reduce the level of sequence contamination from humans and other organisms, hybridization with the EBV genome bait of each HD and EBVaGC sample was processed in two rounds before the final enrichment PCR.

#### Whole-genome bisulfite sequencing

Three normal epithelial tissues, 3 LDs, and 6 EBVaGCs with available DNA (at least 100 ng) from 3 patients (P7, P11, and P13) were subjected to library construction. To validate the efficiency of the bisulfite conversion, unmethylated λ DNA (Promega) was spiked into the samples. The protocol for library construction was a modified version of the post-bisulfite adaptor tagging (PBAT) method [8]. In brief, genomic DNA was first treated with bisulfite using the EZ DNA Methylation Kit (Zymo Research). Next, the first strand was synthesized using high-concentration Klenow (exo-) (Qiagen). The untagged first strand primer was digested using Exonuclease I (New England Biolabs), after which the first strand product was purified and subjected to second-strand synthesis and adaptor tagging. Finally, the product was amplified for subsequent sequencing.

All library products were quality-checked and paired-end sequenced on the Illumina Xten platform. For the exome sequence data, the average sequencing depths of 102.67-fold and 87.21-fold were achieved for the tumor and control samples, respectively. The sequencing information for all samples is listed in Additional file 1: Tables S3-S5.

### Mutation calling

For the exome sequence data, paired-end 150 bp fastq files were aligned to the reference genome hg19 build (UCSC) using BWA (BWA-0.5.9) with default parameters to generate a binary sequence alignment map (BAM) file [9]. The aligned BAM files were sorted and merged using Samtools 0.1.19 [10]. Duplications of sequencing reads were marked and excluded using Picard tools. All insertions and deletions (INDELs) were realigned, after which the base quality was recalibrated using the Genome Analysis Toolkit (GATK2.1-8) [11]. Next, the BAM file of each sample was independently subjected to somatic mutation calling. We first applied MuTect to identify single nucleotide variations (SNVs) and indel locator to identify short INDELs [12]. Next, we used Strelka, another somatic mutation calling tool, to detect SNVs and INDELs [13]. Mutations identified by both methods were reserved and annotated by SNPEFF. To ensure the fidelity of all called mutations, we set the following criteria as an additional filter to select reliable somatic mutations: (1) mutations listed in the National Heart, Lung, and Blood Institute Exome Sequencing Project were removed and (2) contamination by germline SNPs was minimized by removing mutations deposited in dbSNP 135 unless they were documented in the Catalog of Somatic Mutations in Cancer (COSMIC).

Based on the independent identification of somatic mutations in each sample, we adopted a force calling strategy to improve the sensitivity of mutation calling from multiple samples within the same patient [14]. In brief, for each mutation identified in at least one sample as stated above, we counted the number of reads (read quality ≥ 5) supporting the alternate or reference base (base quality ≥ 20) in other matched samples within the same patient. A mutation was selected if more than 3 reads covered the mutational site and the variant allele frequency (VAF) was higher than 0.02. After mutation calling, we evaluated the extent of mutational heterogeneity based on the heterogeneity index (HI) between each sample pair within the same patient as described in our previous study [15].

### Determination of putative driver mutations

To identify putative driver mutations in EBVaGCs and their precursor lesions, we first selected genes on the basis of recent large cohort studies of gastrointestinal malignancy, the COSMIC Cancer Gene Census, and the Kyoto Encyclopedia of Genes and Genomes (KEGG) pathways in cancer. Next, the likely driver status of all non-silent mutations in these genes was manually evaluated based on the following three aspects: (1) the exact mutations or mutational sites were in recurrent mutation hotspots, (2) the exact mutations or mutational sites were documented in the COSMIC database (gastric cancer-associated or related to other cancer types), and (3) the mutations were predicted to be deleterious (including nonsense, frameshift, and splicing mutations). Mutations were classified as putative driver mutations if they matched one of the requirements listed above. Furthermore, we involved 26 EBVaGCs samples from the TCGA cohort to corroborate the occurrence of driver events during the evolutionary process from precursor lesions to EBVaGCs [5].

### Mutational signature analysis

Single nucleotide variations (SNVs) could be categorized into 6 directions, namely, C > T, C > A, C > G, T > C, T > G, and T > A. Considering the 5′ and 3′ flanking nucleotides of a specific mutated base, a total of 96 substitution types exist. We first plotted the “lego” plots to compare the frequency of mutations within specific contexts in precursor lesions and EBVaGCs of 20 patients. As the set of mutational contexts of tumor samples was an imprint of the mutational process that shaped the cancer genome, we then performed a mutational signature analysis of all silent and non-silent mutations in our study. To extract the underlying mutational signatures in single precursor lesion samples and EBVaGCs, we applied the R package deconstructSigs [16] to each sample using the 30 signatures documented by the COSMIC as reference. After extraction, we calculated and compared the mean weights of different signatures in 45 precursor lesions and 65 EBVaGCs.

### Cancer cell fraction clustering

For each somatic mutation, the cancer cell fraction (CCF) was calculated by ABSOLUTE by integrating the purity of the sample, local copy number, and allele counts. In addition, we conducted an analysis to cluster the mutations using the PyClone method [17]. In principle, the assumption that each sample had a number of distinct populations carrying different mutations allowed us to infer (1) the number of clones that co-existed within the sample, (2) the clonality of different clones, and (3) the number of mutations within each clone. For each mutation, we used the binomial distribution to model the observed mutant read counts. The fraction of cells carrying a particular mutation was determined with a Dirichlet process with a base distribution of *U* (0, 1). The Markov chain Monte Carlo (MCMC) sampling method was adopted to obtain the posterior distribution of the parameters. The MCMC chain was run for 5000 iterations, and the initial 500 iterations were treated as a burn-in period and discarded. In each sample, a particular mutation was considered as subclonal if the defined clone comprising this mutation had a mean CCF value less than 0.8.

### Construction of phylogenetic trees

For each patient, a phylogenetic tree was constructed using both mutations and CNAs. First, consistent with the method used in our previous study [15], we extracted a sequence encompassing the mutations (total length of 21 bp) to infer the phylogeny among samples from each patient based on the maximum likelihood algorithm. The original phylogenetic tree explained the presence and absence of most mutations among samples in each patient, which also directly reflected the extent of relatedness among samples. In this step, there existed a small proportion of mutations that did not fit the phylogenetic tree of each patient, perhaps because of the absence of mutations due to copy number loss, a low level of clone mixing, or technical noise.

The assignment of CNAs to phylogenetic trees was conducted by checking the breakpoints (start and end) and allelic copy numbers of altered segments. Considering that breakpoint identification was affected by the exome capture of different samples in each patient, we set a tolerance value of 500 bp when we judged whether one CNA event harbored the same breakpoints in multiple samples. After the assignment of CNAs, the length of each line on the phylogenetic trees was adjusted to be proportional to the number of mutations and CNAs.

### EBV genome alignment, SNV calling, and construction of phylogenetic trees

For each EBV genome sequence, paired-end data were first realigned to a reference panel composed of 67 complete EBV genomes downloaded from the NCBI using BWA. These 67 references covered EBV genomes from different geographical origins and cancer types (including one saliva sample from a healthy individual) (Additional file 1: Table S6) [18]. Furthermore, the potential capture bias resulting from the differences between type 1 and type 2 EBV was not observed in our results. Based on the realigned EBV sequences, we ranked all reference genomes by counting the number of uniquely mapped paired-end reads with high quality (mapping quality ≥ 20 and length ≥ 50 bp) and selected the top-ranked EBV reference genome as the most probable origin of the EBV genome in each sample. Next, for each sample, we realigned the EBV sequence data to the specific reference genome using BWA. The average proportion of paired reads that mapped to the reference genome was 82.68% (Additional file 1: Table S4). The realigned BAM files were marked and sorted, after which duplicates were removed.

The processed BAM files of the EBV sequences were used as input for SNV identification on the EBV genome in each sample. In detail, we used Samtools to obtain a list of potential SNVs. To discover reliable SNVs, we set a series of strict filters and statistical tests to reduce the frequency of false positives, like sequencing errors and misalignments. Using custom Perl scripts, SNVs that did not meet all of the thresholds (Additional file 1: Table S7) were removed from the mutational list. After identification of SNVs, we constructed phylogenetic trees of EBV genomes using genes with variable sequences. We used MAFFT for multiple-sequence alignment [19] of all 94 samples and 67 reference genomes (samples and references were excluded if the corresponding GenBank files lacked annotation for the genes used to construct the phylogenetic trees). Next, we utilized the maximum likelihood method implemented in RAxML based on the GTR+G+I model to generate the phylogenetic tree of each gene [20]. Gene-level selection analysis was performed using the BUSTED package on the Datamonkey webserver [21, 22].

### DNA methylation analysis

For whole-genome bisulfite sequencing data, 150-bp paired-end reads were first trimmed of adaptors and 6-bp random primers. Next, we assessed the sequencing quality using Trim_galore (Trim Galore 0.4.4) in single-end mode. The trimmed reads were aligned to a combined reference consisting of the hg19 human genome and the complete wild-type EBV reference genome (accession number NC_007605). Alignment and calculation of methylation levels (*β* values) were conducted using Bismark v0.19.0 in single-end, non-directional mode [23]. We also aligned the reads to the λ DNA for conversion efficiency assessment. For the human genome, by calculating the Euclidean distance of *β* values between any two samples from the same patient, we identified significant differences in the global methylation levels of normal tissues/LDs and EBVaGCs. To further characterize the epigenetic changes during tumor development, DNA methylation profiles were processed with smoothing, after which differentially methylated regions (DMRs) were identified using the R package bsseq [24]. The criteria used to identify significant DMRs were as follows: a detection *P* value less than 0.05 and a difference of at least 0.3 in the mean *β* value of the region between 6 normal tissues/LDs and 6 EBVaGCs. The genomic context of all qualified DMRs was determined using the R package genomation [25]. Analysis of the functional enrichment of genes with promoter regions (transcription start site (TSS) ± 2000 bp) that overlapped with DMRs was performed using Metascape.

For the EBV genome, due to the low coverage (Additional file 1: Table S5) of normal tissues and LDs, we merged the sequencing reads of EP13-N1 and EP13-LD. To investigate the epigenetic changes during tumor development, we plotted the *β* values along the EBV genomes of EP13-C1, EP13-C5, and EP13-N1/LD, which were annotated according to the NC_007605 GenBank file. The two main repeat regions, IR1 (positions 12001–35355) and TR (positions 169636–171773), were not considered in the analysis due to sparse coverage.

### Virus quantity measurement

To compare the absolute number of EBV genome copies in blood, saliva, and different histological samples, we randomly selected samples from each group and performed real-time qPCR using the AceQ U+ Probe Master Mix Kit (Vazyme) on a Bio-Rad CFX96 system (Bio-Rad). The number of EBV genome copies was measured using a region located within the conserved gene *BamHI*. The forward primer (5′-CCCAACACTCCACCACACC-3′), reverse primer (5′-TCTTAGGAGCTGTCCGAGGG-3′), and probe (5′-FAM-CACACACTACACACACCCACCCGTCTC-BHQ1-3′) were designed based on the complete wild-type EBV reference genome (accession number NC_007605). The B95-8 plasmid was used to generate the standard curve. By comparing the threshold cycle numbers of each sample with the standard curve, the absolute number of EBV genome copies per 10 ng extracted DNA was determined. The experiments used to generate the standard curve were repeated three times at each magnitude.

### Giemsa staining

The tissue slides were deparaffinized and rehydrated. Naturally dried slides were incubated with Giemsa staining solution (Solarbio, Beijing, China, G1015) (diluted 10 times with distilled water) for 10–30 min, then rinsed with water. After that, the sections were incubated with Phenol red solution for 10–30 min, then rinsed with water. Then, the slides were covered with coverslips for further microscopic examination.

### Cell lines

All human GC cell lines, including AGS, SNU719, HGC27, SNU16, and SNU5, were kindly provided by Professor Ruihua Xu (Sun Yat-sen University Cancer Center). AGS-EBV cells were derived from AGS cells with stable EBV infection. All cell lines are grown in DMEM (Gibco-BRL Life Technologies, Carlsbad, USA) supplemented with 10% fetal bovine serum (FBS; Gibco-BRL Life Technologies). All cell lines were maintained in a humidified incubator at 37 °C with a 5% CO_2_.

### MTT assay

Cells were treated with PI3K inhibitor LY294002 (Selleck, S1105) or copanlisib (Selleck, S2802), Wnt pathway inhibitor ICG001 (Selleck, S2662) or mebendazole (Selleck, S4610), or a combination of two agents for the indicated period of time. LY294002 is an inhibitor of PI3Kα/δ/β, whereas copanlisib is an inhibitor of PI3Kα and PI3Kδ. ICG001 is an inhibitor of β-catenin, whereas mebendazole is an inhibitor of Traf2- and Nck-interacting kinase (TNIK), which interacts with TCF4 to regulate Wnt downstream targets, and 5-Aza-2′-deoxycytidine (5-aza) is the DNA methylation inhibitor. To evaluate the effect of PI3K inhibitors or Wnt pathway inhibitors, cells were seeded in 96-well plates at a density of 1500 cells per well. After an overnight incubation, cells were treated under the indicated conditions. To evaluate the effect of 5-aza, seeded cells were treated with 5-aza at a final concentration of 2.5 and 5 μM, respectively, with Aza-containing medium being changed every 24 h. At the end of the treatment, 20 μL of 3-(4,5-dimethylthiazol-2-yl)-2,5-diphenyltetrazolium bromide was added to each well, after which the plates were incubated at 37 °C for 4 h. Next, the supernatants were aspirated carefully, after which formazan crystals were dissolved in DMSO. Finally, the absorbance was measured at 490 nm. The concentration of each PI3K inhibitor, Wnt pathway inhibitor, and combination that produced 50% growth inhibition (IC50) was calculated using a relative survival curve 48 h after treatment. A drug combination experiment was performed by setting serial concentrations of one drug as above with a fixed concentration (IC25 or IC50) of another drug. Furthermore, the value of combination index (CI) calculated by the CalcuSyn software was used to determine the synergism between drug combinations [26–28].

### Colony formation assay

Cells (2000 cells per well) were seeded in a 6-well plate. After an overnight incubation, cells were treated under the indicated conditions or treatments and cultured for 14 days in a humidified 5% CO_2_ incubator at 37 °C. To evaluate the effect of 5-aza, seeded cells were treated with 5-aza at a final concentration of 2.5 and 5 μM, respectively, with Aza-containing medium being changed every 24 h. Colonies were fixed in methanol for 10 min and stained with 0.5% crystal violet for 15 min. All visible colonies were quantified. At least three independent experiments were performed for each assay.

### RNA extraction and semi-quantitative RT-PCR

EBV-positive gastric cancer cell lines, SNU719 and AGS-EBV, were seeded at a 6-well plate with a density of 1 × e05 cells/ml and incubated overnight. The medium was then replaced with a fresh medium containing 5-Aza at a final concentration of 2.5, 5, and 10 μM. 5-Aza treatment lasted for 72 h, with changing of Aza-containing medium every 24 h. For combined treatment of 5-aza with TSA, cells were treated with TSA (100 ng/ml in DMSO) for an additional 24 h after Aza treatment. Total RNA was extracted using TRI Reagent and then performed reverse transcription. cDNA products were amplified with Taq polymerase (Invitrogen) for 40 cycles, with ACTB (32 cycles) as the control. The sequences of primer sets are listed in Additional file 1: Supplementary Methods.

### Western blotting

Total protein was extracted from AGS-EBV cells treated with different treatments (copanlisib 0.05 μM, mebendazole 0.15 μM) or vehicle for 24 h using SDA lysis buffer. Protein lysate was separated via 9% SDS-PAGE. After electrophoresis, the proteins were transferred to the PVDF membrane (Millipore, Burlington, MA, USA). The membranes were subsequently blocked in 5% milk for 1 h. Then, the membrane was incubated with anti-phospho-Akt antibody (Try483) (GeneTex, GTX50128, China) and anti-GAPDH antibody (6004-1, Proteintech, Rosemont, IL, USA) separately at 4 °C overnight. The species-matched secondary antibodies were then hybridized with the membranes at room temperature for 45 min. The protein was visualized by enhanced chemiluminescence (Thermo, Waltham, MA, USA).

### Luciferase assay

AGS-EBV cells were seeded at 1 × 10^4^ cells per well in 48-well plates for 16 h and then transfected with TK-Renilla plasmid (10 ng) and TOPflash or FOPflash reporter plasmids (250 ng) using PEI (YEASEN, 40816ES03, China). After 4–6 h, fresh medium was changed, and cells were treated with LiCl (30 mM) and different treatments (vehicle, copanlisib 0.03 μM, mebendazole 0.15 μM, or the combination). The luciferase activities were detected using the Dual-Glo Luciferase Assay System (Promega, #E2940, Madison, WI, USA) at 24 h post-transfection. The relative luciferase activities were normalized against the values of the Renilla luciferase signal.

### *In vivo *assay

The study was performed on 4- to 5-week-old female immune-deficient mice (BALB/c nude mice) which were purchased from Model Animal Research Center of Nanjing University according to ethics approval number L102012017003G. BALB/c nude mice were housed in isolated cages, under specific pathogen-free (SPF) conditions with a 12-h light/dark cycle and maintained at 22 °C ± 1 at the animal facility of Cancer Prevention Center, Sun Yat-sen University. Mice were inoculated subcutaneously with 1 mm^3^ SNU719 xenograft tumor fragments. When the tumor reached the predetermined size, mice were randomized to the control or experimental group. Copanlisib was dosed intraperitoneal injection at 6 mg/kg with a Q2D schedule. Mebendazole was dosed at 20 mg/kg orally with a once daily schedule. Body weights and tumor volumes were measured every day. Tumors were removed and weighed under isoflourane anesthesia at the completion of the study. The following formula was used to determine tumor volumes: tumor volume = *L* × *W*^2^/2, in which *L* is the length and *W* the width.

### Quantification and statistical analysis

For the sequencing data, statistical analyses and graphics production were performed using R v3.5.0 (Foundation for Statistical Computing). For the experimental data, statistical analyses and graphics production were performed using GraphPad Prism 7 (GraphPad Software). All hypothesis tests were 2-sided. Statistical tests are specified in the “Results” section and the figure legends.

## Results

### The driver landscape of EBVaGCs and their precursor lesions

**Fig. 1:**
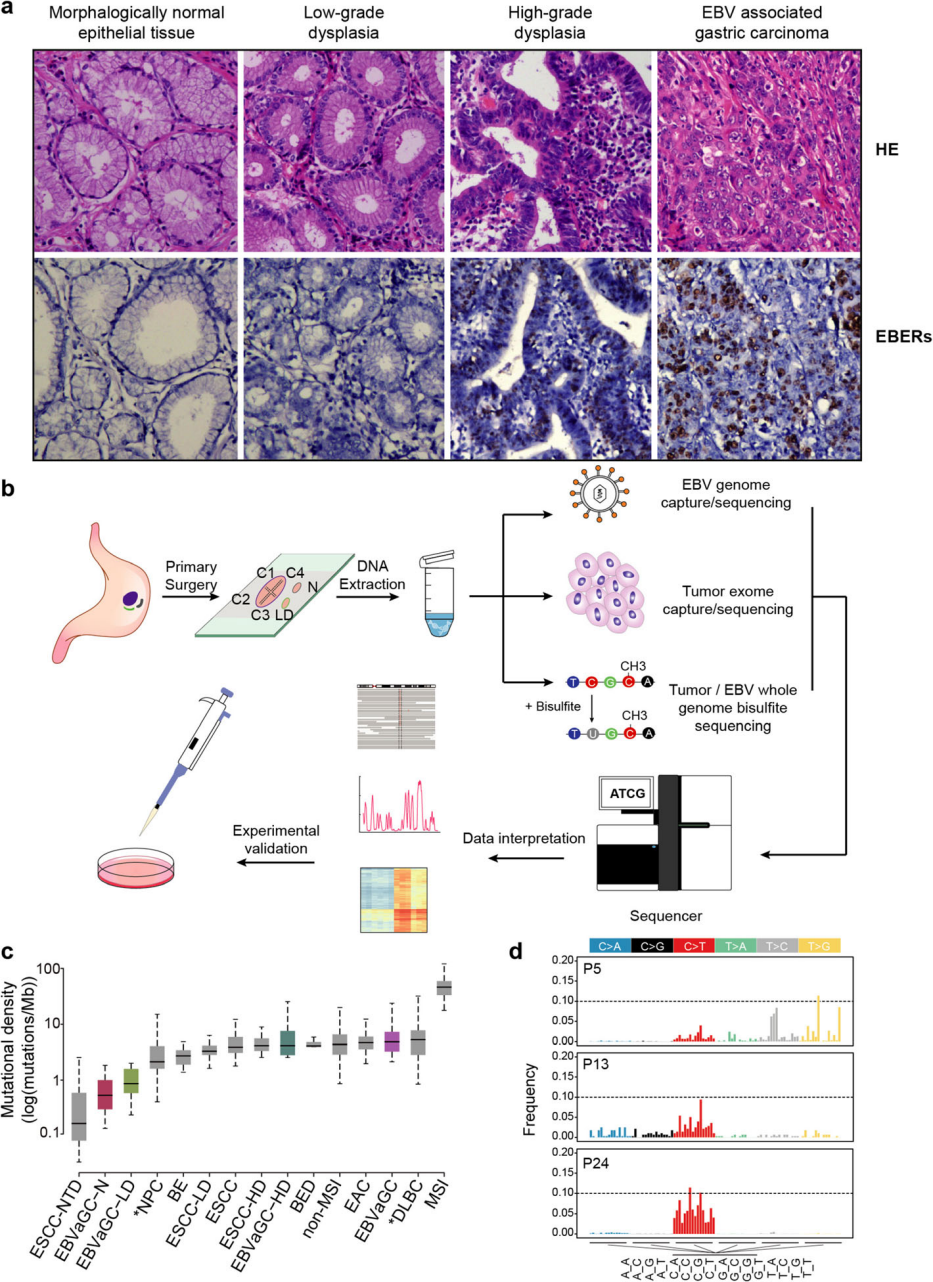
Mutational density and context of EBVaGCs and their precursor lesions. **a** Diagram showing the hematoxylin and eosin (HE)-stained (top) and in situ hybridization of Epstein-Barr-encoded RNA (bottom) sections of morphologically normal epithelial tissue (N), low-grade dysplasia (LD), high-grade dysplasia (HD), and EBV-associated gastric carcinoma (EBVaGC) from a representative patient P10. The magnification is 20-fold. **b** Schematic overview of the study design. Multiple regions covering different histological stages derived from the same patient were acquired. DNA was extracted and subjected to parallel genomic and epigenomic analyses for both host cells and EBV. **c** Comparison of the mutational density of normal tissues (*n* = 17), LDs (*n* = 19), HDs (*n* = 9), and EBVaGCs (*n* = 62) with other types of cancer. ESCC-NTD, esophageal squamous dysplasia from cancer-free patients (non-tumorous dysplasia, *n* = 13); NPC, nasopharyngeal carcinoma (*n* = 71); BE, non-dysplastic Barrett’s esophagus (*n* = 14); ESCC-LD, esophageal squamous low-grade dysplasia (*n* = 44); ESCC, esophageal squamous carcinoma (*n* = 62); ESCC-HD, esophageal squamous high-grade dysplasia (*n* = 31); BED, dysplastic Barrett’s esophagus (*n* = 11); non-MSI, non-microsatellite instable gastric carcinoma (*n* = 231); EAC, esophageal adenocarcinoma (*n* = 183); DLBC, diffuse large B cell lymphoma (*n* = 48); MSI, microsatellite instable gastric carcinoma (*n* = 64). Asterisk denotes other two EBV-positive cancer types. Box and whiskers denote the median, IQR, and 1.5 × IQR. The *y*-axis is shown on a log10-transformed scale. **d** Bar plots showing the mutational spectrum of three representative patients. Different colors denote the mutational direction, and the 3-bp mutational context are labeled below the *x*-axis

Primary EBVaGCs were identified by RNA in situ hybridization (RISH) using an Epstein-Barr-encoded RNA (*EBER*) probe (Fig. 1a, Additional file 1: Figure S1a). Twenty patients, who received surgery with no previous treatments during the period from December 2013 to December 2016 at Sun Yat-Sen University Cancer Center, with both EBVaGCs and precursor lesions after pathological review were included in our study (Additional file 1: Figure S1b). Simultaneous analysis of both precursor lesions and tumors derived from the same patient provides a unique opportunity to portray the process of tumor evolution under an identical germline background. In total, 107 samples comprising 62 EBVaGCs, 9 HDs, 19 LDs, and 17 normal tissues from 20 patients were subjected to whole-exome sequencing (Fig. 1b, Additional file 1: Tables S1-S2).

We found that the frequency of somatic mutation in HDs (median, 4.13 mutations/Mb) and EBVaGCs (median, 4.8 mutations/Mb) was significantly increased in comparison with that of normal tissues (median, 0.53 mutations/Mb) and LDs (median, 0.87 mutations/Mb) (Student’s *t* test, *P* = 8.45e−16) (Fig. 1c). The mutational density values of normal tissues and LDs were lower than that of the esophageal dysplasia samples (Student’s *t* test, *P* = 2.2e−16), but higher than that of esophageal squamous dysplasia from cancer-free patients in our previous study (Student’s *t* test, *P* = 0.003) [14, 15]. HDs and EBVaGCs had mutational densities comparable with those of two EBV-positive cancer types (nasopharyngeal carcinomas and diffuse large B cell lymphomas) and other esophageal/gastric tumors (Student’s *t* test, *P* = 0.09), with the exception of microsatellite instable (MSI) GCs (Student’s *t* test, *P* = 8.10e−13) [5, 29, 30].

With regard to mutational context, we observed a significant enrichment of C > T transitions at CpG dinucleotide positions in precursor lesions and EBVaGCs (Additional file 1: Figure S2a), which is associated with the spontaneous deamination of cytosine and accumulates along with aging [31]. We found a trend for increased T > G transversions at TpT dinucleotides private to EBVaGCs. The high proportion of T > G transversions corresponds with the COSMIC Signature 17 [4]. Consistently, we observed the heavier decomposed weight of this signature in EBVaGCs compared with that in precursor lesions (Additional file 1: Figure S2b); however, the origin of signature 17 is unknown, whether it is associated with EBV infection still needs verification. By decomposing mutational signatures, we could trace the potential mutagenic processes in each patient, as exemplified by the co-existence of multiple signatures in patient P13 (Additional file 1: Figure S2c-d). Overall, with the exception of signature 17, the predilection for these mutagenic processes was present in both EBVaGCs and precursor lesions from each patient, but interpatient heterogeneity was evident (Fig. 1d).

**Fig. 2:**
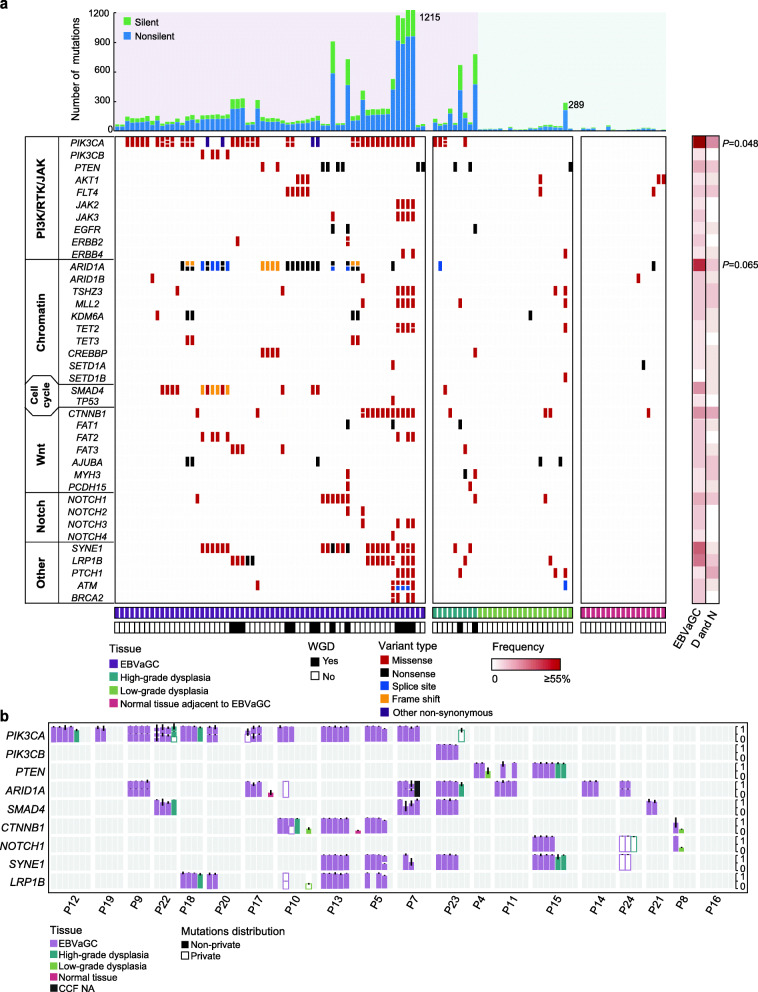
Mutational landscape of EBVaGCs, dysplasia samples, and normal tissues. **a** Top: the number of silent and non-silent mutations of each sample. Middle: somatic mutations (SNVs and INDELs) of EBVaGC-associated genes in different pathways. Genes are ranked by their mutated frequencies. Bottom: histological types and whole-genome doubling (WGD) status of each sample are indicated in different colors. Right: heatmap comparing mutated frequencies of each gene (row) in EBVaGCs and their precursor lesions over patients. The statistical significance is shown (Fisher’s exact test). **b** Bar plots showing the cancer cell fraction (CCF) of each mutation in recurrently mutated genes. Solid bars denote the mutations that are shared by multiple samples from the same patient. Hollow bars denote the mutations that are private in single samples. It should be noted that there existed multiple mutations in a specific gene within one sample, and these mutations are vertically stacked with an adjusted scale of CCF values. Different colors indicate different histological types. The standard deviation is indicated

In EBVaGCs, we identified frequently mutated genes, including *PIK3CA*, *PTEN*, *ARID1A*, *SMAD4*, *CTNNB1*, and *NOTCH1*. These genes were mainly clustered in the PI3K-Akt, chromatin-related, cell cycle regulation, Wnt, and Notch pathways (Fig. 2a). By comparing the frequency of mutated driver genes in EBVaGCs and their precursor lesions, we found that *PIK3CA* (*P* = 0.048) and *ARID1A* (*P* = 0.065) tended to be more prevalent in EBVaGCs. We also found the occurrence of driver mutations in precursor lesions (Fig. 2b). However, in dysplasia samples of EBVaGC, we observed very few mutations in *FBXW7* and *TP53*, but these two genes had high frequencies of mutations in gastric intestinal metaplasia as previously reported [32]. This finding reflects the distinct mutational profiles in the precancerous stage across different gastric cancer types.

### Copy number alterations promote the progression of EBVaGCs

**Fig. 3:**
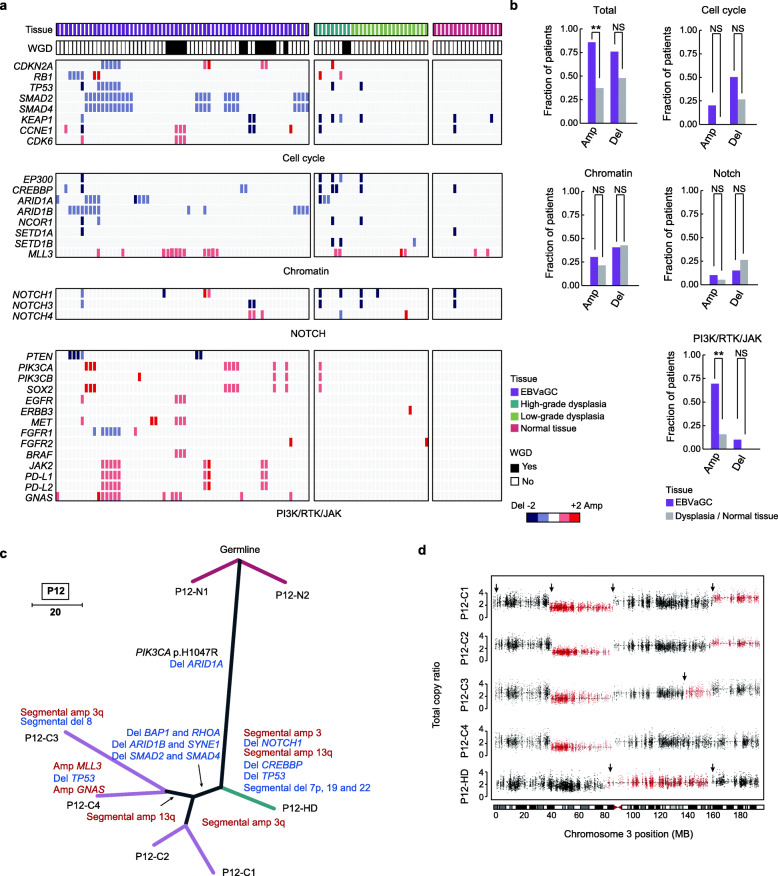
Copy number alterations of driver genes. **a** Diagram exhibiting the deletions (blue) and amplifications (red) of putative driver genes in EBVaGCs, dysplasia samples, and normal tissues. The histological types and whole-genome doubling (WGD) status of each sample are indicated on the top. **b** Bar plots comparing the frequencies of amplifications and deletions of different pathways in EBVaGCs and their precursor lesions over patients. Fisher’s exact test, **P < 0.01; NS, not significant. **c** Phylogenetic tree for patient P12. The length of each line is proportional to the number of mutations and copy number alterations (CNAs). Gray lines represent the clonal mutations shared by multiple samples. **d** Dot plots displaying the total copy ratio of segments in the HD sample (P12-HD) and 4 EBVaGCs from P12. Different segments are marked by red and black in turn, and breakpoints between two segments are indicated. The chromosome ideograms are shown on the bottom

Our analysis showed that the frequency of copy number alterations (CNAs) in precursor lesions was lower than that of EBVaGCs (Additional file 1: Figure S3a-b), whereas the CNAs of EBVaGCs were not extensive in comparison with those of GCs showing chromosome instability [5]. *TP53* aberrations were found to be rare in our cohort, but we identified higher frequencies of mutations in cell cycle–related genes in EBVaGCs compared with precursor lesions (Additional file 1: Figure S3c). The alterations in the cell cycle pathway and the aberrant activation of this pathway in tumor cells might explain the occurrence of the few WGD events in EBVaGCs (Additional file 1: Figure S3d) [15, 33, 34]. Examination of CNAs throughout the entire genome revealed that EBVaGCs harbored frequent amplifications in chr3q, chr7, chr9p, and chr20, as well as deletions in chr18q (all of these regions had a significance level of *q* < 0.05 in EBVaGCs) (Additional file 1: Figure S3b). We then evaluated the gene-level deletions and amplifications encompassing driver genes between EBVaGCs and their precursor lesions. Recurrent deletions in genes involved in cell cycle regulation, chromatin-related signaling, and Notch pathways, as well as amplifications in genes involved in the PI3K, RTK, and JAK pathways, were discovered (Fig. 3a). Interestingly, the frequency over patients of amplifications in EBVaGCs (85%) was significantly higher than that in precursor lesions (36.8%; *P* = 0.003), which was demonstrated by the greater prevalence of oncogenic amplifications in the PI3K/RTK/JAK pathways (70% versus 15.8%; *P* = 0.001) (Fig. 3b). In contrast, the frequencies of deletions and amplifications of genes in EBVaGCs and precursor lesions were similar in the chromatin-related (deletions, 40% versus 42.1%, *P* = 1.0; amplifications, 30% versus 21.1%, *P* = 0.716) and Notch (deletions, 15% versus 26.3%, *P* = 0.451; amplifications, 10% versus 5.3%, *P* = 1.0) pathways. For genes encoding cell cycle regulators, although we identified frequent deletions of *SMAD2*/*4* and amplifications of *CCNE1* and *CDK6* in EBVaGCs, the differences in the frequency over patients were not significant (deletions, 50% versus 26.3%, *P* = 0.191; amplifications, 20% versus 0%, *P* = 0.106).

Patient P12 exemplified a scenario in which amplifications occurred as late events. Although amplifications in chr3q were ubiquitously observed in 4 EBVaGCs and P12-HD, their breakpoints were non-identical (Fig. 3c, d). A similar result was also discovered for the amplifications in chr13q in 2 EBVaGCs (P12-C3 and P12-C4) and P12-HD. These findings indicated that the amplifications in chr3q and chr13q were acquired late as convergent events during tumor evolution in P12. Through analysis of homologous tracking, we did not observe the convergent evolution of loss-of-heterozygosity (LOH) events in our cohort (Additional file 1: Figure S4). Taken together, these findings indicated that deletions tended to occur early, but the amplifications of oncogenes were more likely to arise late during the development of EBVaGCs.

### EBVaGCs and their neighboring HDs had the same clonal origin

**Fig. 4:**
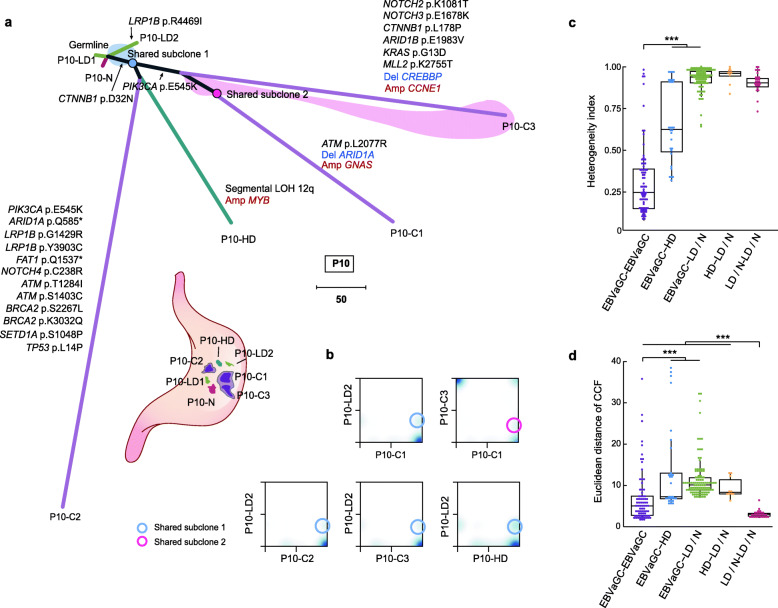
Phylogenetic relationships of multiples samples of representative patients. **a** Top: phylogenetic tree for patient P10. The length of each line is proportional to the number of mutations and copy number alterations (CNAs). Gray lines represent the clonal mutations shared by multiple samples. A subclone in one LD sample of P10 (P10-LD2) presenting as the common ancestor of all other histological advanced samples of P10, and another subclone shared by two EBVaGCs (P10-C1 and P10-C3) are indicated. The shaded area contains mutations present in the shared subclone on the corresponding branches. Bottom: geographical locations of all samples in patient P10. Histological types of all samples are indicated in different colors. **b** Two-dimensional density plots showing the CCF distribution of pairwise samples in P10. **c** Box plots depicting the heterogeneity index (HI) of pairwise samples in each patient. All pairwise samples are divided into 5 groups. Wilcoxon rank sum test, ***P < 0.001. **d** Box plots depicting the Euclidean distance of CCF of pairwise samples in each patient. Wilcoxon rank sum test, ***P < 0.001

We applied a 2-dimensional Dirichlet process to cluster mutations from paired samples according to their purities and allelic copy numbers [35]. The cluster results were integrated into the phylogenetic trees at the sample level to indicate the clonal relationships among all samples in each patient. For example, patient P10 had two separate foci of invasive carcinoma. We sampled 2 EBVaGCs (P10-C1 and P10-C3) from the larger foci and 1 EBVaGC (P10-C2) from the smaller one. Meanwhile, we obtained 3 dysplasia samples (including 1 HD and 2 LDs) and 1 sample of normal tissue from the area surrounding the tumors (Fig. 4a). Interestingly, 34 mutations from the subclonal cluster (mean CCF, 0.32) in P10-LD2, including a missense mutation in *CTNNB1* (p.D32N), were found to be fully clonal in 4 histological advanced samples (P10-C1, P10-C2, P10-C3, and P10-HD) (Fig. 4b). However, these 4 samples had distant genetic relationships (mean HI, 0.82), which might have been the result of their separated spatial locations. This finding strongly indicated that cells carrying subclonal mutations in P10-LD2 had the same ancestor with those clones in EBVaGCs and HD regions. In addition, P10-C3 contained a subpopulation (mean CCF, 0.23) in which 39 mutations were shared by all tumor cells of P10-C1, which was possibly due to subclonal dissemination between P10-C1 and P10-C3 or unbalanced regional distribution of this subclone within the larger tumor foci of P10.

By constructing phylogenetic trees, we found all EBVaGCs from each patient in our cohort were monoclonal, and all 9 HDs sampled from 7 patients were discovered to have the same origin with EBVaGCs. Of the 17 normal tissues and 19 LDs, only 1 normal tissue and 6 LDs from 7 patients harbored subclones (mean CCF value 0.21–0.54) that were full clones in their matched HDs and EBVaGCs (Fig. 4a, Additional file 1: Figure S5). By calculating the heterogeneity index (HI, proportion of heterogeneous mutations relative to the total mutations of paired samples in each patient), we found that the HI values between EBVaGC-EBVaGC pairs were significantly lower than those between EBVaGC and precursor lesion pairs (EBVaGC-HD pairs, *P* = 5.83e−09; EBVaGC-LD/N pairs, *P* = 2.2e−16) (Fig. 4c). The evident difference between the HI values of EBVaGC-EBVaGC pairs and EBVaGC-HD pairs suggested that, although HDs and EBVaGCs were monoclonal, the former diverged from the latter relatively early. Notably, a comparison of the Euclidean distances of mutation CCF values from paired samples showed consistent results, with the exception that LD/N-LD/N pairs had distance values that were lower than those of other kinds of pairs (*P* < 0.001 for each kind) (Fig. 4d). This difference may have been the result of the lower frequencies of mutations and lower CCF values of mutations in normal tissues and LDs, which had not developed for as much time as had HDs and EBVaGCs.

### Multi-strain infection of EBV within the same patient

**Fig. 5:**
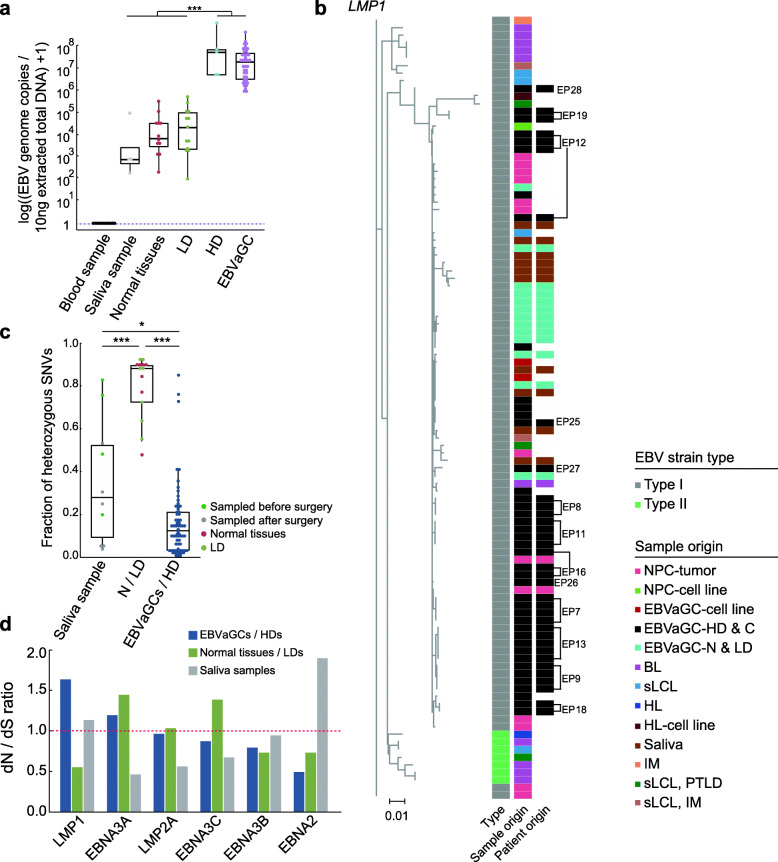
Analysis of EBV genomes in the saliva, normal tissues/LDs, and EBVaGCs/HDs. **a** Box plots comparing the EBV genome copies in peripheral blood samples (*n* = 13), saliva samples (*n* = 4), normal tissues (*n* = 12), LDs (*n* = 13), HDs (*n* = 5), and EBVaGCs (39). The *y*-axis is shown on a log10-transformed scale. Wilcoxon rank sum test, ***P < 0.001. **b** Left: phylogenetic tree of *LMP1* nucleotide sequence from EBV genomes. Right: heatmaps displaying the EBV strain type, sample origin, and patient origin. For HD and EBVaGC samples from the same patients, patient IDs were denoted beside the heatmap. The scale bar of the phylogenetic tree represents 0.01 nucleotide substitution per site. IM, infectious mononucleosis; sLCL, spontaneous lymphoblastoid cell line; EBVaGC, EBV-associated gastric carcinoma; NPC, nasopharyngeal carcinoma; BL, Burkitt’s lymphoma; HL, Hodgkin’s lymphoma; PTLD, post-transplant lymphoproliferative disease. **c** Box plots comparing the fractions of heterozygous single nucleotide variations (SNVs) on EBV genomes from the saliva samples (*n* = 10), normal tissue/LDs (*n* = 14), and EBVaGCs/HDs (*n* = 70). Wilcoxon rank sum test, *P < 0.05, ***P < 0.001. **d** Bar plots displaying the dN/dS ratios of the selected genes of EBV genomes in the saliva samples, normal tissue/LDs, and EBVaGCs/HDs. The dashed line indicates a dN/dS value of 1

We measured the quantity of virus and found the number of genome copies of EBV in EBVaGCs (median, 1.15e07 copies) and HDs (median, 4.03e07 copies) per 10 ng extracted total DNA was significantly higher than that in LDs (median, 5964 copies) and normal tissues (median, 4622 copies) (*P* = 6.89e−12) (Fig. 5a), which demonstrated that rapid expansion of EBV was established at the pre-invasive stage of HD. We then captured the EBV genome from different samples and subjected them to sequencing. A total of 80 EBV genomes from 7 normal tissues, 7 LDs, 5 HDs, and 61 EBVaGCs from 19 patients were analyzed (Additional file 1: Table S2). The results of the alignment of the EBV genome from each sample to multiple EBV reference genomes were consistent among EBVaGCs/HDs within the same patient, but different with that of normal tissues and LDs (Additional file 1: Table S4). By constructing the phylogenetic tree based on EBV *LMP1* gene sequences, we found that the EBV samples from EBVaGCs and HDs were clustered together (Fig. 5b), but divergent from that in normal tissues and LDs of the same patient. Furthermore, EBV sequences from normal tissues and LDs tended to be similar across patients (Fig. 5b, Additional file 1: Figure S6). In addition, we obtained saliva samples from 10 patients and found these samples had a comparable number of EBV genome copies (median, 841 copies) with that of LDs (*P* = 0.13) and normal tissues (*P* = 0.17) (Fig. 5a). We compared the EBV genome from a site of physiological infection to a site of tumorous transformation within the same patients. Four saliva-isolated EBV genomes were aligned to the reference genome LN824142.1, which was obtained from the saliva of a healthy individual in the UK [18]. When comparing the EBV genomes from EBVaGCs and saliva of the same patients, we found that they were genetically distinct, but the latter tended to be clustered together with those EBV genomes from normal tissues and LDs (Fig. 5b, Additional file 1: Figure S6). Of note, by further examining the results of *EBER* RISH in normal tissues and LDs, we identified EBV is present in a few infected B cells (Additional file 1: Figure S7a). Taken together, all these results suggest that the low-abundant EBV we captured from normal tissues and LDs were deposited in those rare infected B cells and not from epithelial cells. In comparison with the same strain-originated EBV within EBVaGCs and neighboring HDs, the EBV genomes from normal tissues and LDs showed evident divergence.

The fractions of heterozygous single nucleotide variations (SNVs) on the EBV genomes from normal tissues/LDs (median, 87.3%) were significantly higher than those of the saliva samples (median, 27.9%) and EBVaGCs/HDs (median, 12.5%) (*P* = 0.029) (Fig. 5c). The greater variability in the sequences of EBV in normal tissues/LDs reflected the potential presence of a mixture of multiple strains or inter-strain recombination [18]. But the limited number of private SNVs on EBV genomes across EBVaGCs and HDs within each patient further consolidates that they harbor single-strain-originated EBV and implies the virus evolved limitedly within the lifetime of tumor development (Additional file 1: Figure S7b). We only observed that EBV genomes from patient 13 had a higher number of private SNVs in tumor samples (ranging from 8 to 37), which might result from unexpected technical artifacts (eg, methylation-dependent DNA damage during formalin fixation). The diversity of EBV sequences in normal tissues/LDs and EBVaGCs/HDs may illuminate the nature of specific strain selection in the tumor samples. We then performed selection analysis on different sample types and identified *LMP1* (dN/dS = 1.64) and *EBNA3A* (dN/dS = 1.20) that evolved under positive selection from the EBV sequences from EBVaGCs and HDs (Fig. 5d). We also found *EBNA3A* (dN/dS = 1.45) and *EBNA3C* (dN/dS = 1.58) in normal tissues/LDs have evolved under positive selection. *EBNA3* family genes (*EBNA3A*, *EBNA3B*, and *EBNA3C*) are latent genes and harbor several known T cell epitopes, which have been identified as targets of cytotoxic T lymphocytes [36, 37]. The observation that these genes evolve under positive selection in gastric tissues may reflect the evasion by EBV-infected cells from the surveillance of the immune microenvironment. We also verified that these genes are highly expressed in EBVaGCs samples by RNA sequencing (Additional file 1: Figure S7c, Additional file 1: Table S8). The finding of the high activity of these virus genes is consistent with the results reported in the TCGA cohort [5], highlighting the potential selection and essential roles of specific virus genes during the tumor development of EBVaGC.

Considering that *Helicobacter pylori* (HP) infection would increase the risk of metaplasia developing to invasive cancer, we also assessed the presence of *Helicobacter pylori* (HP) by Giemsa staining (Additional file 1: Figure S7d) [32]. With the exception of 4 patients (P4, P9, P16, and P20), we did not find HP signals in gastric non-tumorous mucosa. On the other hand, EBVaGCs and HDs had no positive staining signals, suggesting the low ratio of co-infection of HP and EBV in EBVaGCs from our cohort.

### Epigenomic profiling of EBV and tumor cells from precursor lesions to EBVaGCs

**Fig. 6:**
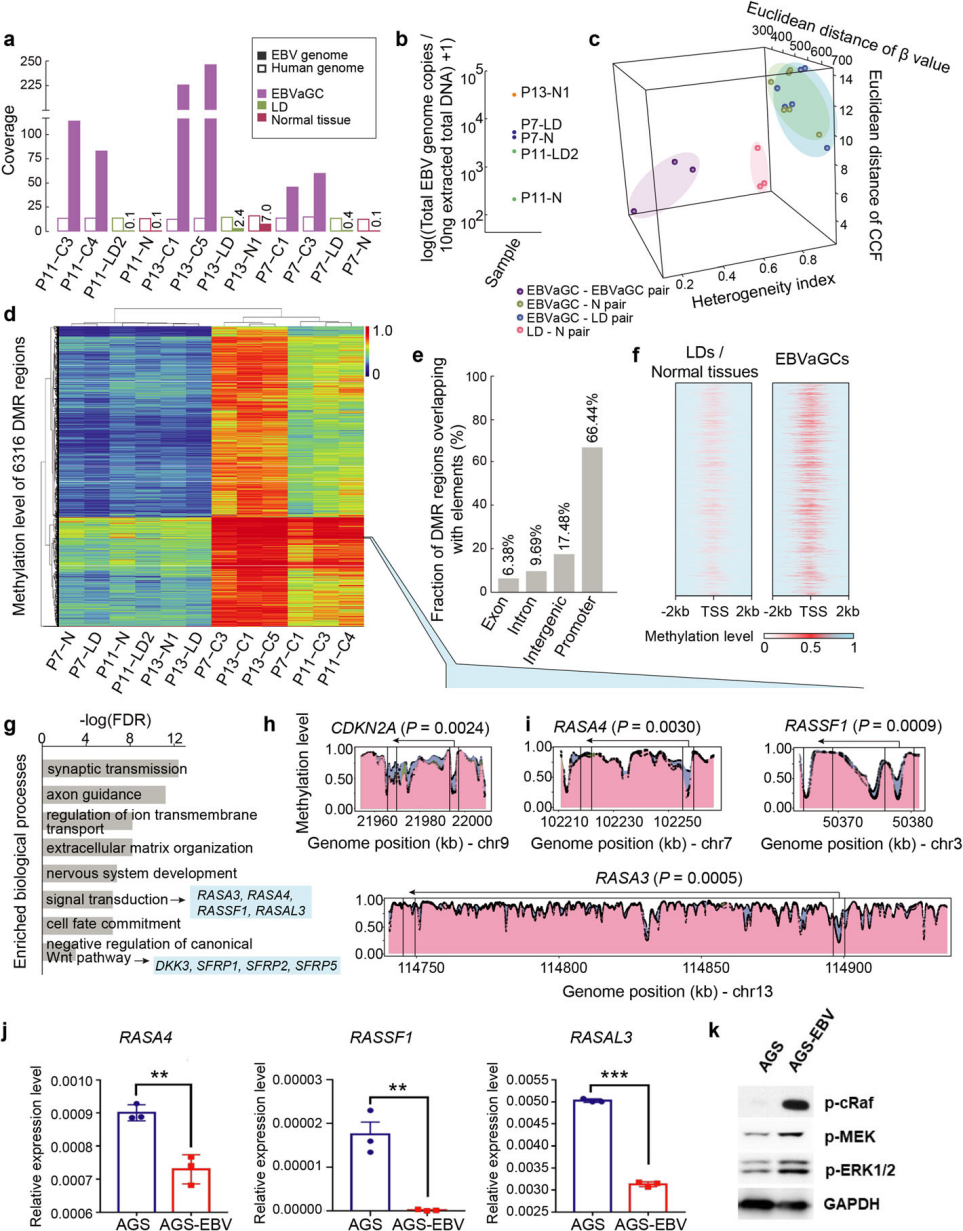
Epigenomic profiling of EBV and host cells in EBVaGCs and their precursor lesions. **a** Bar plots showing the mean coverage of human (hollow bar) and EBV genomes (solid bar) in each sample. Histological types of samples are shown in different colors. **b** Dot plots of EBV genome copies in 5 normal tissues and LDs used for whole-genome bisulfite sequencing (WGBS). The LD sample in patient P13 (P13-LD) is not shown due to the lack of DNA for measurement of EBV genome copies by qPCR (see the “Methods” section). **c** Three-dimensional diagram showing the heterogeneity index, Euclidean distance of CCF, and Euclidean distance of methylation level (*β* value) between pairwise samples in each patient. All pairwise samples are divided into 4 groups. **d** Heatmap of 6316 differentially methylated regions (DMRs) between EBVaGCs and precursor lesions. **e** Bar plots of the fractions of DMRs overlapping with different genomic elements. **f** Diagram exhibiting the methylation level of DMRs encompassed by the region of transcript start site (TSS) ± 2000 bp in LDs and normal tissues (left) and EBVaGCs (right). **g** Enrichment of biological processes (Gene Ontology) for genes with hypermethylated promoters. Genes of interests were selected and indicated. **h** The promoter region of *CDKN2A* showing hypermethylation in EBVaGCs in comparison with that in normal tissues and LDs. Black dots represent each CpG site. Different colors of the areas indicate the histological types of samples (pink, normal tissues; green, LDs; blue, EBVaGCs). The transcription strand is indicated by the arrow orientation (left, reverse strand; right, forward strand). The statistical significance is shown (Student’s *t* test). **i** Genes encoding Ras GTPase-activating proteins (RasGAP) showing hypermethylated promotors in EBVaGCs in comparison with that in normal tissues and LDs. **j** Bar plots comparing the mRNA expression levels of *RASA4*, *RASSF1A*, and *RASAL3* in AGS cell line with or without EBV infection. Student’s *t* test, **P < 0.01, ***P < 0.001. Data are shown as mean ± SD. **k** Western blotting of p-cRaf, p-MEK, and p-ERK1/2 in AGS cell line with or without EBV infection

The high CpG island methylator phenotype (CIMP) is a striking molecular feature of EBVaGCs. Promoter silencing of tumor suppressor genes caused by DNA hypermethylation is believed to drive tumor development [5]. Moreover, it has been reported that *LMP2A* in the EBV genome could cause hypermethylation by activating *DNMT1* and *DNMT3B* [38]. To further characterize the epigenetic changes during tumor evolution, we applied whole-genome bisulfite sequencing to 6 EBVaGCs, 3 LDs, and 3 normal tissues from 3 patients (Additional file 1: Table S2). By uniquely mapping sequencing reads, we found that the mean coverage of the EBV genome in EBVaGCs (median, 97.7) was higher than that in LDs (median, 0.12) or normal tissues (median, 0.45) (Fig. 6a, b).

The pairwise distance of the global methylation level (*β* value) between 2 samples from the same patient showed concordance with the corresponding pairwise HI value (Spearman correlation, *r* = 0.56, *P* = 0.017) and mutation CCF distance (*r* = 0.62, *P* = 0.007) (Fig. 6c), which suggested the relatedness between genomic and epigenomic alterations, as previously observed in glioma [39]. The *β* value distance between EBVaGC-LD pairs (median, 566.2) and EBVaGC-N pairs (median, 542.4) was higher than that between EBVaGC-EBVaGC pairs (median, 302.4; *P* = 0.05) and LD/N-LD/N pairs (median, 268.7; *P* = 0.004). This finding showed that the methylation profiles of normal tissues and LDs were relatively similar, but both were significantly different from those of EBVaGCs. We identified 6316 differentially methylated regions (DMRs) in EBVaGCs relative to normal tissues/LDs, of which 99.8% (6306/6316) were hypermethylated (Fig. 6d). By assigning these hypermethylated regions to different genome compartments, we found that 66.44% of all hypermethylated regions overlapped with the promoter regions (Fig. 6e, f). We then performed pathway analysis to explore the biological relevance of these regions and found significant enrichment of functional clusters, including signal transduction and negative regulation of the Wnt pathway (Fig. 6g). We confirmed hypermethylated (*CDKN2A*) and constitutively unmethylated (*MLH1*) promoters in EBVaGCs as previously reported (Fig. 6h, Additional file 1: Figure S8a). Furthermore, several genes belonging to the Ras GTPase-activating protein (RasGAP) family, including *RASA3* (*P* = 0.0005), *RASA4* (*P* = 0.003), and *RASAL3* (*P* = 0.0016), were identified with hypermethylated promotors in EBVaGCs compared with that in precursor lesions and other gastric cancers (Fig. 6i, Additional file 1: Figures S8b and S9). Together with the gene *RASSF1*, as previously identified in the context of hypermethylation in EBVaGCs [40], silencing of these genes by methylation could increase the abundance of the active form of Ras and induce downstream signaling, such as activation of the PI3K-Akt pathway, to further drive tumor development. To validate this finding, we compared the methylation levels and expressions of RasGAP family genes in the gastric cancer cell line AGS before and after EBV infection. We identified the hypermethylation and reduced the mRNA expressions of RasGAP family genes and increased RAS pathway activity in AGS cells after EBV infection (Fig. 6j, k, Additional file 1: Figure S10a, Additional file 2: Figure S14). These results suggested that EBV infection might reduce the expressions of these genes. The treatment with 5-Aza-2′-deoxycytidine (5-aza), a DNA methylation inhibitor, could rescue the expressions of RasGAP family genes (Additional file 1: Figure S10b). Furthermore, the cell proliferation and clonogenicity abilities in EBV-positive GC cells were inhibited by the treatment of 5-aza (Additional file 1: Figure S10c-f).

We also discovered several genes from the Wnt pathway with hypermethylation in EBVaGCs relative to LDs/normal tissues, including *DKK3* (*P* = 0.0022), *SFRP1* (*P* = 0.0034), and *SFRP2* (*P* = 0.0005) (Additional file 1: Figure S8c), and these genes have been reported to be frequently methylated and to function as tumor suppressors in NPCs [41]. Considering the effects of potential artifacts brought by low-coverage of WGBS data and formalin damages on DNA in FFPE samples, we also applied reduced representation bisulfite sequencing (RRBS) to fresh frozen normal tissues and EBVaGC samples from 3 patients to validate the above findings (Additional file 1: Figure S9, Additional file 1: Table S8). The consistent results further consolidated the hypermethylated profile and essential functions of RasGAP genes and Wnt regulator genes in EBVaGCs.

We compared the methylation level of EBV in the normal tissue and LD sample of P13 (EP13-LD and EP13-N1) with that in 2 EBVaGCs of the same patient (EP13-C1 and EP13-C5). The global methylation level of EBV was high in both EBVaGCs and their early-stage precursor lesions, with more than 93.5% and 94.5% of CpG sites having a *β* value higher than 0.8, respectively (Additional file 1: Figure S8d). We also discovered unmethylated regions of plasmid replication (oriP), Q promoter (Qp), and hypermethylated C promoter (Cp), consistent with the previous study (Additional file 1: Figure S8e) [42]. These results imply a consistent hypermethylation profiling of EBV from normal tissues and LDs to EBVaGCs and warrant further consolidation in a larger dataset.

### PI3K and Wnt pathways synergistically activate clonal expansion of EBV-infected cells

**Fig. 7:**
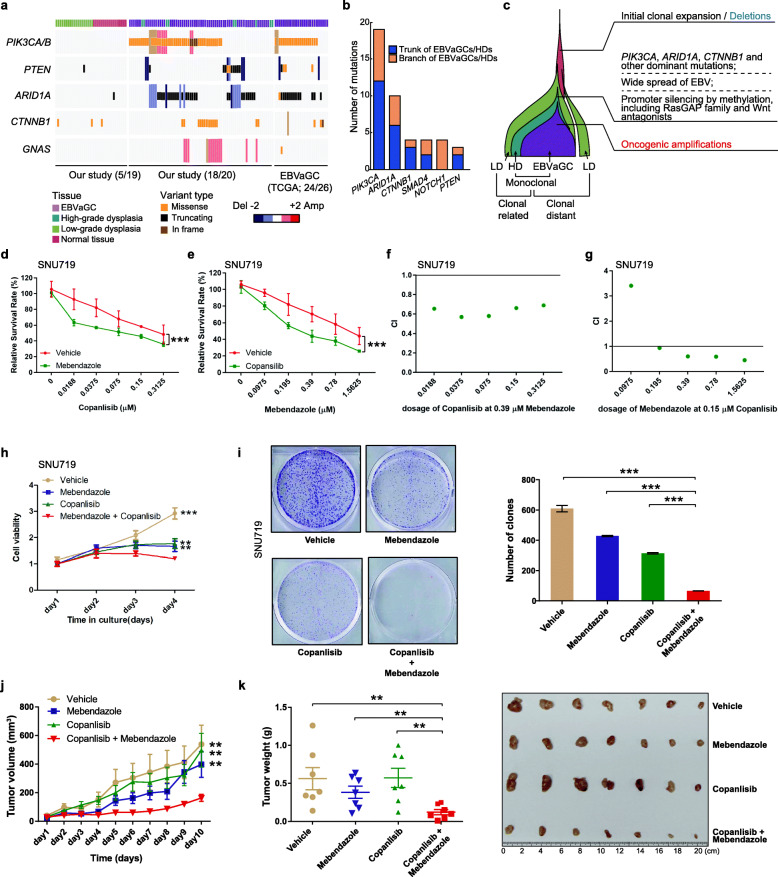
Synergistic function of the PI3K-Akt and Wnt pathways in EBVaGCs. **a** Heatmap of the recurrently mutated genes in the PI3K and Wnt pathways in precursor lesions (left) and EBVaGCs (middle, our study; right, TCGA data). The histological types of each sample are indicated on the top. **b** Bar plots displaying the number of “trunk” (gray) or “branch” (green) mutations in the indicated driver genes. **c** Schematics summarizing the evolutionary process of EBVaGC. Key events promoting the developmental process are denoted. **d** The growth curve of SNU719 cells treated with the indicated doses of PI3K inhibitor (copanlisib) at the presence of Wnt pathway inhibitor (0.39 μM mebendazole) or vehicle (*n* = 3). **e** The growth curve of SNU719 cells treated with the indicated doses of Wnt pathway inhibitor (mebendazole) at the presence of PI3K inhibitor (0.15 μM copanlisib) or vehicle (*n* = 3). **f** The CI of SNU719 cells treated with the indicated doses of copanlisib at the presence of 0.39 μM mebendazole. **g** The CI of SNU719 cells treated with the indicated doses of mebendazole at the presence of 0.15 μM copanlisib. **h** The growth curve of SNU719 cells treated with either 0.5 μM copanlisib or 0.3 μM mebendazole, or the combination (*n* = 3). **i** Comparison of colony formation of SNU719 cells with different treatments or vehicle (*n* = 3). **j** Tumor growth curve of SNU719 xenografts with different treatments (copanlisib 6 mg/kg, mebendazole 20 mg/kg) or vehicle (7 mice per group). **k** Tumor weight of SNU719 xenografts with different treatments or vehicle (7 mice per group). All statistics were conducted using Student’s *t* test, *P < 0.05, **P < 0.01, ***P < 0.001. Data are shown as mean ± SD

During tumor development, the dominant cellular lineages in monoclonal HDs and EBVaGCs harbored clonal mutations and/or CNAs targeting the PI3K-Akt pathway (*PIK3CA*/*B* and *PTEN*), the Wnt pathway (*CTNNB1* and *GNAS*) [43], and *ARID1A* (our study, 18/20 patients; TCGA, 24/26 EBVaGC s[5]), whereas these genetic changes were rare in normal tissues and LDs (our study, 5/19 patients) (*P* = 3.93e−07) (Figs. 2b and 7a). As a subunit of the SWI/SNF chromatin remodeling complex, loss-of-function of *ARID1A* has also been revealed to be involved in the activation of PI3K-Akt signaling via antagonism of high *EZH2* expression [44]. Compared with other driver genes, mutations in *PIK3CA*, *ARID1A*, and *CTNNB1* have a bias towards being truncal events of EBVaGCs and HDs across patients (Fig. 7b). Consequently, in combination with epigenomic changes that also selectively target negative regulators of Ras upstream of PI3K-Akt and Wnt antagonists, we hypothesized that the PI3K-Akt and Wnt pathways functioned collectively to activate clonal expansion of EBV-infected cells in HDs and EBVaGCs and promoted the tumor evolution (Fig. 7c).

Two EBVaGC cell lines were used to perform *in vitro* experiments to confirm this hypothesis. Of these 2 cell lines, SNU719 and AGS cells simultaneously harbored *PIK3CA* and *CTNNB1* mutations (Additional file 1: Table S9). We treated SNU719 cells and AGS cells carrying EBV stable infection (AGS-EBV) with PI3K inhibitors (copanlisib or LY294002) and Wnt pathway inhibitors (mebendazole or ICG001). The IC50 value of copanlisib was dramatically reduced from 0.2808 to 0.0637 μM when it was combined with mebendazole in SNU719 cells (Fig. 7d). The IC50 value of mebendazole was decreased from 1.497 to 0.3578 μM when it was combined with copanlisib in SNU719 cells (Fig. 7e). We further calculated the combination index (CI) and found the CI between copanlisib and mebendazole was less than 1, suggesting that there is a strong synergy between these two drugs (Fig. 7f, g). Consistently, treatment with a combination of copanlisib and mebendazole significantly reduced cell proliferation of SNU719 cells by MTT assay, compared to vehicle, copanlisib, or mebendazole alone (Fig. 7h). Similarly, the treatment of LY294002 and ICG001 had synergistic anti-proliferative effects on SNU719 and AGS-EBV cells (Additional file 1: Figure S11a-b). We found that either copanlisib or mebendazole alone can reduce the colonigenicity, whereas the combination further inhibited the colonigenicity abilities of SNU719 cells (Fig. 7i). Likewise, AGS-EBV cells treated with the combination of the PI3K inhibitor and Wnt pathway inhibitors presented the lowest abilities of colony formation (Additional file 1: Figure S11c). Taken together, our data suggested the synergistic repressive effects of the PI3K inhibitor and Wnt pathway inhibitor on cell proliferation and clonogenicity* in vitro*.

In addition, we found the treatment of copanlisib and the combination significantly reduced the levels of p-Akt (Additional file 1: Figure S11d, Additional file 2: Figure S15), suggesting the successful inhibition of the PI3K-Akt pathway by copanlisib. The activity of TOPflash was suppressed by the treatment of mebendazole or the combination, indicating the specific inhibition on TCF4 activities by mebendazole (Additional file 1: Figure S11e). These results further confirmed the mechanisms of action by copanlisib and mebendazole.

To investigate the influence of the combination therapies of copanlisib and mebendazole on tumorigenesis *in vivo*, the mice bearing SNU719 xenografts were established and then treated with copanlisib with intraperitoneal injection at 6 mg/kg Q2D, mebendazole with 20 mg/kg oral administration daily, and the combination of these two drugs, in comparison with the vehicle. While copanlisib or mebendazole alone only showed no or slightly therapeutic effects, the combination therapy showed significant effect as measured by tumor growth, tumor weight, and survival (Fig. 7j, h, Additional file 1: Figure S11f), suggesting synergetic therapeutic effect by the combination of PI3K inhibitor and Wnt pathway inhibitor. No significant weight loss was seen with the drug alone or the combination.

Meanwhile, a gastric cancer cell line HGC27 which harbors only a *PIK3CA* mutation and two other gastric cancer cell lines SNU16 and SNU5 lacking *PIK3CA* and *CTNNB1* mutations were used to test the specificity of synergistic effects of copanlisib and mebendazole. As shown in Additional file 1: Figure S12a-b, the combination of copanlisib and mebendazole did not show better treatment effects in these three cell lines (HGC27, SNU16, and SNU5) compared to the single drug. The CI in these cell lines were all higher than 1 (Additional file 1: Figure S12c-d), indicating no synergistic effects of these two drugs in these cell lines. Moreover, we found that EBV infection did not influence the sensitivities of these two drugs by comparing the treatment efficacy in AGS-EBV and AGS cell lines (Additional file 1: Figure S13). These data strengthened the finding that the combination of these two drugs could increase the therapeutic efficacy when cells harbor both *PIK3CA* and *CTNNB1* mutations and implied a broader application not specifically to EBVaGCs.

Taken together, these results suggested a synergistic effect of the PI3K-Akt and Wnt pathways on cell growth and invasion, and a combination of inhibitors for these two pathways demonstrated potential as a treatment strategy for EBVaGCs.

## Discussion

Our study portrayed the genomic landscape of tumor-neighboring morphologically normal tissues and dysplasia samples of EBVaGCs. According to our data, the mutational burden significantly increases from normal tissues and LDs to HDs and EBVaGCs. The extensive heterogeneity between EBVaGCs and their precursor lesions, especially normal tissues and LDs, within the same patients, reveals the diversified mutational background underpinning the initiation of EBVaGCs. On the other hand, we also observed normal tissues or LDs as direct cellular precursors to matched neighboring HDs and EBVaGCs, whereby minor clones carrying driver mutations in the former disseminate early, predisposing cells to acquire additional mutations in driver genes, like *PIK3CA* and *ARID1A*, and eventually give rise to the dominant clonal lineage in histological advanced samples. The results of our comparison of EBVaGCs and their precursor lesions indicate that deletions tend to occur early during the development of EBVaGCs, but oncogenic amplifications typically emerge late with progression to EBVaGCs. A similar scenario, in which tumor cells appear to follow an evolutionary path with the late occurrence of oncogenic amplifications, has also been discovered in gastrointestinal adenocarcinoma EACs [14, 45].

In the present study, we used *EBER* RISH, specific probe real-time PCR, and sequencing, to illuminate EBV load dynamics during the development of EBVaGCs. First, all three methods identified substantial EBV in HDs, suggesting that widespread infection by EBV is established at this pre-invasive stage. Second, our results suggest the presence of a very low-level gain of EBV in tumor-neighboring normal tissues and LDs, and the *EBER* RISH showed the limited EBV is from a few infected B cells rather than epithelial cells. Within the same patients, all EBVaGCs and HDs were monoclonal and contained the same single-strain-originated EBV, which suggested that lesions with EBV infection may arise from the clonal proliferation of a single infected epithelial cell. However, for a subset of normal tissues and LDs that already have minor clonal expansion (21–54% of the total cells) and are cellular ancestors of matched HDs and EBVaGCs, the detection of very few EBV copies indicates that the growth of proliferated clones in normal tissues and LDs is not initiated by a virus-infected cell, suggesting that EBV maybe not the initiator for the development of EBVaGCs [7].

Regarding the sequence of EBV, the diversity of EBV genomes among individuals has been elucidated through cohort studies [18]. Genomic differences, especially those located within genes encoding transforming viral proteins, could have large effects on the phenotypes of EBV and host cells [46]. In our study, we identified substantial genomic differences in EBV within patients by comparing viral sequences from multiple sites, including saliva, normal tissues, LDs, HDs, and EBVaGCs. We found that *EBNA3* family genes of EBV from gastric tissues evolve under positive selection, suggesting these genes are involved in immune evasion by EBV-infected cells [36, 37]. The EBV sequence in the saliva was observed to be variable, but the originating tissues (tonsil and/or salivary gland) and cells from which the virus was shed remain unknown [47, 48]. Thus, although we found that *EBNA2* in the saliva were under positive selection, and the dN/dS ratio of this gene showed sharp contrast among saliva, normal tissues/LDs, and EBVaGCs/HDs, the reason underlying this is unclear. However, on the basis of the recognition of distinct EBV genotypes in different sites within the same individual, a better comprehensive definition of EBV diversity between tumors and other tissues will deepen our understanding of the role of EBV variations in tumor development [49].

We identified numerous regions in which the degree of hypermethylation increased from normal tissues and LDs to EBVaGCs. Such epigenetic changes in EBVaGCs are potentially associated with the widespread of EBV showing high expression of *LMP2A*, which is able to activate *DNMT1* and *DNMT3B* in host cells [38]. Supporting this interpretation, a number of tumor suppressor genes (TSGs) with hypermethylation at promoter regions, including those of several Wnt antagonists and RasGAP family members, were identified. In addition, we also observed significantly more genomic alterations in EBVaGCs and HDs in comparison with normal tissues and LDs targeting the PI3K-Akt and Wnt pathways. *PIK3CA* mutations in different domains such as adaptor-binding domain (ABD), C2 domain, and kinase domains have been frequently detected in different cancer types [50, 51]. The hot-spot mutations in exon 3 of *CTNNB1*, which might change the phosphorylation status of β-catenin, were mainly presented in colorectal cancer and other cancers [52]. It has been reported that the therapeutic resistance from PI3K inhibitor might result from the activation of the Wnt-β-catenin pathway. There was an additive inhibitory effect with both PI3K inhibitor and Wnt inhibitor on tumor growth of triple-negative breast cancer in mice xenograft models [53]. As synergistic activation of these two pathways could enhance the mitogenic ability of cells, this finding may provide evidence of the requisition of specific genetic changes by EBV to facilitate persistent infection and co-expansion with its infected host cells. By applying combinational treatment with Food and Drug Administration (FDA)-approved PI3K inhibitor copanlisib and Wnt pathway inhibitor mebendazole to EBVaGC cells, we observed a significant inhibitory effect and confirmed the synergistic function of the PI3K-Akt and Wnt pathways in EBV infection and co-expansion.

## Conclusions

We illuminated the genomic landscape of both EBV and host cells from precursor lesions to invasive gastric carcinomas and demonstrated the synergistic activation of the PI3K and Wnt pathways in EBVaGCs, providing a potential new strategy for the clinical management of patients with EBVaGCs.

## Supplementary Information

**Additional file 1.** Supplementary methods, figures (Figures S1-S13) and tables (Tables S1-S9).

**Additional file 2.** Full images of the western blotting results (Fig. S14-S15).

## Data Availability

The raw data of whole-exome sequencing, EBV genome sequencing, and whole-genome bisulfite sequencing of this study have been deposited in the Genome Sequence Archive in BIG Data Center of Beijing Institute of Genomics (BIG), Chinese Academy of Sciences (https://ngdc.cncb.ac.cn/gsa-human/), under the accession number HRA001143 (https://ngdc.cncb.ac.cn/search/?dbId=hra&q=HRA001143) [54]. The raw data of all the functional experiments of the main figures in this work has been deposited onto the Research Data Deposit public platform (www.researchdata.org.cn) with the approval number RDDB2021001631.
