# Supplementary material for: The genomic architecture of EBV and infected gastric tissue from precursor lesions to carcinoma
Source: Genome Med. 2021 Sep 7;13:146. doi: 10.1186/s13073-021-00963-2 (PMC8422682; doi:10.1186/s13073-021-00963-2)
Supplement: Supplementary file 1 — Additional file 1. Supplementary methods, figures (Figures S1-S13) and tables (Tables S1-S9). [file 13073_2021_963_MOESM1_ESM.docx]

**Additional file 1**

**The genomic architecture of EBV and infected gastric tissue from precursor lesions to carcinoma**

Zhang-Hua Chen^1#^, Shu-Mei Yan^2,3#^, Xi-Xi Chen^1#^, Qi Zhang^2,4,8#^, Shang-Xin Liu^2#^, Yang Liu^1^, Yi-Ling Luo^2^, Chao Zhang^6,7^, Miao Xu^2^, Yi-Fan Zhao^1^, Li-Yun Huang^2,3^, Bin-Liu Liu^2^, Tian-Liang Xia^2^, Da-Zhi Xu^5^, Yao Liang^5^, Yong-Ming Chen^5^, Wei Wang^5^, Shu-Qiang Yuan^5^, Hui-Zhong Zhang^2,3^, Jing-Ping Yun^2,3^, Wei-Wei Zhai^9,10^, Mu-Sheng Zeng^2^, Fan Bai^1,11*^, Qian Zhong^2*^

^1^Biomedical Pioneering Innovation Center (BIOPIC), School of Life Sciences, Peking University, Beijing, China.

^2^State Key Laboratory of Oncology in South China, Collaborative Innovation Center for Cancer Medicine, Sun Yat-sen University Cancer Center, Guangzhou, China.

^3^Department of Pathology, Sun Yat-sen University Cancer Center, Guangzhou, China.

^4^Department of Oncology, Second Affiliated Hospital, Guangzhou Medical University, Guangzhou, China.

^5^Department of Gastric Surgery, Sun Yat-sen University Cancer Center, Guangzhou, China.

^6^Institute for Computational Biomedicine, Weill Cornell Medicine, New York, NY 10021, USA

^7^Division of Hematology/Oncology, Department of Medicine, Weill Cornell Medicine, New York, NY 10021, USA

^8^Department of Ultrasound, The Fifth Affiliated Hospital of Sun Yat-sen University, Zhuhai, China.

^9^Key Laboratory of Zoological Systematics and Evolution, Institute of Zoology, Chinese Academy of Sciences, Beijing, China.

^10^Center for Excellence in Animal Evolution and Genetics, Chinese Academy of Sciences, Kunming, China.

^11^Beijing Advanced Innovation Center for Genomics (ICG), Peking University, Beijing, China.

**Methods**

**Patient enrollment**

Collection and publication of human genetic data in this study were approved by the Ministry of Science and Technology of China. This study was approved by the institutional review board of the Sun Yat-Sen University Cancer Center. All samples collected in this study were obtained with informed patient consent. RNA *in situ* hybridization (RISH) analysis was performed on primary gastric tumors using *EBERs* probe to identify Epstein-Barr virus (EBV)-positive tumors. Formalin-fixed paraffin-embedded (FFPE) tissue blocks from patients who underwent resection of primary gastric tumor during the period from December 2013 to December 2016 and received no previous treatment at Sun Yat-Sen University Cancer Center, were retrieved from the Tumor Resource Bank of Sun Yat-Sen University Cancer Center and subjected to *EBERs* RISH examination. Consequently, FFPE blocks from 20 patients were identified with the presence of both EBV-associated gastric carcinomas (EBVaGCs) and precursor lesions. Clinical information, including tumor stage, histological features (Lauren classification) and immunohistochemical results, are summarized in **Additional file 1: Table S1**.

**Sample collection**

Slides stained with hematoxylin and eosin (HE) were reviewed independently by three pathologists to identify the consensus areas of EBVaGC, high-grade dysplasia (HD), low-grade dysplasia (LD) and morphologically normal epithelial tissue neighboring tumors or dysplasias (N). If there existed contradictory opinions, the three pathologists looked at the slides together. In such cases, samples with an agreed consensus were included in our research; otherwise, samples that still remained controversial were excluded. Moderately graded dysplasia samples were categorized as LD in this work due to their limited number. In 20 patients, normal epithelial tissues and dysplasia samples neighboring tumor nests and 2–5 spatially separated regions (at least ≥ 0.5cm away from each other) within each independent tumor nest from each individual were marked. Consequently, a total of 109 distinct marked areas of interest were isolated via laser capture microdissection (LCM) using a Leica LMD7000 Microsystem (Wetzlar). Meanwhile, matched control samples were collected, including 2 distant normal gastric tissues (≥ 5 cm from tumor site; P5 and P13) and 18 blood samples. To investigate the diversity of EBV genomes within each individual, saliva samples (2 mL) from 5 of these 20 patients were also included in this study. Paired EBVaGCs and saliva samples from 5 additional patients without precursor lesions were added to the EBV genome analyses (**Additional file 1: Table S1**). The number and histological type of all collected samples are listed in **Additional file 1: Table S2**. Furthermore, for the key findings in our work, we also utilized paired fresh frozen EBVaGCs and normal tissues to validate by RNA sequencing (2 pairs) and reduced representation bisulfite sequencing (RRBS, 3 pairs), respectively (see main text) (**Additional file 1: Table S8**).

**DNA extraction**

DNA from blood and saliva samples was extracted using the QIAamp DNA Micro Kit (Qiagen) according to the manufacturer’s protocol for small-volume blood samples. FFPE samples that underwent LCM were extracted using the same QIAamp DNA Micro Kit according to the manufacturer’s instructions for LCM tissue, except that UNG enzyme was applied after decrosslinking to remove deaminated cytosines caused by formalin fixation. After extraction, DNA was quantified using the Qubit Fluorometric system (Life Technologies).

**Library construction and sequencing**

Whole exome sequencing: for 107 samples and 20 matched control samples, 50–200 ng DNA was sheared into 300–500 bp fragments using the Covaris Ultrasonic system (Covaris). The fragmented DNA was end-repaired, 5′-phosphorylated and ligated to sequencing adaptors using the Sure Select library preparation kit (Agilent Technologies) following the manufacturer’s instructions. The whole exonic regions of each sample were captured using the Sure Select V6 whole exon kit (Agilent Technologies).

EBV genome sequencing: 7 normal tissues, 7 LDs, 5 HDs, 65 EBVaGCs and 10 saliva samples with a minimum amount of 50 ng DNA were subjected to whole EBV genome capture. DNA was sheared into 300–500 bp fragments, followed by end-repair, 5′-phosphorylation and adaptor ligation using the VAHTS Universal DNA Library Prep Kit (Vazyme). The EBV Enrichment Assay Kit2 (MyGenostics) was used to capture the EBV genomic regions. To reduce the level of sequence contamination from humans and other organisms, hybridization with the EBV genome bait of each HD and EBVaGC sample was processed in two rounds before the final enrichment PCR.

Whole genome bisulfite sequencing: 3 normal epithelial tissues, 3 LDs and 6 EBVaGCs with available DNA (at least 100 ng) from 3 patients (P7, P11 and P13) were subjected to library construction. To validate the efficiency of the bisulfite conversion, unmethylated λ DNA (Promega) was spiked into the samples. The protocol for library construction was a modified version of the Post Bisulfite adaptor tagging (PBAT) method [1]. In brief, genomic DNA was first treated with bisulfite using the EZ DNA Methylation Kit (Zymo Research). Next, the first strand was synthesized using high-concentration Klenow (exo-) (Qiagen). The untagged first strand primer was digested using Exonuclease I (New England Biolabs), after which the first strand product was purified and subjected to second-strand synthesis and adaptor tagging. Finally the product was amplified for subsequent sequencing.

All library products were quality-checked and paired-end sequenced on the Illumina X10 platform. Of note, to eliminate the suboptimal calling of mutations caused by potential uncovered reads across genome in FFPE samples, we generated a minimum data amount of 15G for each sample. By increasing the data quantity, we could achieve higher genome coverage and sequencing depth (average sequencing depths of 102.67-fold and 87.21-fold were achieved for the tumor and control samples, respectively). The sequencing information for all samples is listed in **Additional file 1: Table S3-S5**.

**Multi-sample mutation calling**

For the exome sequence data, paired-end 150bp fastq files were aligned to the reference genome hg19 build (UCSC) using BWA (BWA-0.5.9) with default parameters to generate a binary sequence alignment map (BAM) file [2]. The aligned BAM files were sorted and merged using Samtools 0.1.19 [3]. Duplications of sequencing reads were marked and excluded using Picard tools. All insertions and deletions (INDELs) were realigned, after which the base quality was recalibrated using the Genome Analysis Toolkit (GATK2.1-8) [4]. Next, the BAM file of each sample was independently subjected to somatic mutation calling. We first applied MuTect to identify single nucleotide variations (SNVs) and Indel locator to identify short INDELs [5]. Next, we used Strelka, another somatic mutation calling tool, to detect SNVs and INDELs [6]. Mutations identified by both methods were reserved and annotated by SNPEFF. To ensure the fidelity of all called mutations, we set the following criteria as an additional filter to select reliable somatic mutations: 1) mutations listed in the National Heart, Lung and Blood Institute Exome Sequencing Project were removed; 2) contamination by germline SNPs was minimized by removing mutations deposited in dbSNP 135 unless they were documented in the Catalog of Somatic Mutations in Cancer (COSMIC).

Based on the independent identification of somatic mutations in each sample, we adopted a force calling strategy to improve the sensitivity of mutation calling from multiple samples within the same patient [7]. In brief, for each mutation identified in at least one sample as stated above, we counted the number of reads (read quality ≥ 5) supporting the alternate or reference base (base quality ≥ 20) in other matched samples within the same patient. A mutation was selected if more than 3 reads covered the mutational site and the variant allele frequency (VAF) was higher than 0.02. After mutation calling, we evaluated the extent of mutational heterogeneity based on the heterogeneity index (HI) between each sample pair within the same patient as described in our previous study [8].

**Mutational signature analysis**

Single nucleotide variations (SNVs) could be categorized into 6 directions, namely, C > T, C > A, C > G, T > C, T > G, and T > A. Considering the 5’ and 3’ flanking nucleotides of a specific mutated base, a total of 96 substitution types exist. We first plotted the ‘lego’ plots to compare the frequency of mutations within specific contexts in precursor lesions and EBVaGCs of 20 patients. As the set of mutational contexts of tumor samples was an imprint of the mutational process that shaped the cancer genome, we then performed a mutational signature analysis of all silent and non-silent mutations in our study. To extract the underlying mutational signatures in single precursor lesion samples and EBVaGCs, we applied the R package deconstructSigs [9] to each sample using the 30 signatures documented by the COSMIC as reference. After extraction, we calculated and compared the mean weights of different signatures in 45 precursor lesions and 65 EBVaGCs.

**Determination of putative driver mutations**

To identify putative driver mutations in EBVaGCs and their precursor lesions, we first selected genes on the basis of recent large cohort studies of gastrointestinal malignancy, the COSMIC cancer gene census and the Kyoto Encyclopedia of Genes and Genomes (KEGG) pathways in cancer. Next, the likely driver status of all non-silent mutations in these genes was manually evaluated based on the following three aspects: 1) the exact mutations or mutational sites were in recurrent mutation hotspots; 2) the exact mutations or mutational sites were documented in the COSMIC database (gastric cancer-associated or related to other cancer types); 3) the mutations were predicted to be deleterious (including nonsense, frame shift and splicing mutations). Mutations were classified as putative driver mutations if they matched one of the requirements listed above.

**Copy number analysis and homologous allele tracking**

Copy number analysis of exome sequence data was performed using ReCAPSEG (implemented in GATK v4). In principle, the standard BAM file of each sample was used as input to extract the read depth at the captured regions. Next, the read depth of each sample was normalized to a panel of control samples to reduce noise. The panel of controls was created using all germline samples collected for this study. The normalized depth ratios of each sample were segmented based on a circular binary segmentation algorithm. Next, informative germline SNPs documented by dbSNP 135 were integrated into the segments to derive allelic copy number ratios. The allelic copy number data was paired with the mutations and used as input to generate solutions in ABSOLUTE, including: 1) purity; 2) average ploidy; 3) the presence of whole genome doubling events; and 4) the absolute allelic copy number across the genome of each sample [10]. Germline SNPs on sex chromosomes were excluded from this analysis. The processed segment data of the samples were divided by the ploidy to identify copy number alterations (CNAs). Amplifications and deletions were defined as genes for which the total copy number deviated from the baseline ploidy by more than 1 copy. To compare the overall copy number states of EBVaGCs and precursor lesions in a whole-genome context, we performed a permutation test to empirically determine the significance of CNAs at different locations across the genome in EBVaGCs and precursor lesions. Considering that such a comparison would be limited to arm- and chromosome-level CNAs, we compared the copy number status of putative driver genes (selected using the method described above) from samples at different histological stages.

To identify loss of heterozygosity (LOH) events in each sample, we inspected the major and minor allele copy numbers calculated by ABSOLUTE. To determine whether the occurrence of LOH in multiple samples within one patient resulted from the same allele loss event, we performed an analysis to track homologous segments [11]. First, we calculated the allele frequency of germline SNPs by adjusting the purity of each sample and capture bias as follows:

$$MAFadjmin= \frac{MAFobsmin-a*(1-p)}{p}$$

$$MAFadjmax=1-MAFadjmin$$

Where *MAFadjmin* and *MAFadjmax* represented the adjusted minor and major allele frequency at each germline SNP site, *MAFobsmin* denoted the observed minor allele frequency, and *p* represented the purity of the sample. We used the variable *a* to reduce the capture bias for different alleles. If the observed minor allele was a reference allele, *a* was set to 0.516; otherwise *a* was set to 0.484. Based on the adjusted results, the major and minor allele in one sample was defined as a reference or mutant allele, respectively. Next, we applied this calculation to other samples within the same patient to classify and tag major and minor alleles as reference or mutant alleles. Based on the established tags, we were able to track whether the lost alleles in a specific LOH region across multiple samples within the same patient were homologous.

**Cancer cell fraction clustering**

For each somatic mutation, the cancer cell fraction (CCF) was calculated by ABSOLUTE by integrating the purity of the sample, local copy number, and allele counts. In addition, we conducted an analysis to cluster mutations using the PyClone method [12]. In principle, the assumption that each sample had a number of distinct populations carrying different mutations allowed us to infer: 1) the number of clones that co-existed within the sample; 2) the clonality of different clones; and 3) the number of mutations within each clone. For each mutation, we used the binomial distribution to model the observed mutant read counts. The fraction of cells carrying a particular mutation was determined with a Dirichlet process with a base distribution of U (0, 1). The Markov chain Monte Carlo (MCMC) sampling method was adopted to obtain the posterior distribution of the parameters. The MCMC chain was run for 5000 iterations, and the initial 500 iterations were treated as a burn-in period and discarded. In each sample, a particular mutation was considered as subclonal if the defined clone comprising this mutation had a mean CCF value less than 0.8.

**Construction of phylogenetic trees**

For each patient, a phylogenetic tree was constructed using both mutations and CNAs. First, consistent with the method used in our previous study [8], we extracted a sequence encompassing the mutations (total length of 21 bp) to infer the phylogeny among samples from each patient based on the maximum likelihood algorithm. The original phylogenetic tree explained the presence and absence of most mutations among samples in each patient, which also directly reflected the extent of relatedness among samples. In this step, there existed a small proportion of mutations that did not fit the phylogenetic tree of each patient, perhaps because of the absence of mutations due to copy number loss, a low level of clone mixing, or technical noise.

The assignment of CNAs to phylogenetic trees was conducted by checking the breakpoints (start and end) and allelic copy numbers of altered segments. Considering that breakpoint identification was affected by the exome capture of different samples in each patient, we set a tolerance value of 500 bp when we judged whether one CNA event harbored the same breakpoints in multiple samples. When assigning LOH events to the phylogenetic trees, the results of homologous tracking (as described above) were also taken into account. In our results, we did not identify any 2 (or multiple) samples within the same patient that had lost opposite alleles in a breakpoint-identical LOH region. After assignment of CNAs, the length of each line on the phylogenetic trees was adjusted to be proportional to the number of mutations and CNAs.

Next, we refined the phylogenetic trees by incorporating the CCF clustering results, which reflected the clonal relationships among all sampled regions in each patient. In detail, we performed CCF clustering at 2 dimensions for any 2 samples within the same patient. Shared clonal mutations of multiple samples and private mutations in a single sample were directly assigned to the original phylogenetic trees. Shared subclonal mutations that were fully clonal in one sample, but were subclonal in the other sample or in both samples, were specially considered. In our study, we set strict criteria to define a shared subclone based on the clustering results; the shared subclone was required to have at least 10 mutations, in which a minimum number of 3 mutations harbored more than 3 reads supporting the mutant base. Consequently, shared subclonal mutations that were not involved in a defined subclone were excluded from the phylogenetic trees. Notably, as previously recognized, a subclone shared with two samples should be distinguished from common shared ancestry [13]. Thus, if 1 sample shared a subclone with other samples within the same patient, the branch position of this sample on the phylogenetic tree was readjusted to indicate those shared subclonal mutations. The position and mutations contained within the shared subclone were also marked. Therefore, by integrating the CCF clustering results, modified phylogenetic trees reflected the evolutionary process of different clonal lineages in each patient at the sample level.

**EBV genome alignment, SNV calling and construction of phylogenetic trees**

For each EBV genome sequence, paired-end data were first realigned to a reference panel composed of 67 complete EBV genomes downloaded from the NCBI using BWA. These 67 references covered EBV genomes from different geographical origins and cancer types (including one saliva sample from a healthy individual) (**Additional file 1: Table S6**) [14]. Furthermore, the potential capture bias resulting from the differences between type 1 and type 2 EBV was not observed in our results. Based on the realigned EBV sequences, we ranked all reference genomes by counting the number of uniquely mapped paired-end reads with high quality (mapping quality ≥ 20 and length ≥ 50bp) and selected the top ranked EBV reference genome as the most probable origin of the EBV genome in each sample. Next, for each sample, we realigned the EBV sequence data to the specific reference genome using BWA. The average proportion of paired reads that mapped to the reference genome was 82.68% (**Additional file 1: Table S4**). The realigned BAM files were marked and sorted, after which duplicates were removed.

The processed BAM files of the EBV sequences were used as input for SNV identification on the EBV genome in each sample. In detail, we used Samtools to obtain a list of potential SNVs. To discover reliable SNVs, we set a series of strict filters and statistical tests to reduce the frequency of false positives, like sequencing errors and misalignments. Using custom Perl scripts, SNVs that did not meet all of the thresholds (**Additional file 1: Table S7**) were removed from the mutational list. After identification of SNVs, we constructed phylogenetic trees of EBV genomes using genes with variable sequences. We used MAFFT for multiple-sequence alignment [15] of all 94 samples and 67 reference genomes (samples and references were excluded if the corresponding GenBank files lacked annotation for the genes used to construct the phylogenetic trees). Next, we utilized the maximum likelihood method implemented in RAxML based on the GTR+G+I model to generate the phylogenetic tree of each gene [16]. Gene-level selection analysis was performed using the BUSTED package on the Datamonkey web server [17, 18].

**DNA methylation analysis**

For whole genome bisulfite sequencing data, 150-bp paired-end reads were first trimmed of adaptors and 6-bp random primers. Next, we assessed sequencing quality using Trim_galore (Trim Galore 0.4.4) in single-end mode. The trimmed reads were aligned to a combined reference consisting of the hg19 human genome and the complete wild-type EBV reference genome (accession number NC_007605). Alignment and calculation of methylation levels (β values) were conducted using Bismark v0.19.0 in single-end, non-directional mode [19]. We also aligned the reads to the λ DNA for conversion efficiency assessment. For the human genome, by calculating the Euclidean distance of β values between any two samples from the same patient, we identified significant differences in the global methylation levels of normal tissues/LDs and EBVaGCs. To further characterize epigenetic changes during tumor development, DNA methylation profiles were processed with smoothing, after which differentially methylated regions (DMRs) were identified using the R package bsseq [20]. The criteria used to identify significant DMRs were as follows: a detection *P* value less than 0.05, and a difference of at least 0.3 in the mean β value of the region between 6 normal tissues/LDs and 6 EBVaGCs. The genomic context of all qualified DMRs was determined using the R package genomation [21]. Analysis of the functional enrichment of genes with promoter regions (transcription start site (TSS) ± 2000 bp) that overlapped with DMRs was performed using Metascape. For the EBV genome, due to the low coverage (**Additional file 1: Table S5**) of normal tissues and LDs, we merged the sequencing reads of EP13-N1 and EP13-LD. To investigate epigenetic changes during tumor development, we plotted the β values along the EBV genomes of EP13-C1, EP13-C5 and EP13-N1/LD, which were annotated according to the NC_007605 GenBank file. The two main repeat regions, IR1 (positions 12001–35355) and TR (positions 169636–171773) were not considered in the analysis due to sparse coverage.

**Virus quantity measurement**

To compare the absolute number of EBV genome copies in blood, saliva, and different histological samples, we randomly selected samples from each group and performed real-time qPCR using the AceQ U+ Probe Master Mix Kit (Vazyme) on a Bio-Rad CFX96 system (Bio-Rad). The number of EBV genome copies was measured using a region located within the conserved gene *BamHI*. The forward primer (5′-CCCAACACTCCACCACACC-3′), reverse primer (5′-TCTTAGGAGCTGTCCGAGGG-3′) and probe (5′-FAM- CACACACTACACACACCCACCCGTCTC-BHQ1-3′) were designed based on the complete wild-type EBV reference genome (accession number NC_007605). The B95-8 plasmid was used to generate the standard curve. By comparing the threshold cycle numbers of each sample with the standard curve, the absolute number of EBV genome copies per 10 ng extracted DNA was determined. The experiments used to generate the standard curve were repeated three times at each magnitude.

**Giemsa staining**

The tissue slides were deparaffinized and rehydrated. Naturally dried slides were incubated with Giemsa staining solution (Solarbio, Beijing, China, G1015) (diluted 10 times with distilled water) for 10-30 minutes, then rinsed with water. After that, the section were incubated with Phenol red solution for 10-30 minutes, then rinsed with water. Then, cover the slides with coverslips for further microscopic examination.

**Cell lines**

All human GC cell lines, including AGS, SNU719, HGC27, SNU16, and SNU5 were kindly provided by Professor Ruihua Xu (Sun Yat-sen University Cancer Center). AGS-EBV cells were derived from AGS cells with stable EBV infection. All cell lines are grown in DMEM (Gibco-BRL Life Technologies, Carlsbad, USA) supplemented with 10% fetal bovine serum (FBS; Gibco-BRL Life Technologies). All cell lines were maintained in a humidified incubator at 37°C with a 5% CO2.

**MTT assay**

Cells were treated with PI3K inhibitor LY294002 (Selleck, S1105) or Copanlisib (Selleck, S2802), Wnt pathway inhibitor ICG001 (Selleck, S2662) or Mebendazole (Selleck, S4610), or a combination of two agents for the indicated period of time. LY294002 is an inhibitor of PI3Kα/δ/β, whereas Copanlisib is an inhibitor of PI3Kα and PI3Kδ. ICG001 is an inhibitor of β-catenin, whereas Mebendazole is an inhibitor of TNIK and 5-Aza-2'-deoxycytidine (5-aza) is the DNA methylation inhibitor. To evaluate the effect of PI3K inhibitors or Wnt pathway inhibitors, cells were seeded in 96-well plates at a density of 1500 cells per well. After an overnight incubation, the cells were treated under the indicated conditions. To evaluate the effect of 5-aza, seeded cells were treated with 5-aza at a final concentration of 2.5 and 5 μM, respectively, with Aza-containing medium being changed every 24 hours. At the end of the treatment, 20 µL of 3-(4,5-dimethylthiazol-2-yl)-2,5 -diphenyltetrazolium bromide was added to each well, after which the plates were incubated at 37 °C for 4 hours. Next, the supernatants were aspirated carefully, after which formazan crystals were dissolved in DMSO. Finally, the absorbance was measured at 490 nm. The concentration of each PI3K inhibitor, Wnt pathway inhibitor, and combination that produced 50% growth inhibition (IC50) was calculated using a relative survival curve 48 hours after treatment. Drug combination experiment was performed by setting serial concentrations of one drug as above with a fixed concentration (IC25 or IC50) of another drug. Furthermore, the value of combination index (CI) calculated by CalcuSyn software was used to determine synergism between drug combinations [22-24].

**Colony formation assay**

Cells (2,000 cells per well) were seeded in a 6-well plate. After an overnight incubation, the cells were treated under the indicated conditions or treatments and cultured for 14 days in a humidified 5% CO_2_ incubator at 37 °C. To evaluate the effect of 5-aza, seeded cells were treated with 5-aza at a final concentration of 2.5 and 5 μM, respectively, with Aza-containing medium being changed every 24 hours. Colonies were fixed in methanol for 10 min and stained with 0.5% crystal violet for 15 min. All visible colonies were quantified. At least three independent experiments were performed for each assay.

**RNA extraction and semi-quantitative RT-PCR**

EBV positive gastric cancer cell lines, SNU719 and AGS-EBV, were seeded at 6-well plate with a density of 1×e05 cells/ml and incubated overnight. Medium was then replaced with fresh medium containing 5-Aza at a final concentration of 2.5, 5 and 10 μM, respectively. 5-aza treatment lasted for 72 hours, with changing of Aza-containing medium every 24 hours. For combined treatment of 5-aza with TSA, cells were treated with TSA (100 ng/ml in DMSO) for additional 24 hours after Aza treatment. Total RNA was extracted using TRI Reagent and then performed reverse transcription. cDNA products were amplified with Taq polymerase (Invitrogen) for 40 cycles, with ACTB (32 cycles) as the control. The sequences of primer sets were listed below:

| Primers | Sequence (5’ - 3’) |
| --- | --- |
| RASA3-F | TCCAGAGCGTGAAGATCAAG |
| RASA3-R | TTGTGGTACTTCTGCAAGTC |
| RASA4-F | TGTAGCCATCACTGCCTCTCCA |
| RASA4-R | CCCGTTCTCCCTCCCATTCTCT |
| RASSF1A-F | GCGTCGTGCGCAAAGG |
| RASSF1A-R | TGCTGTTGATCTGGGCATTG |
| RASAL3-F | TCTGGGGCGTCGTGCGCAAA |
| RASAL3-R | GAACCTTGATGAAGCCTGTG |
| ACTB-F | CATGTACGTTGCTATCCAGGC |
| ACTB-R | CTCCTTAATGTCACGCACGAT |

**Western blotting**

Total protein was extracted from AGS-EBV cells treated with different treatments (Copanlisib 0.05μM, Mebendazole 0.15μM) or vehicle for 24 hoursusing SDA lysis buffer. Protein lysate were separated via 9% SDS-PAGE. After electrophoresis, the proteins were transferred to the PVDF membrane (Millipore, Burlington, MA, USA). The membranes were subsequently blocked in 5% milk for 1 hr. Then the membrane was incubated with anti-phospho-Akt antibody (Try483)(GeneTex,GTX50128,China) and anti-GAPDH antibody (6004-1,Proteintech, Rosemont, IL, USA) separately at 4℃ overnight. The species-matched secondary antibodies were then hybridized with the membranes at room temperature for 45 minutes. Protein was visualized by enhanced chemiluminescence (Thermo, Waltham, MA, USA).

**Luciferase assay**

AGS-EBV cells were seeded at 1X10^4^ cells per well in 48 well plates for 16 hours and then transfected with TK-Renilla plasmid (10ng) and TOPflash or FOPflash reporter plasmids (250ng) using PEI (YEASEN, 40816ES03, China). After 4-6hr, fresh medium was changed and cells were treated with LiCl (30mM) and different treatments (vehicle, Copanlisib 0.03μM, Mebendazole 0.15μM or the combination). The luciferase activities were detected using the Dual-Glo Luciferase Assay System (Promega, #E2940, Madison, WI, USA) at 24 hours post-transfection. The relative luciferase activities were normalized against the values of the Renilla luciferase signal.

***In vivo* assay**

The study was performed on four- to five-week-old female immune-deficient mice (BALB/c nude mice) which were purchased from Model Animal Research Center of Nanjing University according to ethics approval number L102012017003G. BALB/c nude mice were housed in isolated cages, under specific pathogen-free (SPF) conditions with a 12 h light/dark cycle and maintained at 22℃± 1 at the animal facility of Cancer Prevention Center, Sun Yat-sen University. Mice were inoculated subcutaneously with 1 mm^3^ SNU719 xenograft tumor fragments. When the tumor reached the predetermined size, mice were randomized to the control or experimental group. Copanlisib was dosed intraperitoneal injection at 6 mg/kg with a Q2D schedule. Mebendazole was dosed at 20 mg/kg orally with a once daily schedule. Body weights and tumor volumes were measured every day. Tumors were removed and weighed under isoflourane anesthesia at completion of the study. The following formula was used to determine tumor volumes: tumor volume =L×W^2^/2, in which L is the length and W the width.

**Quantification and statistical analysis**

For the sequencing data, statistical analyses and graphics production were performed using R v3.5.0 (Foundation for Statistical Computing). For the experimental data, statistical analyses and graphics production were performed using GraphPad Prism 7 (GraphPad Software). All hypothesis tests were 2-sided. Statistical tests are specified in the Results section and the Figure Legends.

**
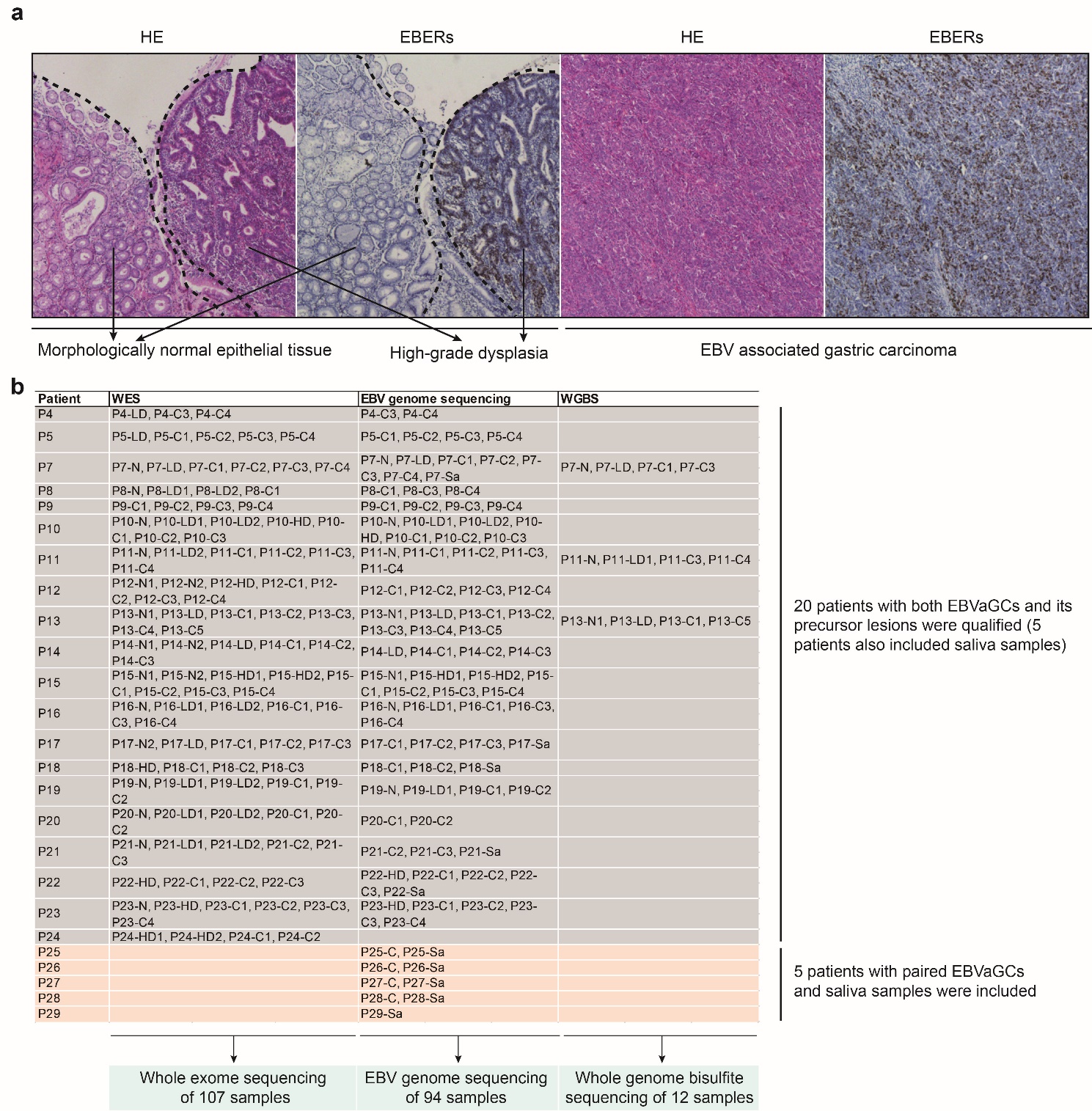
**

**Fig. S1**

**Overview of the study cohort. a**) Diagram showing the hematoxylin- and eosin- (HE) stained and *in situ* hybridization of Epstein-Barr Encoded RNA sections of morphologically normal epithelial tissue (N) and high-grade dysplasia (HD) (left), and EBV-associated gastric carcinoma (EBVaGC) (right) from a representative patient P15. Arrow indicate the neighboring areas of normal epithelial tissues and HD. The magnification is 4 fold. **b**) Details of samples from each patients and the analyses in our work.


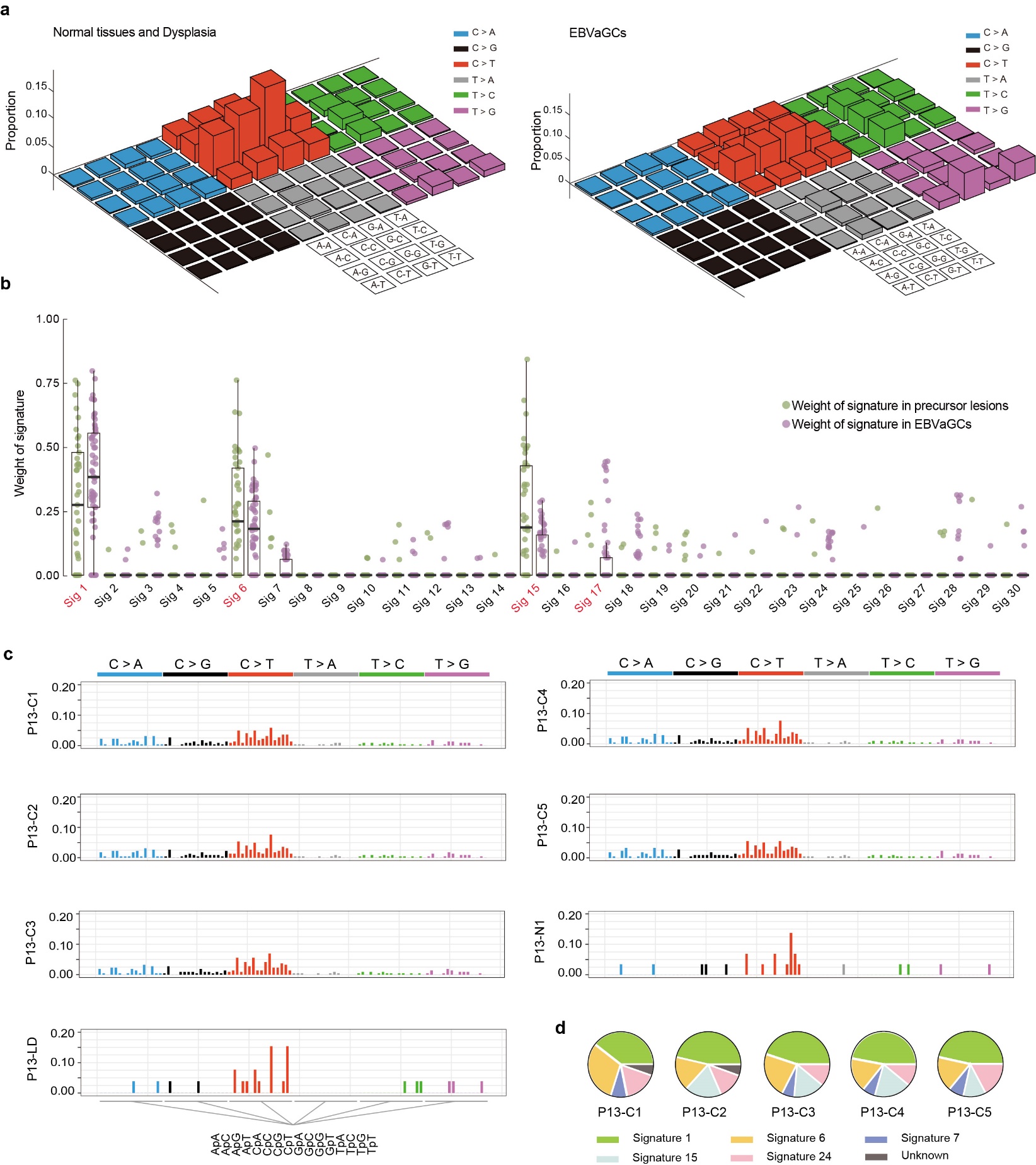


**Fig. S2**

**Mutational signatures in EBVaGCs and their precursor lesions. a**) ‘Lego’ plots displaying the frequency of base substitutions within specific trinucleotide mutational contexts in all precursor lesion samples (left, n=45) and EBVaGCs (right, n=62). **b**) The weight of decomposed COSMIC signatures in each of precursor lesions (green dots) and EBVaGCs (purple dots). The mean value is indicated. **c**) Bar plots of the frequencies of base substitutions within specific trinucleotide mutational contexts in each sample of patient P13. **d)** Pie charts showing the fractions of COSMIC signatures extracted by deconstructSigs in 5 EBVaGCs of P13.

**
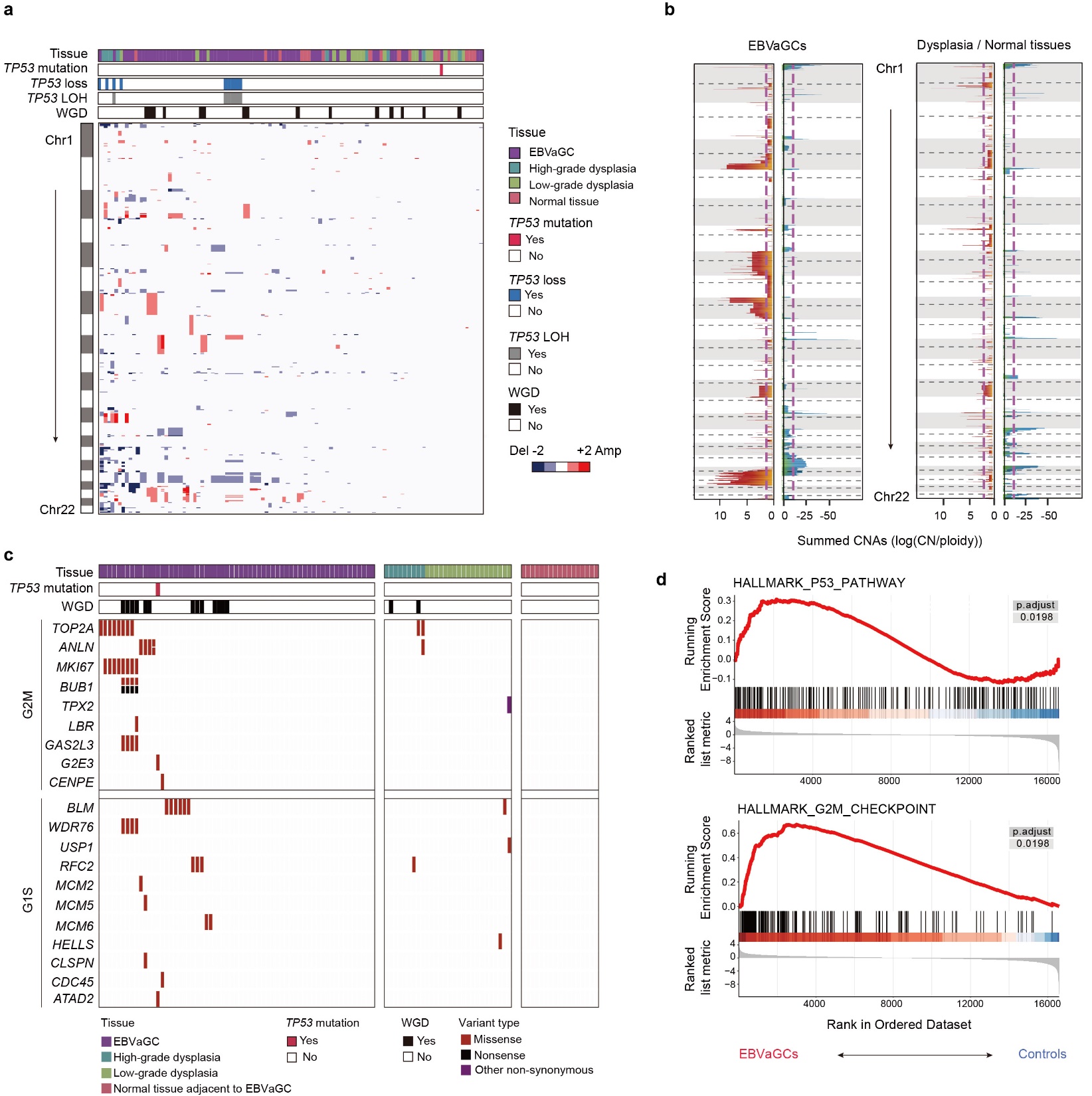
**

**Fig. S3**

**Copy number alterations upon the view of the whole genome. a**) Heatmap of copy number alterations (CNAs) across the genomes in normal tissues (n=17), LDs (n=19), HDs (n=9) and EBVaGCs (n=62). The histological types, *TP53* mutations, *TP53* loss, *TP53* loss of heterozygosity (LOH) and whole genome doubling (WGD) status of each sample are indicated by different color bars on the top. Chromosomes are shown on the left. **b**) Stacked mountain plots displaying the summed CNAs for 62 EBVaGCs (left) and 45 precursor lesions (right). The copy number gains and losses are denoted by warm-color and cool-color peaks, respectively. The dash purple line indicates the observed value in our data corresponding to an empirical *q* value of 0.05 (*P* values obtained from permutation test and corrected by multiple testing using Benjamini-Hochberg method). **c**) Heatmap of the recurrently mutated genes in G1S and G2M pathways in EBVaGCs (right) and precursor lesions (left). The histological types, *TP53* mutations and WGD status of each sample are indicated by different color bars on the top. **d**) GSEA showing genes involved in the P53 and G2M pathway are significantly up-regulated in EBVaGCs (n=12) compared to normal controls (n=12). The transcriptome data was acquired from GEO database (GSE51575).


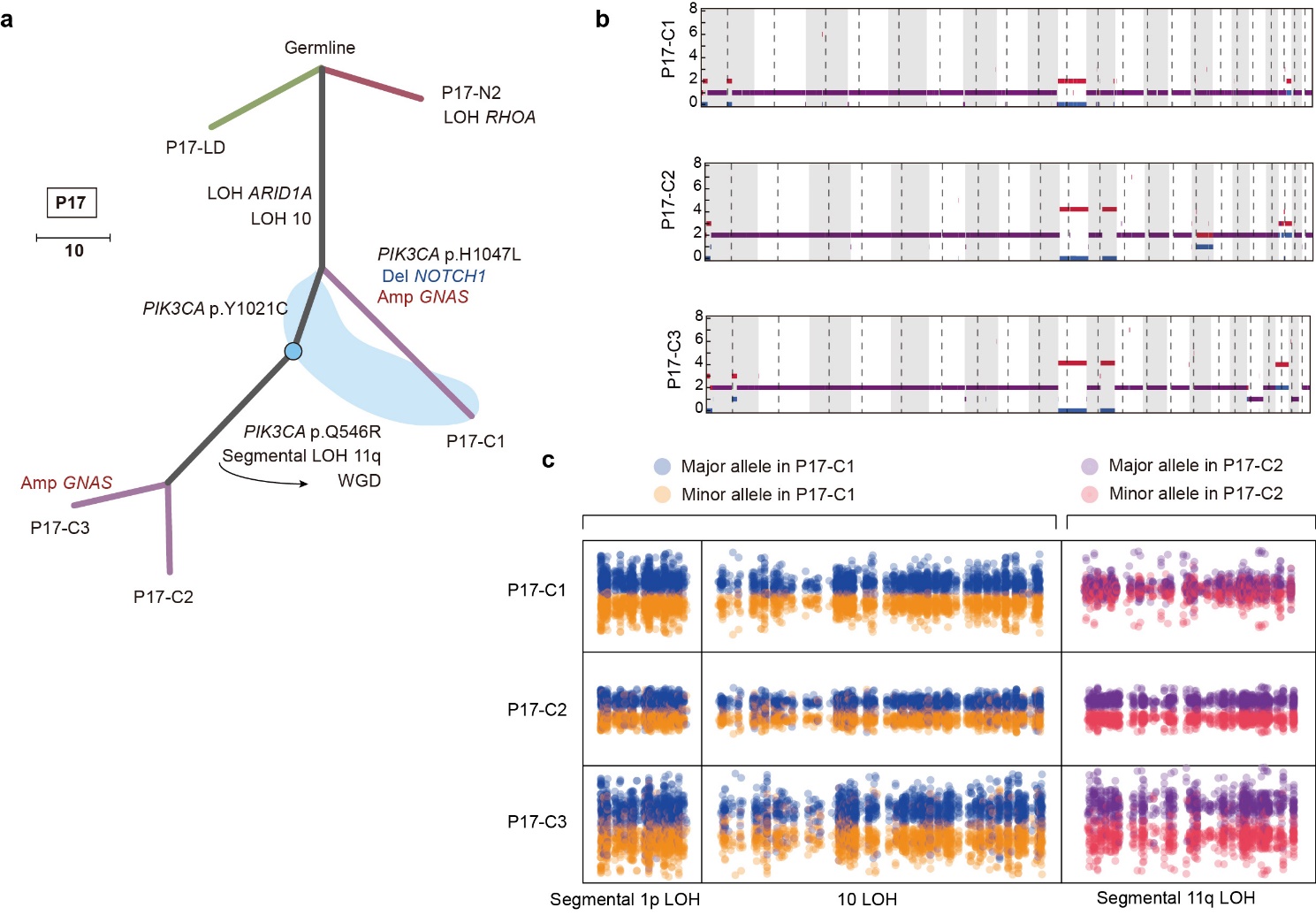


**Fig. S4**

**Homologous tracking of loss of heterozygosity in patient P17. a**) Phylogenetic tree for patient P17. The length of each line is proportional to the number of mutations and copy number alterations (CNAs). Grey lines represent the clonal mutations shared by multiple samples. A subclone in one EBVaGC (P17-C1) shared as full clones in other two EBVaGCs (P17-C2 and P17-C3) is indicated. Shaded area contains mutations present in the shared subclone on corresponding branches. **b**) Allelic copy numbers of 3 EBVaGCs from P17. The red line represents the major allele and the blue line represents the minor allele. **c**) Dot plots displaying the homologous tracking results of loss of heterozygosity (LOH) events in 3 EBVaGCs from P17. Segmental chr1p LOH, chr10 LOH and segmental chr11q LOH are determined as the results shown above. Dot colors indicate the established tags of major and minor alleles as reference or mutant alleles. This diagram exemplifies the occurrence of LOH events in P17 resulted from the same allele loss event, and we did not observe the convergent evolution of LOH events in other patients (data not shown).

**
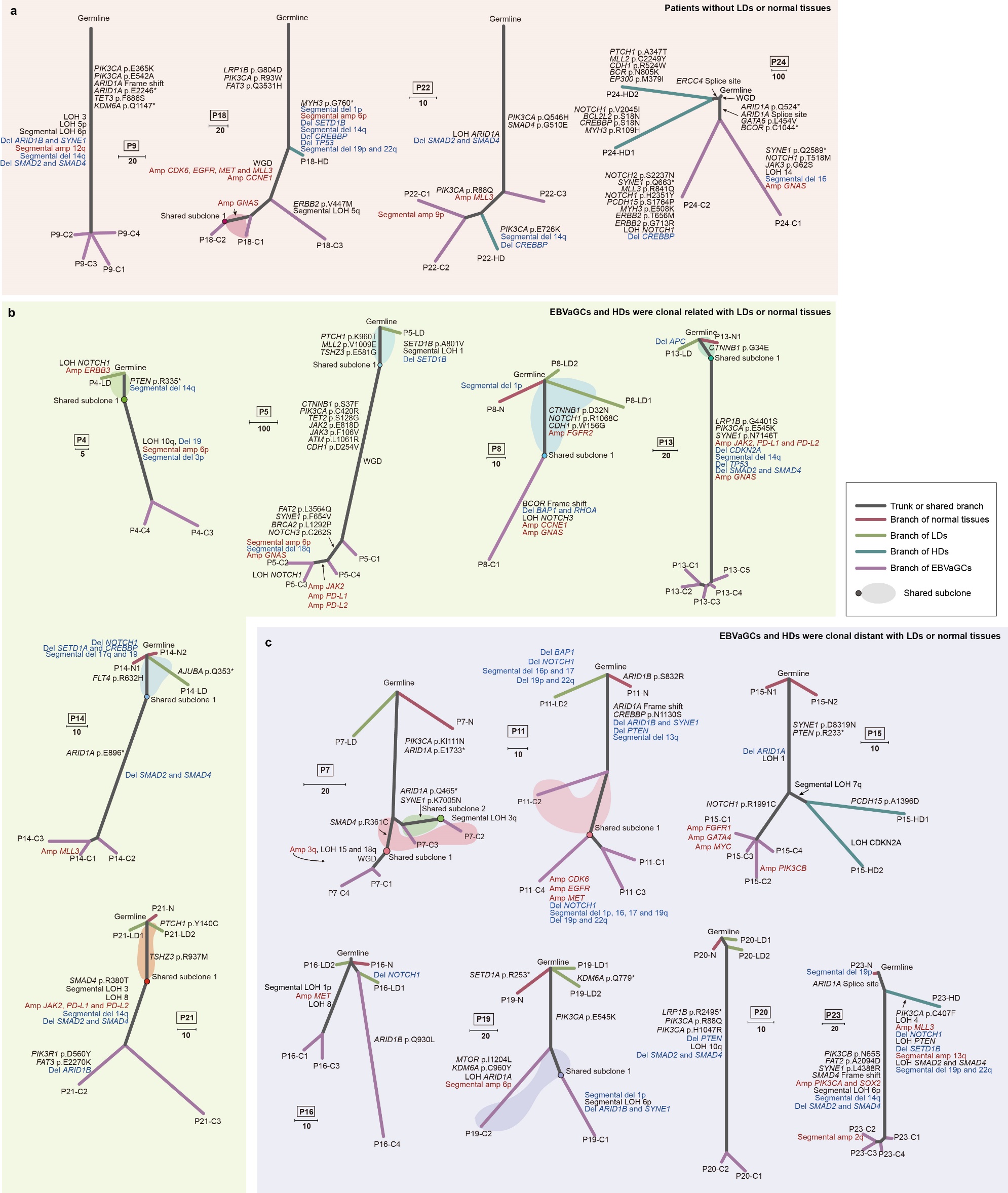
**

**Fig. S5**

**Phylogenetic trees for all patients. a**) Phylogenetic trees for patients without LDs or normal tissues. The length of each line is proportional to the number of mutations and copy number alterations (CNAs). Grey lines represent the clonal mutations shared by multiple samples. **b**) Phylogenetic trees for patients with clonal related EBVaGCs and precursor lesions. Subclones in normal tissues or LDs that shared as common ancestors in EBVaGCs from the same patient are indicated. Shaded area contains mutations present in the shared subclone on corresponding branches. **c**) Phylogenetic trees for patients with clonal distant EBVaGCs and precursor lesions. One subclone shared by two EBVaGCs in patient P19 is indicated.

**
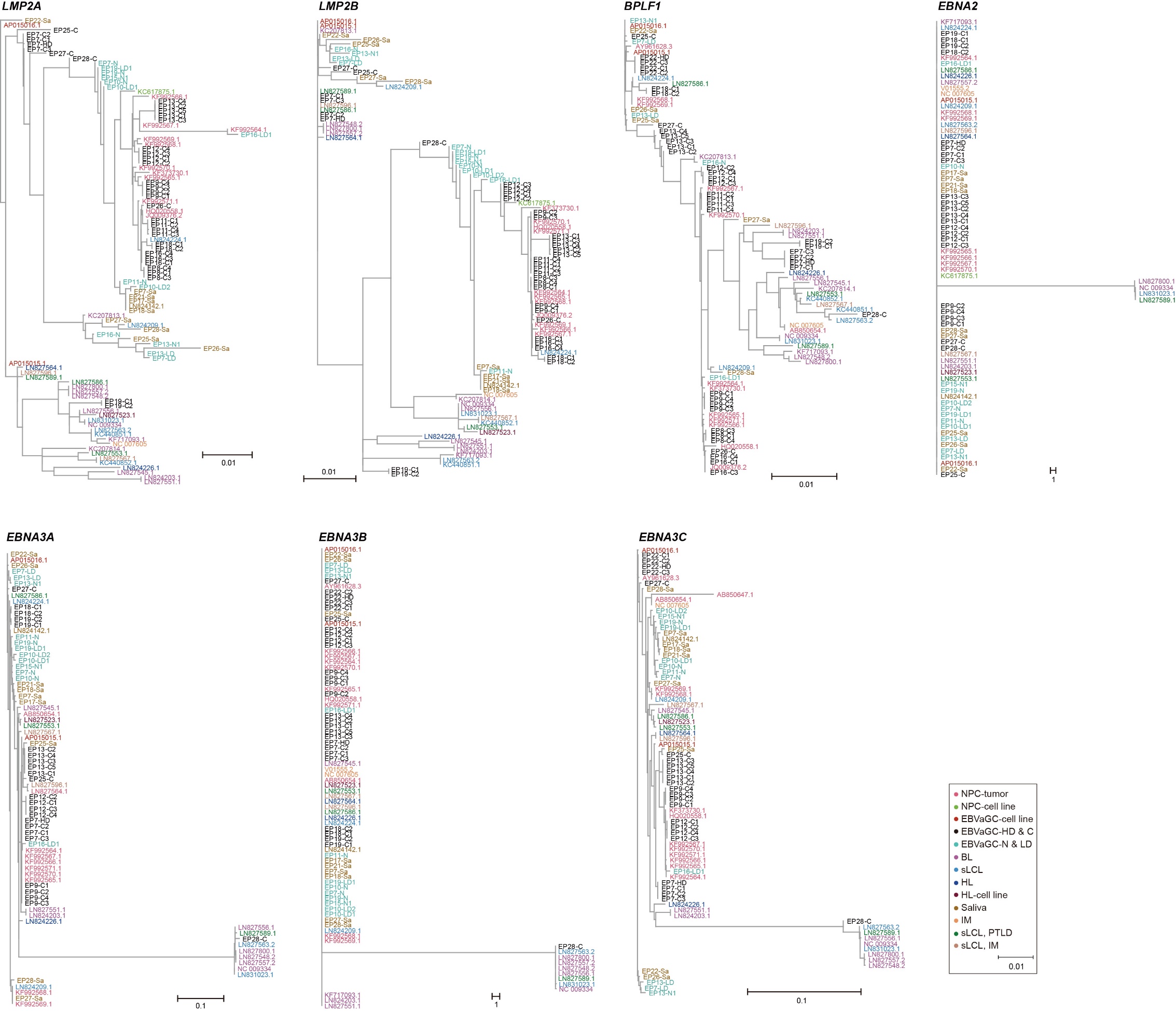
**

**Fig. S6**

**Phylogenetic trees of genes with variable sequences from EBV genomes.** The name of each EBV strain is shown in different colors according to the type of the sample from which the EBV strain is identified. The scale bar represents the ­number of nucleotide substitution per site. IM, infectious mononucleosis; sLCL, spontaneous lymphoblastoid cell line; EBVaGC, EBV-associated gastric carcinoma; NPC, nasopharyngeal carcinoma; BL, Burkitt’s lymphoma; HL, Hodgkin’s lymphoma; PTLD, posttransplant lymphoproliferative disease.

**
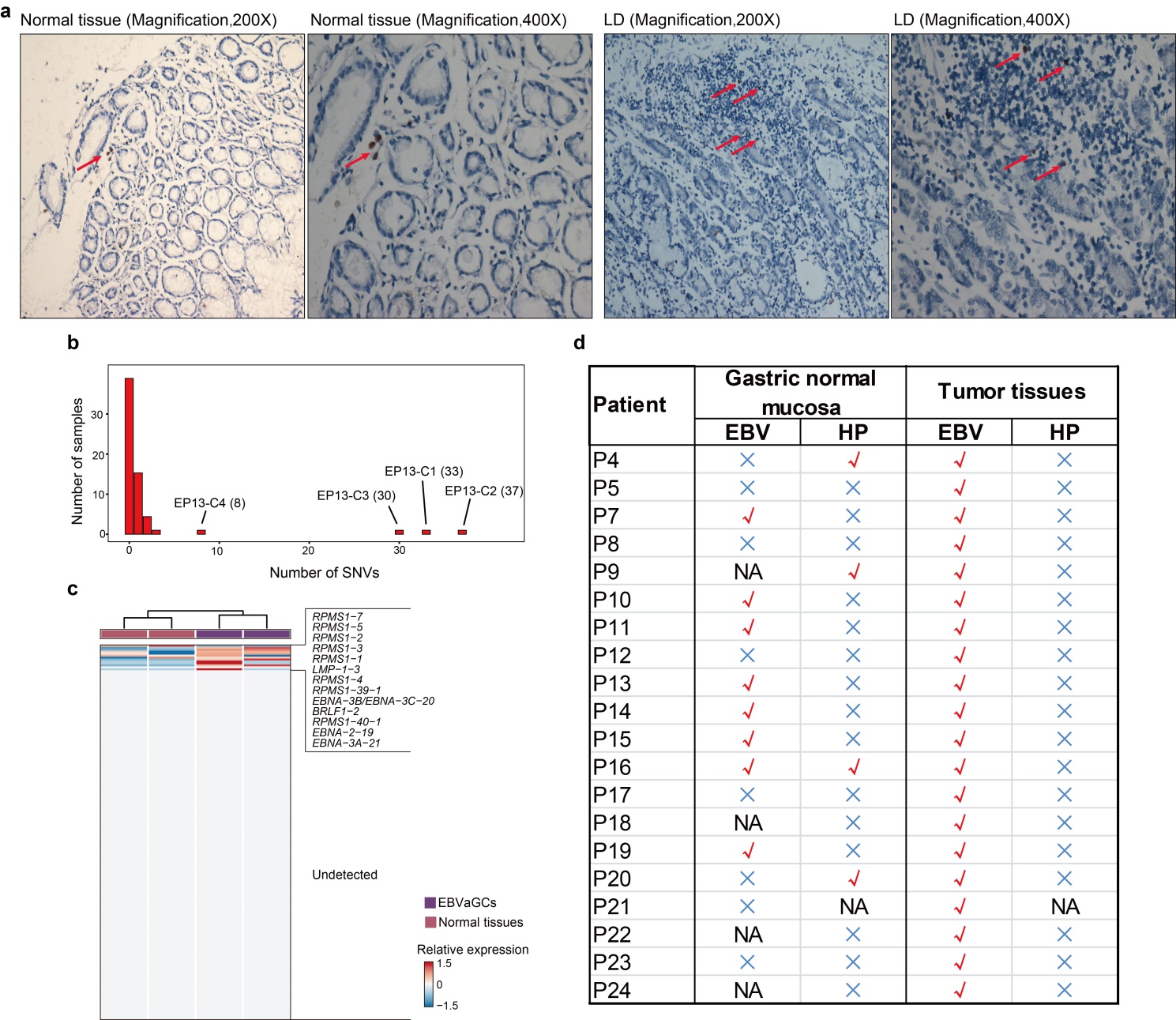
**

**Fig. S7**

**EBV infection in a few B lymphocytes in normal tissues and LDs. a**) *In situ* hybridization of Epstein-Barr Encoded RNA sections of morphologically normal epithelial tissue (N) and low-grade dysplasia (LD) from a representative patient P7. The red arrows indicate the rare positive signals of EBV infected B lymphocytes. **b**) Histogram showing the number of private SNVs on EBV genomes within EBVaGCs/HDs of each patient. The few number of private SNVs in each sample of most patients suggests the limited evolution of virus within the lifetime of tumor development. **c**) Heatmap displaying the expression of EBV genes in EBVaGCs and normal tissues. Relative expression was defined as the gene-wise (row) z-score of normalized read counts across four samples. Names of detected genes (read count > 0) are listed. **d**) Chart showing the presence of EBV assessed by EBV genome capture, and *Helicobacter pylori* (HP) assessed by Giemsa staining.

**
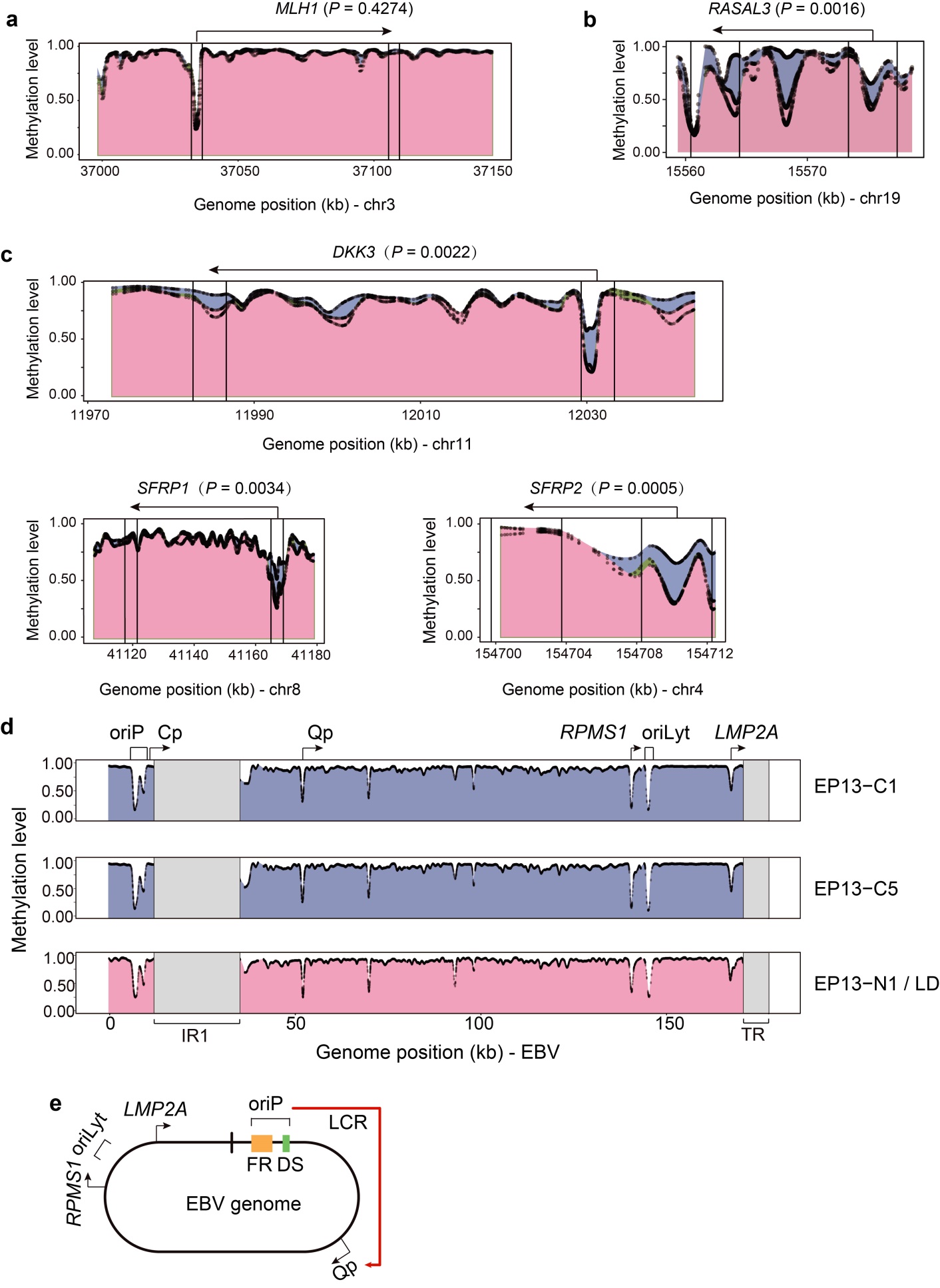
**

**Fig. S8**

**Epigenetic changes in promotor regions from precursor lesions to EBVaGCs. a**) The promoter region of *MLH1* showing consititutively unmethylated in EBVaGCs in comparison with that in normal tissues and LDs. Black dots represent each CpG site. Different colors of areas indicate the histological types of samples (pink, normal tissues; green, LDs; blue, EBVaGCs). The transcription strand is indicated by the arrow orientation (left, reverse strand; right, forward strand). The statistical significance is shown (Student’s *t* test). **b**) *RASAL3*, which encodes one Ras GTPase-activaing protein (RasGAP), showing hypermethylation in EBVaGCs in comparison with that in normal tissues and LDs. **c**) Three genes from the Wnt pathway showing hypermethylated promotors in EBVaGCs compared with normal tissues and LDs. The silencing of these genes by methylation are also found in nasopharyngeal carcinomas. **d)** The epigenomic profiling of EBV genomes in EBVaGCs and precursor lesions of patient P13. Two main repeat regions (IR1 and TR) were masked out due to sparse coverage. **e)** Schematic of the episome of EBV labelled with unmethylated promotors in EBVaGCs and their precursor lesions. FR, family of repeats; DS, dyad symmetry; LCR, locus control region.

**
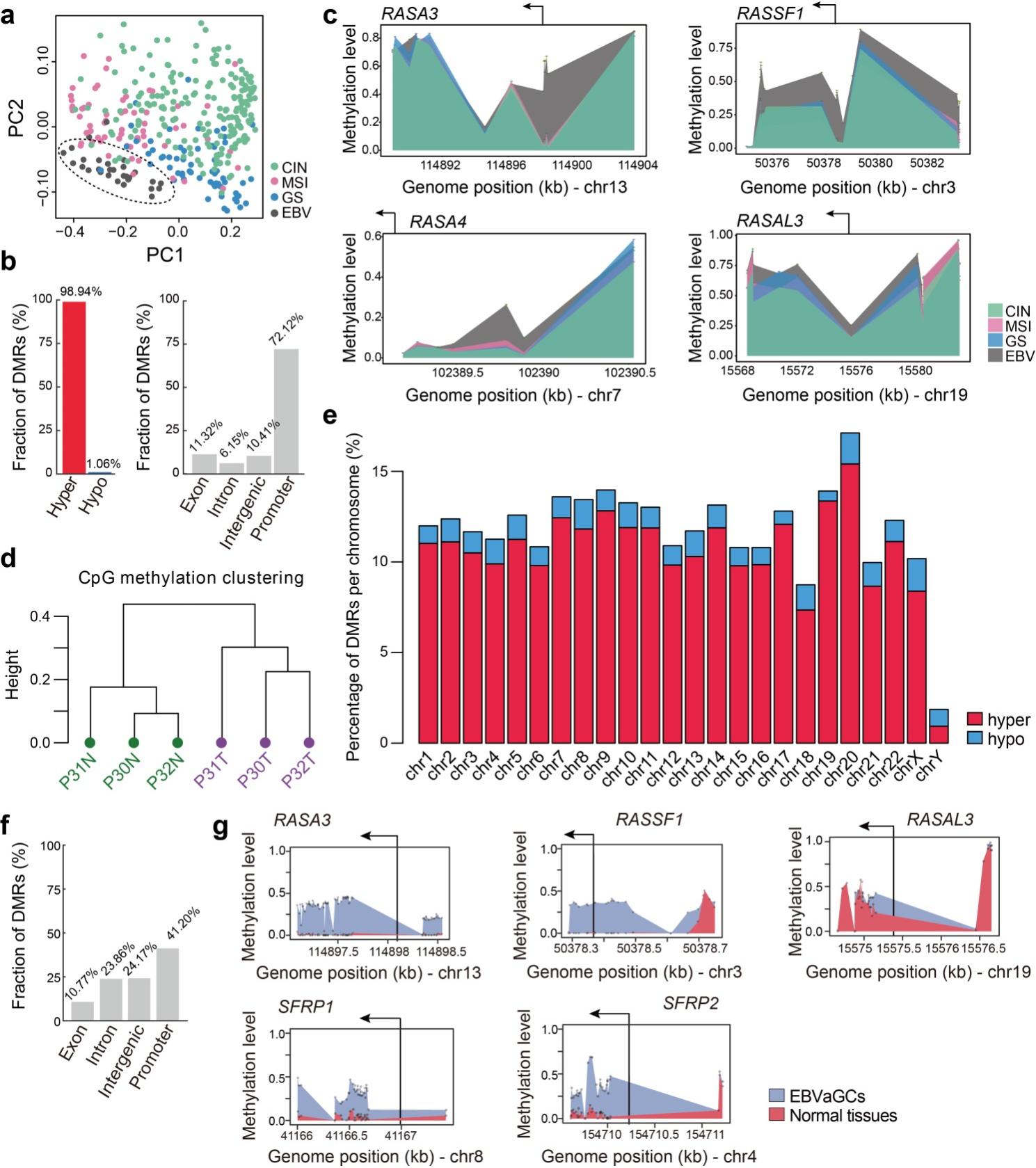
**

**Fig. S9**

**Validation of hypermethylated profiling of EBVaGC. a**) Principal-component analysis (PCA) plot of DNA methylation profiles in 4 different molecular subtypes of gastric cancer from TCGA cohort. **b**) Bar plots of the fractions of hyper- and hypo-methylated DMRs of EBVaGC in comparison with other gastric cancer types (left), and fractions of DMRs overlapping with different genomic elements (right). **c**) Genes encoding Ras GTPase-activaing proteins (RasGAP) showing hypermethylated promotors in EBVaGCs in comparison with other gastric cancer types from TCGA cohort. CIN, chromosomal instability; MSI, microsatellite instability; GS, genomically stable. **d**) Clustering of normal tissues and EBVaGCs from 3 patients. All samples were fresh frozen samples and processed with RRBS. The clustering analysis was based on pearson correlation of CpG methylation level. Green dots, normal tissues; purple dots, EBVaGCs. **e**) Bar plots showing the frequency of hyper- or hypo-methylated CpG sites in all detected CpG sites on each chromosome. **f**) Bar plots of the fractions of hypermethylated regions from RRBS data overlapping with different genomic elements. **g**) RRBS data showing that genes encoding Ras GTPase-activating proteins (RasGAP) and Wnt antagonists showing hypermethylated promotors in EBVaGCs in comparison with normal tissues.

**
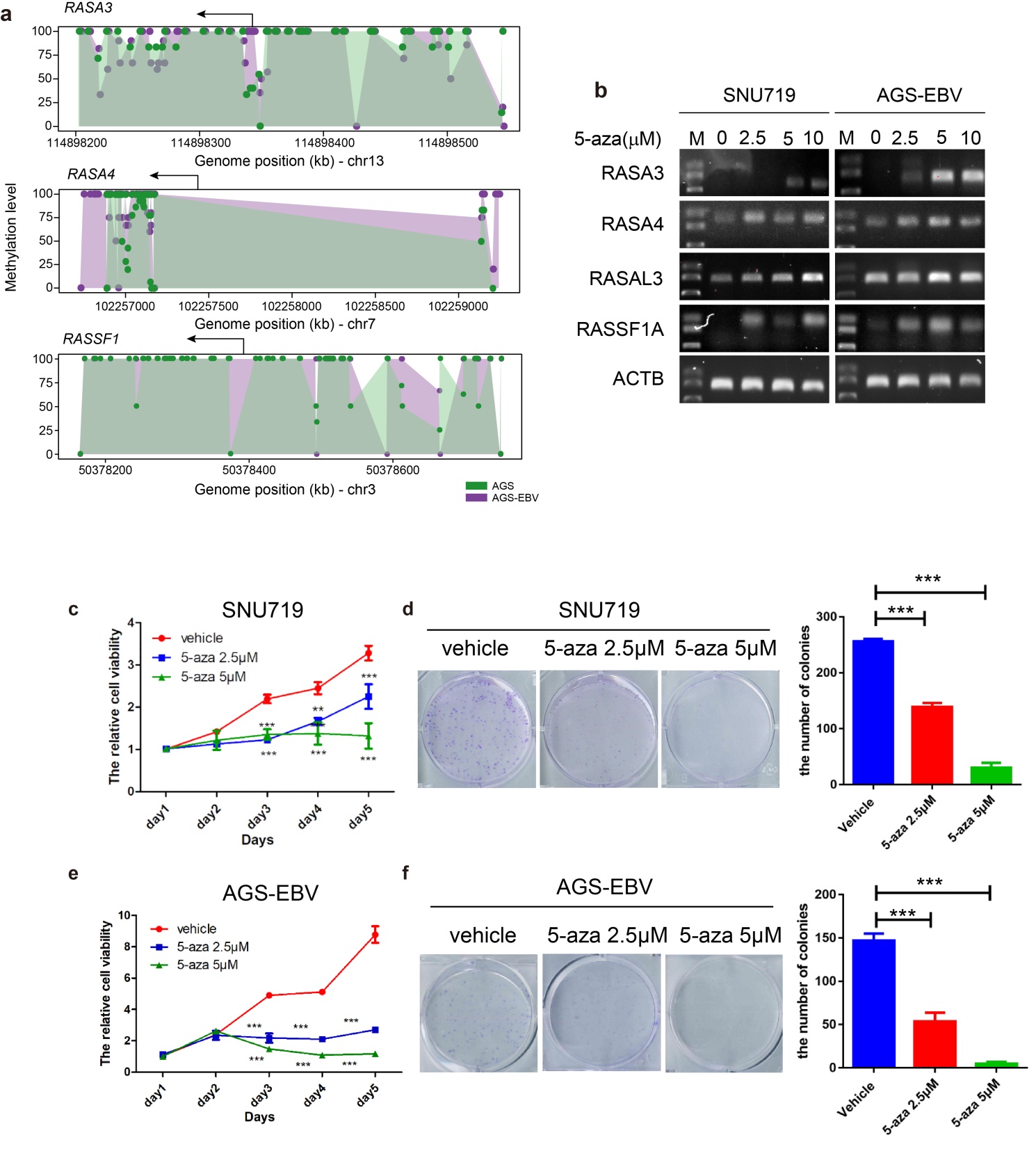
**

**Fig. S10**

**Demethylation treatment reduce the cell proliferation and clonogenicity abilities in EBV-positive GC cells a**) Methylation level of RasGAP family genes in AGS and AGS-EBV cell lines. Different colors of areas and dots indicate the AGS cell line before or after EBV infection. Each dot represents a CpG site. The transcriptional starting site (TSS) and transcription strand is indicated by the arrow orientation (left, reverse strand; right, forward strand). **b**) SNU719 and AGS-EBV cells were treated with different doses of 5-Aza-2'-deoxycytidine (5-aza, DNA methylation inhibitor) for 72 hours and then treated with TSA for 24 hours. RASA3/4, RASAL3, and RASSF1A and β-actin were amplified by PCR. β-actin was served as a negative control. **c-d**) SNU719 cells were treated with vehicle, 2.5μM 5-aza, or 5μM 5-aza (5-aza) for 5 days (c) or 8 days (d). Cell viability was measured by MTT assay in SNU719 cells (c). The representative pictures and quantitative analysis of colony formation of SNU719 cells (d). **e-f**). AGS-EBV cells were treated with vehicle, 2.5μM 5-aza, or 5μM 5-aza (5-aza) for 5 days (e) or 8 days (f). Cell viability was measured by MTT assay in AGS-EBV cells (e). The representative pictures and quantitative analysis of colony formation of AGS-EBV cells (f).

**
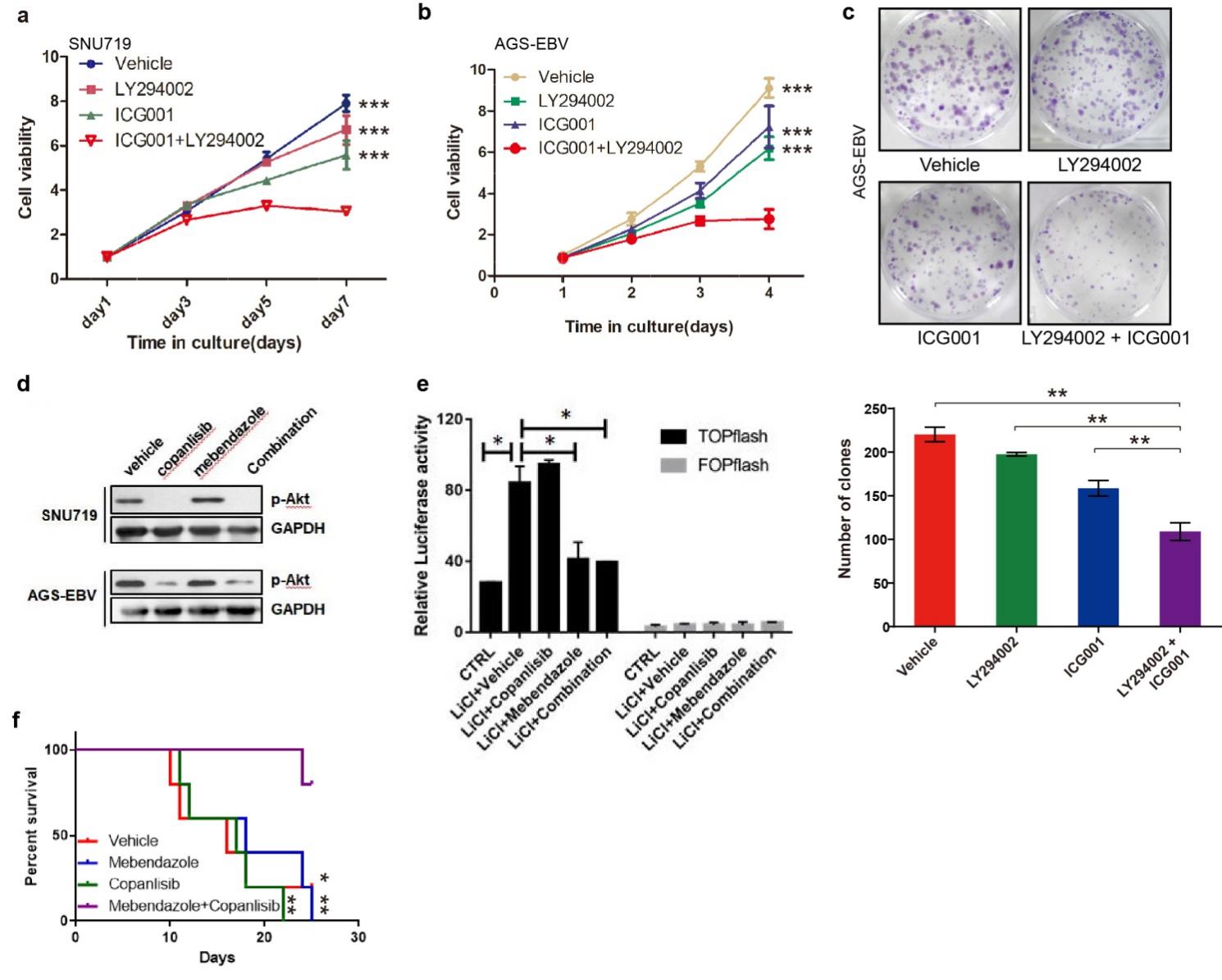
**

**Fig. S11**

**Validations of synergistic function of the PI3K-Akt and Wnt pathways on EBV-positive tumor cells. a**) The growth curve of SNU719 cells treated with either 5 μM LY294002 or 1.5 μM ICG001, or the combination. (n=3). **b**) The growth curve of AGS-EBV cells treated with either 12.8 μM LY294002 or 0.8 μM ICG001, or the combination. (n=4). **c**) Comparison of colony formation of AGS-EBV cells with different treatments or vehicle. (n=3). All statistics were conducted using Student’s *t* test, **P*<0.05, ***P*<0.01, ****P*<0.001. Data are shown as mean ± SD. **d**) Western Blotting of p-Akt in SNU719 and AGS-EBV cells treated with different treatments as indicated for 24 hours. **e)** TOPflash/FOPflash reporter analysis in AGS-EBV treated with LiCl (30 μM) and different treatments as indicated for 24 hours. The statistics were conducted using Student’s t test, *P<0.05. Data are shown as mean ± SD (n=3). **f**) The survival curve of SNU719 xenografts mice with different treatments (Copanlisib 6mg/kg, Mebendazole 20mg/kg) or vehicle (5 mice per group).

**
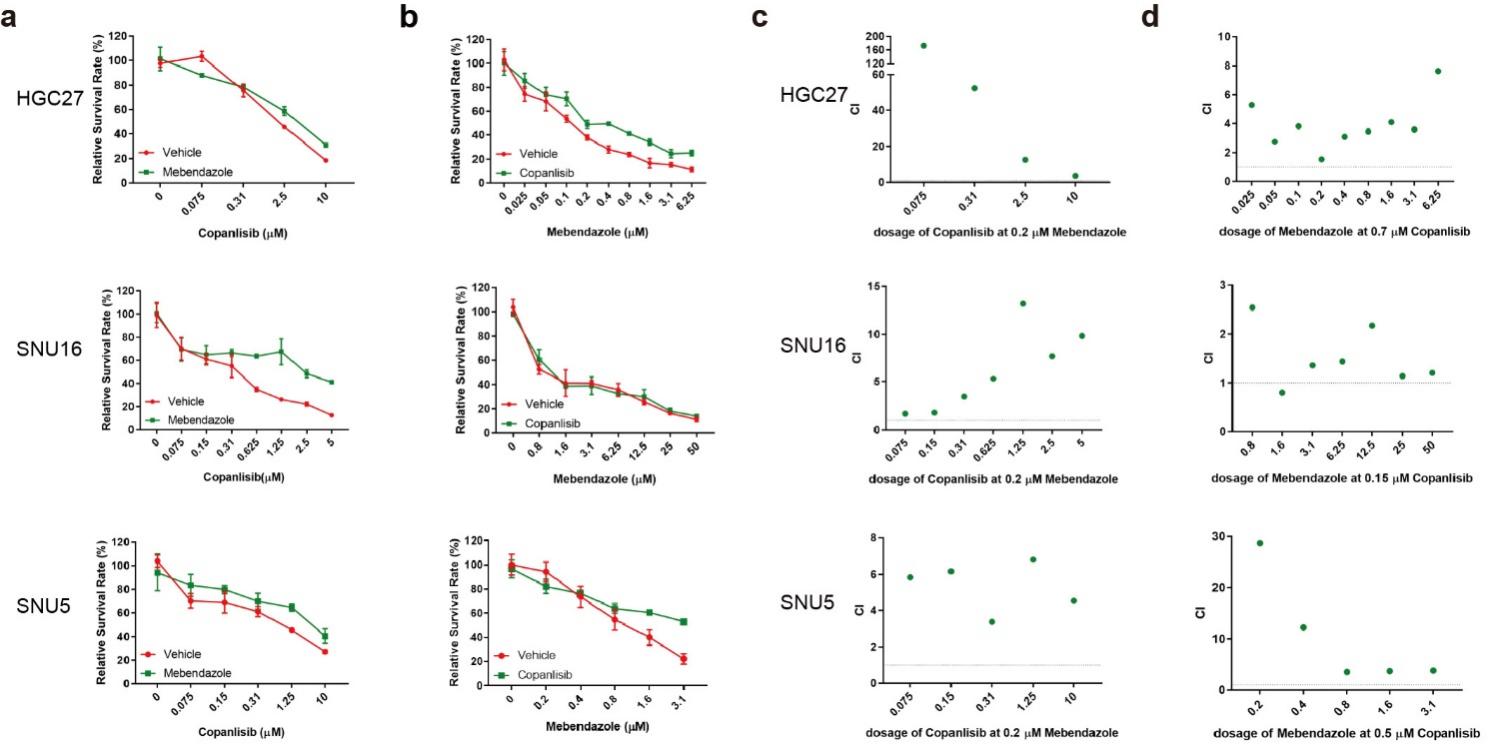
**

**Fig. S12**

**The effects of the combination of Copanlisib and Mebendazole in gastric cancer cell lines**

**a**) The growth curve of HGC27, SNU16 and SNU5 cells treated with the indicated doses of Copanlisib at the presence of Mebendazole or Vehicle. (n=3). **b**) The CI of HGC27, SNU16 and SNU5 cells treated with the indicated doses of Copanlisib at the presence of Mebendazole. (n=3). **c**) The growth curve of HGC27, SNU16 and SNU5 cells treated with the indicated doses of Mebendazole at the presence of Copanlisib or Vehicle. (n=3). **d**) The CI of HGC27, SNU16 and SNU5 cells treated with the indicated doses of Mebendazole at the presence of Copanlisib. Data are shown as mean ± SD. (n=3).


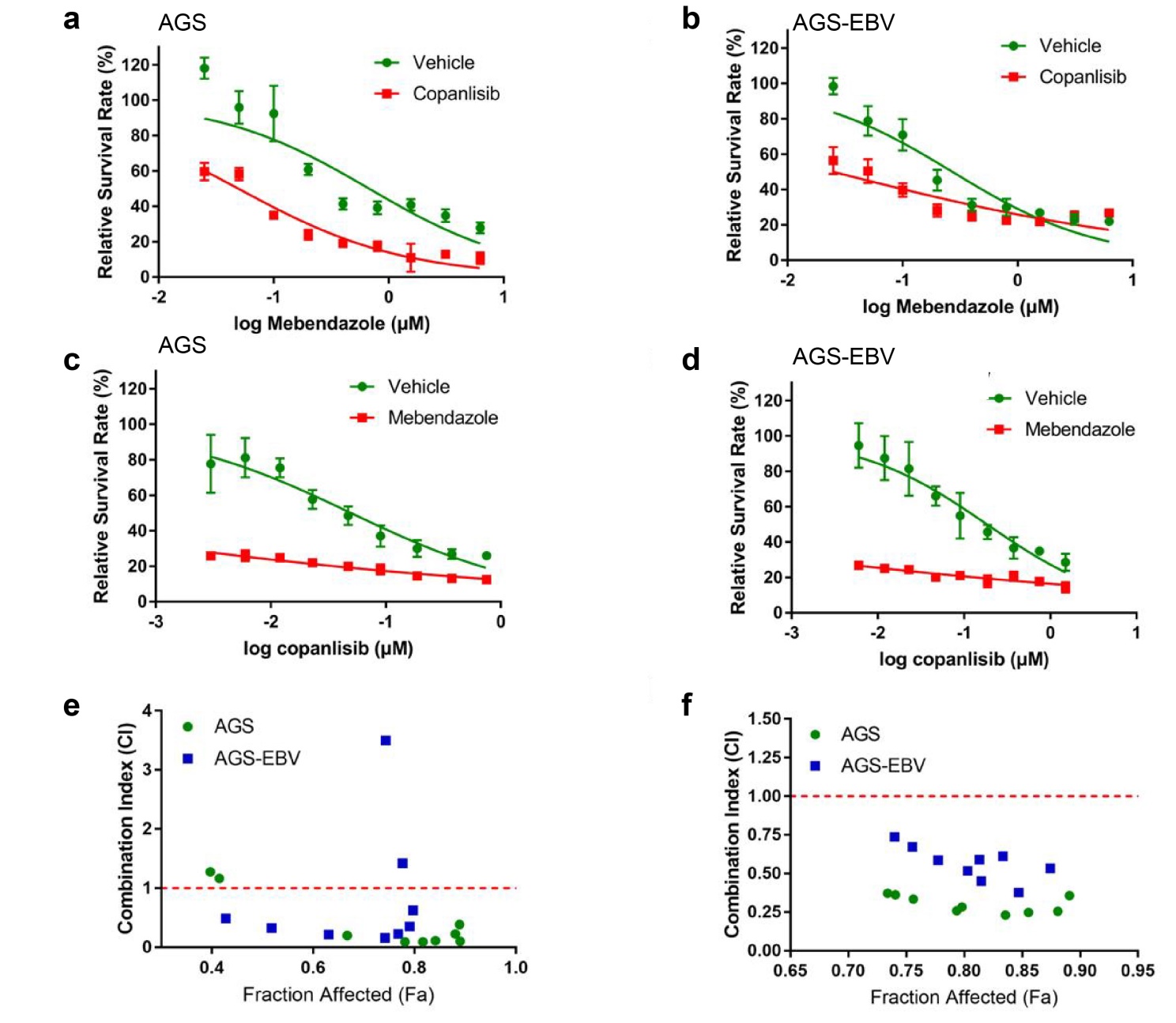


**Fig. S13**

**The synergistic effects of Copanlisib and Mebendazole in AGS and AGS-EBV cells.** **a-b**) The growth curve of AGS cells or AGS-EBV cells treated with the indicated doses of Wnt pathway inhibitor (Mebendazole) at the presence of PI3K inhibitor (0.03 μM Copanlisib) or Vehicle. (n=3). **c-d**) The growth curve of AGS cells or AGS-EBV cells treated with the indicated doses of PI3K inhibitor (Copanlisib) at the presence of Wnt pathway inhibitor (0.65 μM Mebendazole) or Vehicle. (n=3). **e**) The CI of AGS cells (green dots) or AGS-EBV cells (blue squares) treated with the indicated doses of Mebendazole at the presence of 0.03 μM Copanlisib. **f**) The CI of AGS cells (green dots) or AGS-EBV cells (blue squares) treated with the indicated doses of Copanlisib at the presence of 1.3 μM Mebendazole.

**Table S1. Clinical information of patients**

| **Patient** | **Gender** | **Age** | **Tumor location^a^** | **Stage** | **Tumor size（mm）** | **Invasion^b^** | **Differetiation^c^** | **Lauren type^d^** | **EBV** | **HER2** | **CA72 (0-5.30 U/ml)** | **CEA(0.00-5.00 ng/ml)** | **CA19-9 (0.00-35.00 U/ml)** |
| --- | --- | --- | --- | --- | --- | --- | --- | --- | --- | --- | --- | --- | --- |
| P4 | M | 54 | 3 | NA | 20*15*10 | 3 | 3 | 1 | + | 1+ | 20.75 | 2.18 | 1.98 |
| P5 | M | 55 | 3 | pT4aN3bM0/IIIC | 60*45*12 | 5 | 3 | 1 | + | NA | 22.22 | 0.667 | 14.85 |
| P7 | M | 69 | 2/3 | pT3N3M0/IIIB | 40*30*10 | 4 | 1-2 | 1 | + | 1+ | 1.67 | NA | NA |
| P8 | M | 51 | 2 | pT4N3bM0/IIIC | 40*40*8 | 5 | 3 | 3 | + | 2+ | 1.07 | 3.22 | 6.21 |
| P9 | M | 72 | 3 | pTxNxM1/IV | 105*30*20 | 4 | 3 | 2 | + | 1+ | 0.671 | 2.7 | 7.01 |
| P10 | M | 61 | 3 | NA | 35*25*15 | 1-4 | 2-3 | 3 | + | 1+ | NA | NA | NA |
| P11 | M | 67 | 3 | pT4N3M0/IIIC | 30*25*10 | 5 | 3 | 3 | + | 1+ | 0.912 | 2.89 | 7.87 |
| P12 | M | 34 | 4 | pT4bN3M0/IIIC | 45*40*8 | 5 | 3 | 2 | + | 2+ | 1.17 | 1.09 | 2.75 |
| P13 | F | 63 | NA | pT4bN3M0/IIIC | 100*50*40 | 5 | 2-3 | 1 | + | 1+ | 0.904 | 0.616 | 4.74 |
| P14 | M | 43 | 3 | pT4N3bM0/IIIC | 50*30*10 | 5 | 3 | 3 | + | 2+ | 0.575 | 1.69 | 7.65 |
| P15 | M | 51 | 3 | pT2N0M0/IIA | 15*15*8 | 2 | 2-3 | 3 | + | 0 | 0.781 | 3.29 | 3.97 |
| P16 | M | 60 | 4 | pT3N0M0/IIA | 15*12*6 | 4 | 3 | 3 | + | 0 | 2.5 | 4.65 | 10.97 |
| P17 | F | 59 | 3/4 | pT3N3M0/IIIB | 40*40*8 | 4 | 3 | 2 | + | 0 | 3.7 | 5.48 | 0.6 |
| P18 | M | 58 | 1 | pT4N2M0/IIIB | 70*60*16 | 5 | 3 | 2 | + | 0 | 0.888 | 3.04 | 1.66 |
| P19 | M | 60 | 3 | pT1bN0M0/IA | 25*158 | 1 | 3 | 1 | + | 0 | 0.996 | 4.01 | 27.53 |
| P20 | M | 29 | 3 | pT3N3M0/IIIB | 60*40*10 | 4 | 3 | 2 | + | 1+ | 1.13 | 1.84 | 5.6 |
| P21 | M | 32 | 3 | pT4aN3M0/IIIC | 60*50*15 | 5 | 3 | 2 | + | 0 | 2.04 | 0.798 | 3.11 |
| P22 | M | 62 | 3 | pT3N3M0/IIIB | 40*30*15 | 4 | 2-3 | 3 | + | 1+ | 4.31 | 1.77 | 20.66 |
| P23 | M | 42 | 3 | pT4aN3M0/IIIC | 60*45*20 | 5 | 3 | 1 | + | 2+ | NA | NA | NA |
| P24 | M | 49 | 3 | NA | 60*50*15 | 4 | 3 | 2 | + | 0 | NA | NA | NA |
| P25 | M | 43 | 3 | pT4aN2M0/IIIB | 20*10*10 | NA | 3 | 1 | + | NA | 0.857 | 189.4 | 273.7 |
| P26 | M | 57 | 3 | pT3N3M0/III | 160*70*30 | NA | 2-3 | 1 | + | 0 | 1.44 | 2.77 | 0.807 |
| P27 | F | 35 | 3 | pT1bN0M0/IA | 110*60*30 | 1 | 3 | 3 | + | 0 | 2.45 | 0.623 | 7.78 |
| P28 | F | 52 | 3 | pT4aN2M0/IIIb | 130*115*30 | 5 | 1 | 3 | + | 1+ | 0.613 | 1.59 | 4.79 |
| P29 | M | 58 | 2/3 | pT4aN3M0/III | NA | NA | 3 | 2 | + | 0 | 1.2 | 0.869 | 4.03 |
| a: Tumor location: 1.cardia,2.fudus,3.body,4.antrum | | | |  |  |  |  |  |  |  |  |  |  |
| b: Invasion: 1.submucosa,2.muscularis mucosa,3.lamina propria,4.serosa,5.invasion through serosa or adjacent organ | | | | | | |  |  |  |  |  |  |  |
| c: Differetiation: 1.high,2.medium,3.low | | | |  |  |  |  |  |  |  |  |  |  |
| d: Lauren type: 1.intestinal,2.diffuse,3.mix | | | |  |  |  |  |  |  |  |  |  |  |

**Table S2. Sample type and analysis strategy**

| **Patient** | **Sample** | **Type^a^** | **WES^b^** | **EBV genome sequencing^b^** | **WGBS^b^** |
| --- | --- | --- | --- | --- | --- |
| P4 | P4-LD | LD | Y | N | N |
| P4 | P4-C3 | EBVaGC | Y | Y | N |
| P4 | P4-C4 | EBVaGC | Y | Y | N |
| P5 | P5-LD | LD | Y | N | N |
| P5 | P5-C1 | EBVaGC | Y | Y | N |
| P5 | P5-C2 | EBVaGC | Y | Y | N |
| P5 | P5-C3 | EBVaGC | Y | Y | N |
| P5 | P5-C4 | EBVaGC | Y | Y | N |
| P7 | P7-N | N | Y | Y | Y |
| P7 | P7-LD | LD | Y | Y | Y |
| P7 | P7-C1 | EBVaGC | Y | Y | Y |
| P7 | P7-C2 | EBVaGC | Y | Y | N |
| P7 | P7-C3 | EBVaGC | Y | Y | Y |
| P7 | P7-C4 | EBVaGC | Y | Y | N |
| P7 | P7-Sa | Saliva | N | Y | N |
| P8 | P8-N | N | Y | N | N |
| P8 | P8-LD1 | LD | Y | N | N |
| P8 | P8-LD2 | LD | Y | N | N |
| P8 | P8-C1 | EBVaGC | Y | Y | N |
| P8 | P8-C3 | EBVaGC | N | Y | N |
| P8 | P8-C4 | EBVaGC | N | Y | N |
| P9 | P9-C1 | EBVaGC | Y | Y | N |
| P9 | P9-C2 | EBVaGC | Y | Y | N |
| P9 | P9-C3 | EBVaGC | Y | Y | N |
| P9 | P9-C4 | EBVaGC | Y | Y | N |
| P10 | P10-N | N | Y | Y | N |
| P10 | P10-LD1 | LD | Y | Y | N |
| P10 | P10-LD2 | LD | Y | Y | N |
| P10 | P10-HD | HD | Y | Y | N |
| P10 | P10-C1 | EBVaGC | Y | Y | N |
| P10 | P10-C2 | EBVaGC | Y | Y | N |
| P10 | P10-C3 | EBVaGC | Y | Y | N |
| P11 | P11-N | N | Y | Y | Y |
| P11 | P11-LD2 | LD | Y | N | Y |
| P11 | P11-C1 | EBVaGC | Y | Y | N |
| P11 | P11-C2 | EBVaGC | Y | Y | N |
| P11 | P11-C3 | EBVaGC | Y | Y | Y |
| P11 | P11-C4 | EBVaGC | Y | Y | Y |
| P12 | P12-N1 | N | Y | N | N |
| P12 | P12-N2 | N | Y | N | N |
| P12 | P12-HD | HD | Y | N | N |
| P12 | P12-C1 | EBVaGC | Y | Y | N |
| P12 | P12-C2 | EBVaGC | Y | Y | N |
| P12 | P12-C3 | EBVaGC | Y | Y | N |
| P12 | P12-C4 | EBVaGC | Y | Y | N |
| P13 | P13-N1 | N | Y | Y | Y |
| P13 | P13-LD | LD | Y | Y | Y |
| P13 | P13-C1 | EBVaGC | Y | Y | Y |
| P13 | P13-C2 | EBVaGC | Y | Y | N |
| P13 | P13-C3 | EBVaGC | Y | Y | N |
| P13 | P13-C4 | EBVaGC | Y | Y | N |
| P13 | P13-C5 | EBVaGC | Y | Y | Y |
| P14 | P14-N1 | N | Y | N | N |
| P14 | P14-N2 | N | Y | N | N |
| P14 | P14-LD | LD | Y | Y | N |
| P14 | P14-C1 | EBVaGC | Y | Y | N |
| P14 | P14-C2 | EBVaGC | Y | Y | N |
| P14 | P14-C3 | EBVaGC | Y | Y | N |
| P15 | P15-N1 | N | Y | Y | N |
| P15 | P15-N2 | N | Y | N | N |
| P15 | P15-HD1 | HD | Y | Y | N |
| P15 | P15-HD2 | HD | Y | Y | N |
| P15 | P15-C1 | EBVaGC | Y | Y | N |
| P15 | P15-C2 | EBVaGC | Y | Y | N |
| P15 | P15-C3 | EBVaGC | Y | Y | N |
| P15 | P15-C4 | EBVaGC | Y | Y | N |
| P16 | P16-N | N | Y | Y | N |
| P16 | P16-LD1 | LD | Y | Y | N |
| P16 | P16-LD2 | LD | Y | N | N |
| P16 | P16-C1 | EBVaGC | Y | Y | N |
| P16 | P16-C3 | EBVaGC | Y | Y | N |
| P16 | P16-C4 | EBVaGC | Y | Y | N |
| P17 | P17-N2 | N | Y | N | N |
| P17 | P17-LD | LD | Y | N | N |
| P17 | P17-C1 | EBVaGC | Y | Y | N |
| P17 | P17-C2 | EBVaGC | Y | Y | N |
| P17 | P17-C3 | EBVaGC | Y | Y | N |
| P17 | P17-Sa | Saliva | N | Y | N |
| P18 | P18-HD | HD | Y | N | N |
| P18 | P18-C1 | EBVaGC | Y | Y | N |
| P18 | P18-C2 | EBVaGC | Y | Y | N |
| P18 | P18-C3 | EBVaGC | Y | N | N |
| P18 | P18-Sa | Saliva | N | Y | N |
| P19 | P19-N | N | Y | Y | N |
| P19 | P19-LD1 | LD | Y | Y | N |
| P19 | P19-LD2 | LD | Y | N | N |
| P19 | P19-C1 | EBVaGC | Y | Y | N |
| P19 | P19-C2 | EBVaGC | Y | Y | N |
| P20 | P20-N | N | Y | N | N |
| P20 | P20-LD1 | LD | Y | N | N |
| P20 | P20-LD2 | LD | Y | N | N |
| P20 | P20-C1 | EBVaGC | Y | Y | N |
| P20 | P20-C2 | EBVaGC | Y | Y | N |
| P21 | P21-N | N | Y | N | N |
| P21 | P21-LD1 | LD | Y | N | N |
| P21 | P21-LD2 | LD | Y | N | N |
| P21 | P21-C2 | EBVaGC | Y | Y | N |
| P21 | P21-C3 | EBVaGC | Y | Y | N |
| P21 | P21-Sa | Saliva | N | Y | N |
| P22 | P22-HD | HD | Y | Y | N |
| P22 | P22-C1 | EBVaGC | Y | Y | N |
| P22 | P22-C2 | EBVaGC | Y | Y | N |
| P22 | P22-C3 | EBVaGC | Y | Y | N |
| P22 | P22-Sa | Saliva | N | Y | N |
| P23 | P23-N | N | Y | N | N |
| P23 | P23-HD | HD | Y | Y | N |
| P23 | P23-C1 | EBVaGC | Y | Y | N |
| P23 | P23-C2 | EBVaGC | Y | Y | N |
| P23 | P23-C3 | EBVaGC | Y | Y | N |
| P23 | P23-C4 | EBVaGC | Y | Y | N |
| P24 | P24-HD1 | HD | Y | N | N |
| P24 | P24-HD2 | HD | Y | N | N |
| P24 | P24-C1 | EBVaGC | Y | N | N |
| P24 | P24-C2 | EBVaGC | Y | N | N |
| P25 | P25-C | EBVaGC | N | Y | N |
| P25 | P25-Sa | Saliva | N | Y | N |
| P26 | P26-C | EBVaGC | N | Y | N |
| P26 | P26-Sa | Saliva | N | Y | N |
| P27 | P27-C | EBVaGC | N | Y | N |
| P27 | P27-Sa | Saliva | N | Y | N |
| P28 | P28-C | EBVaGC | N | Y | N |
| P28 | P28-Sa | Saliva | N | Y | N |
| P29 | P29-Sa | Saliva | N | Y | N |

a: N,morphalogically normal epithelial tissues; LD,low-grade dysplasia; HD,high-grade dysplasia; EBVaGC, EBV-associated gastric carcinoma

b:Y,yes; N,No

**Table S3. Sequencing information of all samples (Exome sequencing information)**

| **Sample^a^** | **Avergae_sequencing_depth_on_target** | **Coverage_of_target_region** | **Fraction_of_target_covered_with_at_least_20x** | **Fraction_of_target_covered_with_at_least_10x** | **Fraction_of_target_covered_with_at_least_4x** |
| --- | --- | --- | --- | --- | --- |
| P10-B | 65.82 | 99.80% | 95.80% | 98.90% | 99.50% |
| P10-C1 | 117.75 | 99.90% | 97.90% | 99.20% | 99.70% |
| P10-C2 | 124.49 | 99.90% | 98.00% | 99.30% | 99.80% |
| P10-C3 | 147.41 | 99.90% | 98.50% | 99.40% | 99.80% |
| P10-HD | 124.61 | 99.90% | 98.30% | 99.40% | 99.80% |
| P10-LD1 | 216.57 | 99.90% | 99.30% | 99.70% | 99.90% |
| P10-LD2 | 121.92 | 99.90% | 98.30% | 99.30% | 99.70% |
| P10-N | 106.14 | 99.90% | 98.00% | 99.40% | 99.80% |
| P11-B | 61.13 | 99.80% | 95.60% | 98.70% | 99.50% |
| P11-C1 | 113.15 | 99.90% | 97.40% | 99.10% | 99.70% |
| P11-C2 | 84.26 | 99.60% | 90.40% | 96.20% | 98.60% |
| P11-C3 | 93.12 | 99.80% | 96.00% | 98.50% | 99.50% |
| P11-C4 | 95.89 | 99.80% | 96.00% | 98.50% | 99.50% |
| P11-LD2 | 75.73 | 99.80% | 94.60% | 98.10% | 99.40% |
| P11-N | 124.44 | 99.90% | 97.30% | 98.90% | 99.60% |
| P12-B | 80.4 | 99.90% | 98.00% | 99.30% | 99.70% |
| P12-C1 | 82.29 | 99.90% | 96.50% | 98.90% | 99.70% |
| P12-C2 | 132.16 | 99.90% | 97.80% | 99.20% | 99.70% |
| P12-C3 | 79.44 | 99.70% | 95.80% | 98.30% | 99.30% |
| P12-C4 | 113.16 | 99.90% | 96.90% | 99.00% | 99.70% |
| P12-HD | 114.99 | 99.90% | 97.40% | 99.10% | 99.70% |
| P12-N1 | 85.54 | 99.90% | 97.00% | 99.00% | 99.70% |
| P12-N2 | 89.15 | 99.90% | 97.10% | 99.10% | 99.70% |
| P13-B | 121.16 | 99.80% | 98.40% | 99.30% | 99.70% |
| P13-C1 | 126.88 | 99.80% | 98.20% | 99.30% | 99.60% |
| P13-C2 | 114.13 | 99.80% | 98.40% | 99.30% | 99.70% |
| P13-C3 | 109.81 | 99.80% | 98.10% | 99.20% | 99.60% |
| P13-C4 | 123.49 | 99.80% | 98.60% | 99.30% | 99.60% |
| P13-C5 | 106.41 | 99.80% | 97.90% | 99.10% | 99.60% |
| P13-LD | 102.54 | 98.60% | 94.90% | 96.70% | 97.70% |
| P13-N1 | 117.12 | 99.80% | 98.30% | 99.30% | 99.70% |
| P14-B | 95.98 | 99.90% | 98.30% | 99.30% | 99.70% |
| P14-C1 | 100.07 | 99.90% | 97.00% | 98.90% | 99.60% |
| P14-C2 | 114.71 | 99.90% | 97.50% | 99.00% | 99.60% |
| P14-C3 | 106.78 | 99.90% | 97.60% | 99.10% | 99.70% |
| P14-LD | 58.71 | 99.80% | 92.00% | 97.70% | 99.30% |
| P14-N1 | 126.53 | 99.90% | 97.60% | 99.00% | 99.60% |
| P14-N2 | 104.64 | 99.90% | 97.20% | 98.90% | 99.60% |
| P15-B | 95.02 | 99.90% | 98.50% | 99.40% | 99.70% |
| P15-C1 | 109.46 | 99.90% | 98.00% | 99.20% | 99.70% |
| P15-C2 | 100.79 | 99.90% | 98.00% | 99.30% | 99.70% |
| P15-C3 | 128.46 | 99.90% | 98.50% | 99.30% | 99.70% |
| P15-C4 | 104.78 | 99.90% | 98.20% | 99.30% | 99.70% |
| P15-HD1 | 99.11 | 99.90% | 97.20% | 98.90% | 99.60% |
| P15-HD2 | 102.85 | 99.90% | 97.60% | 99.10% | 99.70% |
| P15-N1 | 104.73 | 99.90% | 98.00% | 99.20% | 99.70% |
| P15-N2 | 102.39 | 99.80% | 97.40% | 98.90% | 99.50% |
| P16-B | 58.97 | 99.90% | 93.80% | 98.40% | 99.60% |
| P16-C1 | 101.36 | 99.90% | 96.20% | 98.60% | 99.60% |
| P16-LD2 | 101.11 | 99.90% | 96.00% | 98.50% | 99.50% |
| P16-C3 | 87.77 | 99.90% | 95.20% | 98.20% | 99.50% |
| P16-C4 | 115.77 | 99.90% | 96.80% | 98.80% | 99.60% |
| P16-LD1 | 101.14 | 99.90% | 96.00% | 98.50% | 99.50% |
| P16-N | 107.58 | 99.90% | 97.00% | 98.90% | 99.70% |
| P17-B | 77.33 | 99.80% | 96.90% | 99.00% | 99.60% |
| P17-C1 | 119.97 | 99.80% | 97.60% | 99.10% | 99.70% |
| P17-C2 | 89.7 | 99.80% | 96.80% | 98.90% | 99.60% |
| P17-C3 | 98.16 | 99.70% | 96.60% | 98.60% | 99.40% |
| P17-LD | 91.47 | 99.80% | 96.90% | 98.90% | 99.60% |
| P17-N2 | 90.89 | 99.80% | 96.50% | 98.80% | 99.60% |
| P18-B | 90.62 | 99.90% | 98.00% | 99.40% | 99.80% |
| P18-C1 | 93.1 | 99.90% | 97.40% | 99.20% | 99.80% |
| P18-C2 | 94.8 | 99.90% | 96.90% | 99.00% | 99.70% |
| P18-C3 | 51.17 | 99.90% | 93.90% | 98.50% | 99.70% |
| P18-HD | 75.77 | 99.90% | 96.00% | 98.80% | 99.70% |
| P19-B | 70.77 | 99.90% | 96.10% | 98.90% | 99.60% |
| P19-C1 | 121.67 | 99.90% | 98.30% | 99.40% | 99.80% |
| P19-C2 | 130.54 | 99.90% | 97.90% | 99.20% | 99.70% |
| P19-LD1 | 99.09 | 99.90% | 97.70% | 99.30% | 99.80% |
| P19-LD2 | 113.59 | 99.90% | 97.90% | 99.30% | 99.80% |
| P19-N | 80.67 | 99.90% | 96.50% | 98.90% | 99.70% |
| P20-B | 77.01 | 99.90% | 96.80% | 99.00% | 99.60% |
| P20-C1 | 139.29 | 99.80% | 97.90% | 99.10% | 99.60% |
| P20-C2 | 98.27 | 99.90% | 97.10% | 98.90% | 99.60% |
| P20-LD1 | 108.72 | 99.90% | 97.80% | 99.30% | 99.80% |
| P20-LD2 | 105.06 | 99.90% | 97.40% | 99.10% | 99.70% |
| P20-N | 106.24 | 99.90% | 97.80% | 99.20% | 99.70% |
| P21-B | 74.7 | 99.80% | 96.40% | 98.80% | 99.50% |
| P21-LD2 | 97.21 | 99.90% | 97.40% | 99.10% | 99.70% |
| P21-C2 | 109.36 | 99.90% | 97.40% | 99.10% | 99.70% |
| P21-C3 | 112.26 | 99.90% | 96.90% | 98.80% | 99.60% |
| P21-LD1 | 74.87 | 99.90% | 95.50% | 98.70% | 99.60% |
| P21-N | 98.29 | 99.90% | 97.30% | 99.00% | 99.60% |
| P22-B | 87.2 | 99.90% | 97.50% | 99.30% | 99.80% |
| P22-C1 | 105.34 | 99.90% | 97.20% | 99.00% | 99.70% |
| P22-C2 | 78.87 | 99.90% | 95.90% | 98.70% | 99.60% |
| P22-C3 | 84.84 | 99.90% | 96.30% | 98.80% | 99.60% |
| P22-HD | 89.08 | 99.90% | 96.40% | 98.80% | 99.70% |
| P23-B | 75.45 | 99.90% | 96.80% | 99.10% | 99.70% |
| P23-C1 | 102.66 | 99.90% | 97.20% | 99.00% | 99.70% |
| P23-C2 | 78.63 | 99.90% | 95.20% | 98.50% | 99.60% |
| P23-C3 | 88.14 | 99.90% | 95.80% | 98.60% | 99.60% |
| P23-C4 | 104.45 | 99.90% | 97.40% | 99.10% | 99.70% |
| P23-HD | 91.99 | 99.90% | 96.50% | 98.80% | 99.70% |
| P23-N | 99.68 | 99.90% | 96.80% | 98.90% | 99.70% |
| P24-B | 205.49 | 100.00% | 99.60% | 99.80% | 99.90% |
| P24-C1 | 115.85 | 99.80% | 96.50% | 98.40% | 99.40% |
| P24-C2 | 95.66 | 99.80% | 94.90% | 97.70% | 99.20% |
| P24-HD1 | 102.35 | 99.80% | 95.70% | 98.10% | 99.30% |
| P24-HD2 | 116.36 | 99.80% | 96.50% | 98.50% | 99.50% |
| P4-B | 70.8 | 99.90% | 96.50% | 99.00% | 99.60% |
| P4-C3 | 84.65 | 99.80% | 95.60% | 98.30% | 99.40% |
| P4-C4 | 80.17 | 99.80% | 95.00% | 98.10% | 99.30% |
| P4-LD | 94.01 | 99.90% | 96.80% | 98.80% | 99.60% |
| P5-B | 114.85 | 99.80% | 96.50% | 98.60% | 99.50% |
| P5-C1 | 121.18 | 99.90% | 97.80% | 99.10% | 99.70% |
| P5-C2 | 91.62 | 99.80% | 95.40% | 98.10% | 99.40% |
| P5-C3 | 110.37 | 99.80% | 96.40% | 98.50% | 99.50% |
| P5-C4 | 164.41 | 99.90% | 98.40% | 99.40% | 99.80% |
| P5-LD | 120.15 | 99.90% | 96.90% | 98.80% | 99.60% |
| P7-B | 95.54 | 99.90% | 98.00% | 99.40% | 99.80% |
| P7-C1 | 77.99 | 99.80% | 95.70% | 98.40% | 99.40% |
| P7-C2 | 75.62 | 99.90% | 95.70% | 98.50% | 99.50% |
| P7-C3 | 76.24 | 99.80% | 95.20% | 98.30% | 99.40% |
| P7-C4 | 73.05 | 99.90% | 96.30% | 98.80% | 99.60% |
| P7-LD | 79.45 | 99.90% | 96.60% | 98.90% | 99.60% |
| P7-N | 71.69 | 99.90% | 96.00% | 98.70% | 99.60% |
| P8-B | 61.62 | 99.90% | 94.10% | 98.20% | 99.50% |
| P8-C1 | 104.78 | 99.90% | 97.40% | 99.10% | 99.70% |
| P8-LD2 | 100.15 | 99.90% | 97.50% | 99.10% | 99.70% |
| P8-LD1 | 88.15 | 99.90% | 96.60% | 98.80% | 99.60% |
| P8-N | 84.4 | 99.90% | 96.50% | 98.90% | 99.70% |
| P9-B | 64.34 | 99.80% | 96.00% | 98.80% | 99.50% |
| P9-C1 | 111.19 | 99.90% | 96.50% | 98.60% | 99.60% |
| P9-C2 | 88.02 | 99.80% | 94.80% | 98.00% | 99.40% |
| P9-C3 | 106.38 | 99.90% | 96.40% | 98.60% | 99.60% |
| P9-C4 | 106.51 | 99.80% | 96.20% | 98.40% | 99.40% |
| a:Names of control samples, including 2 distant normal gastric tissues (≥ 5 cm from tumor site; P5 and P13) and 18 blood samples, are in the form of Patient identification + B | | | | | |

**Table S4. Sequencing information of all samples (EBV genome information)**

| **Sample** | **1st ranking reference mapped reads(frequency)^a^** | **2nd ranking reference mapped reads(frequency)^b^** | **3rd ranking reference mapped reads(frequency)^c^** | **4th ranking reference mapped reads(frequency)^d^** | **5th ranking reference mapped reads(frequency)^e^** | **Mapped paired-end reads^f^** | **Mapping rate** |
| --- | --- | --- | --- | --- | --- | --- | --- |
| EP10-C1 | AB850649.1 466325(0.57) | Others 120018(0.15) | AB850658.1 96996(0.12) | AY961628.3 71516(0.09) | AP015015.1 67641(0.08) | 41274768 | 87.04% |
| EP10-C2 | AB850649.1 465190(0.56) | Others 129467(0.15) | AB850658.1 95851(0.11) | AY961628.3 77784(0.09) | AP015015.1 69206(0.08) | 41872058 | 86.97% |
| EP10-C3 | AB850649.1 408991(0.60) | Others 90082(0.13) | AB850658.1 73658(0.11) | AY961628.3 58180(0.08) | AP015015.1 55413(0.08) | 37353556 | 86.75% |
| EP10-HD2 | AB850649.1 506344(0.59) | Others 118770(0.14) | AB850658.1 81927(0.10) | AY961628.3 73239(0.09) | AP015015.1 72795(0.09) | 44129476 | 86.57% |
| EP10-LD1 | LN824142.1 1427518(0.66) | KC207813.1 354201(0.16) | AP015016.1 294286(0.14) | Others 45779(0.02) | KF992570.1 29659(0.01) | 79153530 | 85.45% |
| EP10-LD2 | LN824142.1 1784214(0.68) | AP015016.1 415692(0.16) | Others 241794(0.09) | KC207813.1 119676(0.05) | AB850653.1 76546(0.03) | 115343866 | 87.53% |
| EP10-N | LN824142.1 72676(0.77) | AB850660.1 9036(0.10) | Others 4866(0.05) | AP015016.1 4506(0.05) | LN827596.1 3065(0.03) | 16177506 | 33.25% |
| EP11-C1 | JQ009376.2 13085(0.68) | Others 2950(0.15) | KF373730.1 1515(0.08) | HQ020558.1 1302(0.07) | LC137018.1 445(0.02) | 30206528 | 95.31% |
| EP11-C2 | JQ009376.2 6928(0.52) | Others 3355(0.25) | KF373730.1 1454(0.11) | HQ020558.1 1014(0.08) | LN827563.2 536(0.04) | 36722380 | 96.23% |
| EP11-C3 | JQ009376.2 13408(0.65) | Others 3529(0.17) | KF373730.1 1812(0.09) | HQ020558.1 1505(0.07) | LC137018.1 495(0.02) | 32843210 | 95.32% |
| EP11-C4 | JQ009376.2 21830(0.74) | HQ020558.1 2518(0.09) | Others 2494(0.08) | KF373730.1 1804(0.06) | LC137018.1 847(0.03) | 26728076 | 95.67% |
| EP11-N | LN824142.1 672(0.46) | Others 319(0.22) | AP015016.1 254(0.17) | LC137018.1 111(0.08) | KC207813.1 102(0.07) | 8826416 | 16.65% |
| EP12-C1 | KF992569.1 22447(0.40) | AB850648.1 16225(0.29) | Others 8186(0.15) | JQ009376.2 6471(0.11) | LC137018.1 2950(0.05) | 36913064 | 95.04% |
| EP12-C2 | KF992569.1 25393(0.38) | AB850648.1 17870(0.27) | Others 9686(0.15) | JQ009376.2 8821(0.13) | LC137018.1 4239(0.06) | 46470790 | 95.40% |
| EP12-C3 | KF992569.1 21420(0.42) | AB850648.1 10156(0.20) | Others 8928(0.18) | JQ009376.2 7376(0.15) | KF373730.1 2754(0.05) | 41612604 | 95.91% |
| EP12-C4 | KF992569.1 23663(0.42) | AB850648.1 16619(0.29) | Others 8226(0.15) | JQ009376.2 5824(0.10) | KF373730.1 2010(0.04) | 43238926 | 95.49% |
| EP13-C1 | AP015016.1 487082(0.86) | AP015015.1 62395(0.11) | Others 5778(0.01) | AB850649.1 5316(0.01) | KF992565.1 4191(0.01) | 31713802 | 96.02% |
| EP13-C2 | AP015016.1 763253(0.85) | AP015015.1 109609(0.12) | AB850649.1 8752(0.01) | Others 8750(0.01) | KF992565.1 6154(0.01) | 48697610 | 96.45% |
| EP13-C3 | AP015016.1 1003616(0.87) | AP015015.1 136990(0.12) | AB850649.1 10494(0.01) | Others 5777(0.00) | KF992565.1 2383(0.00) | 48052170 | 97.13% |
| EP13-C4 | AP015016.1 1020401(0.87) | AP015015.1 134397(0.11) | AB850649.1 11019(0.01) | Others 5289(0.00) | KF992570.1 799(0.00) | 42963280 | 97.06% |
| EP13-C5 | AP015016.1 1237846(0.88) | AP015015.1 148378(0.11) | AB850649.1 12659(0.01) | Others 5896(0.00) | AB850644.1 852(0.00) | 51504322 | 97.38% |
| EP13-LD | AP015016.1 497830(0.55) | AP015015.1 394304(0.44) | KC617875.1 6363(0.01) | LN827589.1 4410(0.00) | Others 3350(0.00) | 59688268 | 82.70% |
| EP13-N1 | AP015016.1 1158095(0.87) | AP015015.1 162251(0.12) | Others 10025(0.01) | LN827589.1 3817(0.00) | LN827563.2 3077(0.00) | 93754740 | 88.87% |
| EP14-C1 | AB850646.1 165312(0.72) | AB850653.1 35274(0.15) | JQ009376.2 20137(0.09) | Others 7014(0.03) | AP015016.1 1460(0.01) | 35599584 | 79.87% |
| EP14-C2 | AB850646.1 154389(0.79) | AB850653.1 21526(0.11) | JQ009376.2 13642(0.07) | Others 5975(0.03) | AP015016.1 1047(0.01) | 33338360 | 79.95% |
| EP14-C3 | AB850646.1 166864(0.74) | AB850653.1 32227(0.14) | JQ009376.2 19256(0.09) | Others 6723(0.03) | AP015016.1 1425(0.01) | 36570014 | 80.05% |
| EP14-LD | AB850646.1 120033(0.56) | AB850653.1 54540(0.26) | KF992564.1 15194(0.07) | JQ009376.2 11654(0.05) | Others 11068(0.05) | 40458928 | 70.84% |
| EP15-C1 | AB850658.1 163262(0.43) | Others 71943(0.19) | AP015016.1 61518(0.16) | KC207813.1 48603(0.13) | AB850643.1 30144(0.08) | 33486906 | 64.88% |
| EP15-C2 | AB850658.1 141996(0.42) | Others 71126(0.21) | AP015016.1 50425(0.15) | KC207813.1 44041(0.13) | AB850643.1 26609(0.08) | 30575240 | 65.54% |
| EP15-C3 | AB850658.1 157860(0.43) | Others 74506(0.20) | AP015016.1 57879(0.16) | KC207813.1 47997(0.13) | AB850643.1 29103(0.08) | 33957596 | 65.07% |
| EP15-C4 | AB850658.1 163602(0.46) | Others 69142(0.19) | AP015016.1 54671(0.15) | KC207813.1 40785(0.11) | AB850643.1 30433(0.08) | 31171244 | 64.91% |
| EP15-HD1 | AB850658.1 140286(0.42) | Others 67350(0.20) | AP015016.1 54327(0.16) | KC207813.1 42187(0.13) | AB850643.1 28572(0.09) | 29214720 | 64.84% |
| EP15-HD2 | AB850658.1 157830(0.42) | Others 74429(0.20) | AP015016.1 64328(0.17) | KC207813.1 47156(0.12) | AB850643.1 35600(0.09) | 31713586 | 63.49% |
| EP15-N1 | LN824142.1 579584(0.98) | AB850649.1 8031(0.01) | Others 1077(0.00) | AP015016.1 455(0.00) | LN824226.1 263(0.00) | 15552256 | 22.11% |
| EP16-C1 | JQ009376.2 16200(0.54) | Others 9456(0.32) | KF373730.1 2132(0.07) | KF992564.1 1086(0.04) | LN824224.1 987(0.03) | 58637044 | 95.27% |
| EP16-C3 | JQ009376.2 17225(0.51) | Others 12088(0.36) | KF373730.1 2076(0.06) | KF992564.1 1287(0.04) | KC617875.1 1024(0.03) | 66755268 | 93.67% |
| EP16-C4 | JQ009376.2 2921(0.51) | Others 1925(0.33) | KF373730.1 430(0.07) | LN824224.1 300(0.05) | KC617875.1 194(0.03) | 13549898 | 94.77% |
| EP16-LD1 | KF992564.1 165061(0.57) | KC617875.1 104264(0.36) | Others 10081(0.03) | KF373730.1 8377(0.03) | JQ009376.2 3443(0.01) | 76555694 | 92.26% |
| EP16-N | KC207813.1 29009(0.36) | KF992564.1 25635(0.32) | Others 11302(0.14) | LN824209.1 9258(0.12) | AB850643.1 5132(0.06) | 57161594 | 74.50% |
| EP17-C1 | AB850653.1 396565(0.77) | AB850646.1 40926(0.08) | Others 40313(0.08) | KF992570.1 20695(0.04) | LN824142.1 17054(0.03) | 45572936 | 77.30% |
| EP17-C2 | AB850653.1 461825(0.78) | AB850646.1 49218(0.08) | Others 42088(0.07) | KF992570.1 23418(0.04) | LN824142.1 16141(0.03) | 51572636 | 77.14% |
| EP17-C3 | AB850653.1 498220(0.78) | AB850646.1 46521(0.07) | Others 44040(0.07) | KF992570.1 27197(0.04) | LN824142.1 20824(0.03) | 57158620 | 78.05% |
| EP17-Sa | LN824142.1 36108(0.57) | Others 10355(0.16) | KC207813.1 7519(0.12) | AB850658.1 6111(0.10) | AP015015.1 3229(0.05) | 10358850 | 63.18% |
| EP18-C1 | LN824224.1 1064293(0.95) | Others 30877(0.03) | KC617875.1 14612(0.01) | AB850648.1 8622(0.01) | JQ009376.2 6333(0.01) | 55067780 | 88.19% |
| EP18-C2 | LN824224.1 1167438(0.95) | Others 31711(0.03) | KC617875.1 13806(0.01) | AB850648.1 8082(0.01) | LN827564.1 6619(0.01) | 59250842 | 88.38% |
| EP18-Sa | LN824142.1 37583(0.48) | Others 15061(0.19) | KC207813.1 12270(0.16) | AB850658.1 8795(0.11) | AP015015.1 5314(0.07) | 10515928 | 71.80% |
| EP19-C1 | LN824224.1 693100(0.34) | Others 471432(0.23) | AB850649.1 352444(0.17) | LN824142.1 293263(0.14) | LN824209.1 235524(0.12) | 50257992 | 86.25% |
| EP19-C2 | LN824224.1 798402(0.35) | Others 536797(0.23) | AB850649.1 381647(0.17) | LN824142.1 335716(0.15) | LN824209.1 237672(0.10) | 59532948 | 86.57% |
| EP19-LD1 | LN824142.1 705(0.56) | AP015016.1 336(0.27) | Others 105(0.08) | AP015015.1 53(0.04) | AB850646.1 50(0.04) | 1355540 | 2.72% |
| EP19-N | LN824142.1 792(0.61) | AP015016.1 322(0.25) | Others 91(0.07) | AP015015.1 60(0.05) | AB850646.1 31(0.02) | 218526 | 0.45% |
| EP20-C1 | Others 14414(0.34) | AB850657.1 13482(0.32) | JQ009376.2 7886(0.19) | KF373730.1 3688(0.09) | KC617875.1 2617(0.06) | 50230292 | 80.09% |
| EP20-C2 | Others 17162(0.35) | AB850657.1 13224(0.27) | JQ009376.2 10651(0.22) | KF373730.1 4656(0.10) | KC617875.1 2968(0.06) | 54860846 | 81.51% |
| EP21-C2 | Others 29573(0.34) | AB850657.1 23594(0.27) | AB850648.1 16349(0.19) | JQ009376.2 11750(0.14) | HQ020558.1 5262(0.06) | 74211850 | 80.34% |
| EP21-C3 | Others 20746(0.36) | AB850657.1 18437(0.32) | AB850648.1 9865(0.17) | JQ009376.2 5746(0.10) | HQ020558.1 2506(0.04) | 58630374 | 80.27% |
| EP21-Sa | LN824142.1 17829(0.51) | KC207813.1 6937(0.20) | Others 4791(0.14) | AB850658.1 3347(0.10) | AB850643.1 1897(0.05) | 4946818 | 62.67% |
| EP22-C1 | AY961628.3 206046(0.52) | AP015015.1 64340(0.16) | KC207813.1 57986(0.15) | LN824142.1 37961(0.09) | Others 33441(0.08) | 63382230 | 92.15% |
| EP22-C2 | AY961628.3 268518(0.49) | AP015015.1 91419(0.17) | KC207813.1 87768(0.16) | LN824142.1 48841(0.09) | Others 48041(0.09) | 82423410 | 93.51% |
| EP22-C3 | AY961628.3 296821(0.53) | AP015015.1 84261(0.15) | KC207813.1 79707(0.14) | Others 48826(0.09) | LN824142.1 45856(0.08) | 88222232 | 94.63% |
| EP22-HD | AY961628.3 270368(0.50) | KC207813.1 90891(0.17) | AP015015.1 86969(0.16) | Others 47970(0.09) | LN824142.1 44157(0.08) | 75272858 | 94.45% |
| EP22-Sa | AP015016.1 18567(0.82) | AB850645.1 3233(0.14) | Others 495(0.02) | AB850650.1 259(0.01) | AY961628.3 204(0.01) | 1738238 | 4.59% |
| EP23-C1 | LC137018.1 16757(0.36) | JQ009376.2 14033(0.30) | Others 10538(0.23) | KF373730.1 3547(0.08) | AB850643.1 1485(0.03) | 72246586 | 94.62% |
| EP23-C2 | LC137018.1 21786(0.39) | JQ009376.2 16232(0.29) | Others 11899(0.21) | KF373730.1 4143(0.07) | AB850643.1 2052(0.04) | 88764532 | 94.30% |
| EP23-C3 | LC137018.1 25323(0.41) | JQ009376.2 17431(0.28) | Others 13127(0.21) | KF373730.1 4597(0.07) | AB850643.1 1645(0.03) | 81747972 | 93.85% |
| EP23-C4 | LC137018.1 24867(0.41) | JQ009376.2 17346(0.29) | Others 11631(0.19) | KF373730.1 4113(0.07) | AB850643.1 2174(0.04) | 79739820 | 94.87% |
| EP23-HD | LC137018.1 20838(0.44) | JQ009376.2 14916(0.31) | Others 7637(0.16) | KF373730.1 2182(0.05) | AB850643.1 1886(0.04) | 38568670 | 94.02% |
| EP25-C | AP015016.1 450919(0.44) | AB850650.1 273746(0.27) | LN824142.1 114383(0.11) | Others 99699(0.10) | KC207813.1 90389(0.09) | 62985220 | 93.25% |
| EP25-Sa | AP015016.1 23174(0.31) | AB850650.1 23135(0.31) | LN824142.1 11706(0.15) | Others 9959(0.13) | KC207813.1 7812(0.10) | 5134698 | 11.08% |
| EP26-C | Others 34931(0.30) | JQ009376.2 30418(0.26) | HQ020558.1 23709(0.20) | KC617875.1 16801(0.14) | LC137018.1 12454(0.11) | 70866184 | 95.82% |
| EP26-Sa | AP015016.1 287(0.33) | Others 275(0.31) | AB850650.1 180(0.20) | LN824142.1 84(0.10) | KC207813.1 57(0.06) | 245162 | 0.57% |
| EP27-C | AP015016.1 585554(0.43) | AB850644.1 297546(0.22) | AB850643.1 219068(0.16) | Others 198158(0.14) | AB850658.1 68042(0.05) | 145698352 | 93.32% |
| EP27-Sa | LN824209.1 767787(0.62) | Others 201531(0.16) | AP015016.1 112047(0.09) | AP015015.1 82077(0.07) | AB850658.1 71775(0.06) | 92591462 | 82.47% |
| EP28-C | LN827563.2 350833(0.85) | Others 37712(0.09) | KF992571.1 11923(0.03) | JQ009376.2 7056(0.02) | KC617875.1 5311(0.01) | 229508974 | 79.15% |
| EP28-Sa | Others 7963(0.30) | LN824209.1 7961(0.30) | AP015016.1 5273(0.20) | LN827563.2 3344(0.12) | AB850644.1 2239(0.08) | 4850704 | 34.99% |
| EP29-Sa | Others 21860(0.45) | AB850648.1 11676(0.24) | KF992569.1 6455(0.13) | AP015016.1 4241(0.09) | LN824209.1 3844(0.08) | 43468502 | 64.83% |
| EP4-C3 | AB850644.1 333525(0.55) | AB850649.1 164431(0.27) | AB850658.1 51244(0.09) | Others 26291(0.04) | KC440851.1 26033(0.04) | 10111194 | 79.44% |
| EP4-C4 | AB850644.1 615665(0.44) | AB850649.1 390018(0.28) | AB850658.1 233089(0.16) | Others 106943(0.08) | KC440851.1 68970(0.05) | 35165180 | 81.47% |
| EP5-C1 | AB850643.1 109197(0.33) | AB850646.1 89222(0.27) | AB850660.1 56905(0.17) | AB850649.1 46572(0.14) | Others 25580(0.08) | 21288696 | 77.32% |
| EP5-C2 | AB850643.1 90094(0.30) | AB850646.1 84719(0.28) | AB850649.1 50536(0.17) | AB850660.1 47490(0.16) | Others 28238(0.09) | 20056820 | 80.10% |
| EP5-C3 | AB850643.1 139405(0.36) | AB850646.1 106145(0.27) | AB850660.1 67256(0.17) | AB850649.1 45560(0.12) | Others 28206(0.07) | 23156776 | 81.82% |
| EP5-C4 | AB850643.1 117494(0.34) | AB850646.1 91376(0.27) | AB850649.1 54490(0.16) | AB850660.1 53771(0.16) | Others 27366(0.08) | 22503170 | 76.61% |
| EP7-C1 | AP015016.1 340721(0.60) | AB850649.1 86656(0.15) | KC207813.1 53686(0.09) | Others 45920(0.08) | AP015015.1 40002(0.07) | 52113828 | 90.89% |
| EP7-C2 | AP015016.1 284927(0.59) | AB850649.1 73776(0.15) | KC207813.1 45802(0.09) | Others 42556(0.09) | AP015015.1 37938(0.08) | 44263488 | 94.74% |
| EP7-C3 | AP015016.1 236200(0.58) | AB850649.1 62740(0.15) | KC207813.1 43157(0.11) | Others 37008(0.09) | AP015015.1 30158(0.07) | 37322842 | 94.07% |
| EP7-C4 | AP015016.1 238521(0.60) | AB850649.1 60357(0.15) | KC207813.1 38889(0.10) | Others 32613(0.08) | AP015015.1 26053(0.07) | 37251364 | 94.92% |
| EP7-LD | AP015016.1 242013(0.89) | AP015015.1 13156(0.05) | Others 7634(0.03) | LN827589.1 4200(0.02) | LN827523.1 4124(0.02) | 21049466 | 40.86% |
| EP7-N | LN824142.1 682(0.37) | Others 367(0.20) | AY961628.3 340(0.18) | AP015016.1 242(0.13) | AB850650.1 233(0.12) | 7157856 | 16.71% |
| EP7-Sa | LN824142.1 87630(0.54) | Others 26920(0.17) | KC207813.1 18271(0.11) | AP015016.1 16796(0.10) | AB850658.1 12465(0.08) | 20679616 | 80.64% |
| EP8-C1 | Others 32635(0.44) | JQ009376.2 16575(0.22) | KF992565.1 12362(0.17) | LC137018.1 6725(0.09) | LN827548.2 6330(0.08) | 73686120 | 94.51% |
| EP8-C3 | Others 24213(0.45) | KF992565.1 10472(0.19) | JQ009376.2 10354(0.19) | LN827548.2 4818(0.09) | LC137018.1 3944(0.07) | 47892166 | 94.05% |
| EP8-C4 | Others 23521(0.46) | JQ009376.2 11376(0.22) | KF992565.1 7266(0.14) | LC137018.1 5232(0.10) | LN827548.2 4132(0.08) | 50238038 | 91.99% |
| EP9-C1 | KF992565.1 21025(0.59) | Others 7222(0.20) | JQ009376.2 3443(0.10) | KF373730.1 2050(0.06) | AB850653.1 1860(0.05) | 53244930 | 96.49% |
| EP9-C2 | KF992565.1 19854(0.63) | Others 5365(0.17) | JQ009376.2 3247(0.10) | KF373730.1 1679(0.05) | AB850653.1 1433(0.05) | 45320934 | 96.03% |
| EP9-C3 | KF992565.1 17140(0.63) | Others 4358(0.16) | JQ009376.2 2603(0.10) | AB850653.1 1690(0.06) | KF373730.1 1545(0.06) | 36629184 | 96.47% |
| EP9-C4 | KF992565.1 13137(0.61) | Others 3470(0.16) | JQ009376.2 2214(0.10) | KF373730.1 1357(0.06) | AB850653.1 1239(0.06) | 33511152 | 96.44% |
| a-e: Top 4 ranked EBV references and all others when mapping to the EBV reference panel (reference name + uniquely mapped paired-end reads(frequency) | | | | | |  |  |
| f: Mapped paired-end reads to the top 1 ranked reference without remove of duplicates | | | |  |  |  |  |

**Table S5. Sequencing information of all samples (WGBS information)**

| **Sample** | **Sample type** | **Mapping rate** | **Covergae_human(hg19)** | **Coverage_EBV(NC_007605)** | **CpG methylated rate** | **CHG methylated rate** | **CHH methylated rate** |
| --- | --- | --- | --- | --- | --- | --- | --- |
| P7-N | N | 56.75% | 12.02 | 0.12 | 73.88% | 26.97% | 28.02% |
| P7-LD | LD | 55.11% | 13.06 | 0.45 | 73.03% | 26.15% | 27.54% |
| P7-C1 | C | 57.30% | 13.29 | 45.13 | 77.87% | 31.18% | 33.12% |
| P7-C3 | C | 48.08% | 14.51 | 59.20 | 85.93% | 61.29% | 62.49% |
| P11-N | N | 55.66% | 12.77 | 0.08 | 77.28% | 32.61% | 32.92% |
| P11-LD2 | LD | 58.40% | 13.32 | 0.08 | 77.60% | 33.03% | 33.68% |
| P11-C3 | C | 57.96% | 13.11 | 113.13 | 79.49% | 38.13% | 38.71% |
| P11-C4 | C | 59.19% | 13.28 | 82.29 | 76.72% | 35.18% | 35.57% |
| P13-N1 | N | 58.84% | 15.60 | 7.02 | 77.98% | 38.28% | 40.32% |
| P13-LD | LD | 55.59% | 14.12 | 2.40 | 76.09% | 28.49% | 29.77% |
| P13-C1 | C | 54.90% | 12.02 | 225.42 | 81.49% | 33.47% | 35.85% |
| P13-C5 | C | 55.24% | 13.15 | 246.77 | 80.04% | 27.12% | 29.61% |

**Table S6. List of 67 EBV reference genomes**

| **Genbank No.** | **Strain** | **Geographic origin** | **Disease type^a^** | **EBNA2 type** |
| --- | --- | --- | --- | --- |
| V01555 | B95-8 | USA | IM | 1 |
| NC_007605 | B95-8 (+Raji) | USA | IM | 1 |
| KC440851 | K4123-Mi | USA | sLCL | 1 |
| KC440852 | K4113-Mi | USA | sLCL | 1 |
| MG021317 | EBVaGC5-1 | USA | EBVaGC-tumor | 1 |
| MG021316 | HKNPC6-GC1 | USA | EBVaGC-tumor | 1 |
| MG021315 | EBVaGC8-3 | USA | EBVaGC-tumor | 1 |
| MG021314 | EBVaGC8-2 | USA | EBVaGC-tumor | 1 |
| MG021313 | EBVaGC8-1 | USA | EBVaGC-tumor | 1 |
| AY961628 | GD1 | China | NPC-tumor | 1 |
| HQ020558 | GD2 | China | NPC-tumor | 1 |
| KC617875 | C666-1 | China | NPC-cell line | 1 |
| AB850654 | HN15 | China | NPC-tumor | 1 |
| AB850643 | HN1 | China | NPC-tumor | 1 |
| AB850644 | HN2 | China | NPC-tumor | 1 |
| AB850657 | HN8 | China | NPC-tumor | 1 |
| AB850649 | HN4 | China | NPC-tumor | 1 |
| AB850648 | HN3 | China | NPC-tumor | 1 |
| AB850651 | HN14 | China | NPC-tumor | 1 |
| AB850647 | HN12 | China | NPC-tumor | 1 |
| AB850656 | HN16 | China | NPC-tumor | 1 |
| AB850658 | HN17 | China | NPC-tumor | 1 |
| AB850660 | HN18 | China | NPC-tumor | 1 |
| AB850653 | HN6 | China | NPC-tumor | 1 |
| AB850650 | HN13 | China | NPC-tumor | 1 |
| AB850646 | HN11 | China | NPC-tumor | 1 |
| AB850659 | HN9 | China | NPC-tumor | 1 |
| AB850655 | HN7 | China | NPC-tumor | 1 |
| AB850652 | HN5 | China | NPC-tumor | 1 |
| AB850645 | HN10 | China | NPC-tumor | 1 |
| LC137018 | HNNPC1 | China | NPC-tumor | 1 |
| KF373730 | M81 | Hong Kong | NPC-tumor | 1 |
| JQ009376 | HKNPC1 | Hong Kong | NPC-tumor | 1 |
| KF992564 | HKNPC2 | Hong Kong | NPC-tumor | 1 |
| KF992565 | HKNPC3 | Hong Kong | NPC-tumor | 1 |
| KF992566 | HKNPC4 | Hong Kong | NPC-tumor | 1 |
| KF992567 | HKNPC5 | Hong Kong | NPC-tumor | 1 |
| KF992568 | HKNPC6 | Hong Kong | NPC-tumor | 1 |
| KF992569 | HKNPC7 | Hong Kong | NPC-tumor | 1 |
| KF992570 | HKNPC8 | Hong Kong | NPC-tumor | 1 |
| KF992571 | HKNPC9 | Hong Kong | NPC-tumor | 1 |
| LN824209 | HKN14 | Hong Kong | sLCL | 1 |
| LN824224 | HKN19 | Hong Kong | sLCL | 1 |
| AP015016 | YCCEL1 | Japan | EBVaGC-cell line | 1 |
| AP015015 | SNU-719 | Japan | EBVaGC-cell line | 1 |
| KC207813 | Akata | Japan | BL | 1 |
| LN827800 | Jijoye | Nigeria | BL | 2 |
| LN827548 | P3HR1_c16 | Nigeria | BL | 2 |
| KF717093 | Raji | Nigeria | BL | 1 |
| LN827556 | Cheptages | Kenya | BL | 2 |
| LN827545 | Daudi | Kenya | BL | 1 |
| LN827551 | Makau | Kenya | BL | 1 |
| LN824203 | Mak1 duplicate | Kenya | BL | 1 |
| LN831023 | sLCL-2.22 | Kenya | sLCL | 2 |
| KC207814 | Mutu | Kenya | BL | 1 |
| LN827563 | sLCL-1.18 | Kenya | sLCL | 1 |
| NC_009334 | AG876 | Ghana | BL | 2 |
| LN827557 | BL36 | North Afica | BL | 1 |
| LN824142 | Saliva1 | UK | Healthy saliva | 1 |
| LN824226 | HL01 | UK | HL | 1 |
| LN827564 | HL04 | UK | HL | 1 |
| LN827523 | L591 | Germany | HL-cell line | 1 |
| LN827596 | sLCL-IM1.02 | Australia | sLCL,IM | 1 |
| LN827567 | sLCL-IM1.09 | Australia | sLCL,IM | 1 |
| LN827553 | sLCL-IS1.08 | Australia | sLCL,PTLD | 1 |
| LN827586 | sLCL-IS1.15 | Australia | sLCL,PTLD | 1 |
| LN827589 | sLCL-IS2.01 | Australia | sLCL,PTLD | 2 |
|  |  |  |  |  |
| a: IM, infectious mononucleosis; sLCL, spontaneous lymphoblastoid cell line; EBVaGC, EBV associated gastric carcinoma; NPC, nasopharyngeal carcinoma; BL, Burkitt’s lymphoma; HL, Hodgkin’s lymphoma; PTLD, posttransplant lymphoproliferative disease | | | | |

**Table S7. Thresholds used to filter SNVs on EBV genomes**

| 1 | Total coverage: more than 10 reads supported the mutational site (coverage of forward and reverse strand should have a number more than 3 respectively); |
| --- | --- |
|  |  |
| 2 | Variant allele frequency: more than 3 reads carried the altered base and the VAF was higher than 0.1; |
|  |  |
| 3 | Mapping and base quality: average mapping quality was greater than 20 and average base quality was greater than 30; |
|  |  |
| 4 | Proximal gap: there was no INDEL within an 11-bp window centered on the mutation and fewer than 20 % reads covering the mutational site supported an INDEL at any site along the read; |
|  |  |
| 5 | Mutational position: mutation had more than 10bp distance from the start and end position of the read, meanwhile the absolute deviation between two mutations should be more than 3 (to exclude clustered mutations resulted from misalignments in repetitive regions or other noise); |
|  |  |
|  |  |
| 6 | Strand bias: to reject sequencing errors caused by context bias, a *P*-value should be greater than 0.05 when we performed Fisher’s exact test to compare the fraction of mutant reads in both the forward and reverse strand. |
|  |  |
|  |  |

**Table S8. Validation dataset of RNA-seq and RRBS**

| **Patient** | **Sample** | **Type** | **Sequencing** |
| --- | --- | --- | --- |
| P11 | Normal tissue | Fresh frozen | RNA-seq |
| P11 | EBVaGC | Fresh frozen | RNA-seq |
| P18 | Normal tissue | Fresh frozen | RNA-seq |
| P18 | EBVaGC | Fresh frozen | RNA-seq |
| P30 | Normal tissue | Fresh frozen | RRBS |
| P30 | EBVaGC | Fresh frozen | RRBS |
| P31 | Normal tissue | Fresh frozen | RRBS |
| P31 | EBVaGC | Fresh frozen | RRBS |
| P32 | Normal tissue | Fresh frozen | RRBS |
| P32 | EBVaGC | Fresh frozen | RRBS |

**Table S9. *PIK3CA*, *ARID1A* and *CTNNB1* mutations in AGS, SNU719, HGC27, SNU5 and SNU16 cells**

| **Cell line** | **Gene** | **Chromosome** | **Position** | **Ref** | **Alt** | **Amino acid change** |
| --- | --- | --- | --- | --- | --- | --- |
| AGS | *PIK3CA* | chr3 | 178936092 | A | C | p.E545A |
|  | *PIK3CA* | chr3 | 178928079 | G | A | p.E453K |
|  | *ARID1A* | chr1 | NA | NA | NA | NA |
|  | *CTNNB1* | chr3 | 41266104 | G | A | p.G34E |
| SNU719 | *PIK3CA* | chr3 | 178916924 | C | G | p.P104R |
|  | *ARID1A* | chr1 | NA | NA | NA | NA |
|  | *CTNNB1* | chr3 | 41266104 | G | T | p.G34V |
| HGC27 | *PIK3CA* | chr3 | 178936082 | G | A | p.E542K |
|  | *ARID1A* | chr1 | NA | NA | NA | NA |
|  | *CTNNB1* | chr3 | NA | NA | NA | NA |
| SNU5 | *PIK3CA* | chr3 | NA | NA | NA | NA |
|  | *ARID1A* | chr1 | NA | NA | NA | NA |
|  | *CTNNB1* | chr3 | NA | NA | NA | NA |
| SNU16 | *PIK3CA* | chr3 | NA | NA | NA | NA |
|  | *ARID1A* | chr1 | NA | NA | NA | NA |
|  | *CTNNB1* | chr3 | NA | NA | NA | NA |

The mutation lists of all cell lines used in this study are obtained from the Cancer Cell Line Encyclopedia (CCLE) (<https://portals.broadinstitute.org/ccle>)

**References**

1. Miura F, Enomoto Y, Dairiki R, Ito T: **Amplification-free whole-genome bisulfite sequencing by post-bisulfite adaptor tagging**. *Nucleic acids research* 2012, **40**(17):e136.

2. Li H, Durbin R: **Fast and accurate short read alignment with Burrows-Wheeler transform**. *Bioinformatics* 2009, **25**(14):1754-1760.

3. Li H, Handsaker B, Wysoker A, Fennell T, Ruan J, Homer N, Marth G, Abecasis G, Durbin R, Genome Project Data Processing S: **The Sequence Alignment/Map format and SAMtools**. *Bioinformatics* 2009, **25**(16):2078-2079.

4. McKenna A, Hanna M, Banks E, Sivachenko A, Cibulskis K, Kernytsky A, Garimella K, Altshuler D, Gabriel S, Daly M *et al*: **The Genome Analysis Toolkit: a MapReduce framework for analyzing next-generation DNA sequencing data**. *Genome research* 2010, **20**(9):1297-1303.

5. Cibulskis K, Lawrence MS, Carter SL, Sivachenko A, Jaffe D, Sougnez C, Gabriel S, Meyerson M, Lander ES, Getz G: **Sensitive detection of somatic point mutations in impure and heterogeneous cancer samples**. *Nature biotechnology* 2013, **31**(3):213-219.

6. Saunders CT, Wong WS, Swamy S, Becq J, Murray LJ, Cheetham RK: **Strelka: accurate somatic small-variant calling from sequenced tumor-normal sample pairs**. *Bioinformatics* 2012, **28**(14):1811-1817.

7. Stachler MD, Taylor-Weiner A, Peng S, McKenna A, Agoston AT, Odze RD, Davison JM, Nason KS, Loda M, Leshchiner I *et al*: **Paired exome analysis of Barrett's esophagus and adenocarcinoma**. *Nature genetics* 2015, **47**(9):1047-1055.

8. Chen XX, Zhong Q, Liu Y, Yan SM, Chen ZH, Jin SZ, Xia TL, Li RY, Zhou AJ, Su Z *et al*: **Genomic comparison of esophageal squamous cell carcinoma and its precursor lesions by multi-region whole-exome sequencing**. *Nat Commun* 2017, **8**(1):524.

9. Rosenthal R, McGranahan N, Herrero J, Taylor BS, Swanton C: **DeconstructSigs: delineating mutational processes in single tumors distinguishes DNA repair deficiencies and patterns of carcinoma evolution**. *Genome biology* 2016, **17**:31.

10. Carter SL, Cibulskis K, Helman E, McKenna A, Shen H, Zack T, Laird PW, Onofrio RC, Winckler W, Weir BA *et al*: **Absolute quantification of somatic DNA alterations in human cancer**. *Nature biotechnology* 2012, **30**(5):413-421.

11. Gibson WJ, Hoivik EA, Halle MK, Taylor-Weiner A, Cherniack AD, Berg A, Holst F, Zack TI, Werner HM, Staby KM *et al*: **The genomic landscape and evolution of endometrial carcinoma progression and abdominopelvic metastasis**. *Nature genetics* 2016, **48**(8):848-855.

12. Roth A, Khattra J, Yap D, Wan A, Laks E, Biele J, Ha G, Aparicio S, Bouchard-Cote A, Shah SP: **PyClone: statistical inference of clonal population structure in cancer**. *Nat Methods* 2014, **11**(4):396-398.

13. Yates LR, Knappskog S, Wedge D, Farmery JHR, Gonzalez S, Martincorena I, Alexandrov LB, Van Loo P, Haugland HK, Lilleng PK *et al*: **Genomic Evolution of Breast Cancer Metastasis and Relapse**. *Cancer Cell* 2017, **32**(2):169-184 e167.

14. Palser AL, Grayson NE, White RE, Corton C, Correia S, Ba Abdullah MM, Watson SJ, Cotten M, Arrand JR, Murray PG *et al*: **Genome diversity of Epstein-Barr virus from multiple tumor types and normal infection**. *J Virol* 2015, **89**(10):5222-5237.

15. Katoh K, Misawa K, Kuma K, Miyata T: **MAFFT: a novel method for rapid multiple sequence alignment based on fast Fourier transform**. *Nucleic acids research* 2002, **30**(14):3059-3066.

16. Stamatakis A: **RAxML version 8: a tool for phylogenetic analysis and post-analysis of large phylogenies**. *Bioinformatics* 2014, **30**(9):1312-1313.

17. Murrell B, Weaver S, Smith MD, Wertheim JO, Murrell S, Aylward A, Eren K, Pollner T, Martin DP, Smith DM *et al*: **Gene-wide identification of episodic selection**. *Molecular biology and evolution* 2015, **32**(5):1365-1371.

18. Delport W, Poon AF, Frost SD, Kosakovsky Pond SL: **Datamonkey 2010: a suite of phylogenetic analysis tools for evolutionary biology**. *Bioinformatics* 2010, **26**(19):2455-2457.

19. Krueger F, Andrews SR: **Bismark: a flexible aligner and methylation caller for Bisulfite-Seq applications**. *Bioinformatics* 2011, **27**(11):1571-1572.

20. Hansen KD, Langmead B, Irizarry RA: **BSmooth: from whole genome bisulfite sequencing reads to differentially methylated regions**. *Genome biology* 2012, **13**(10):R83.

21. Akalin A, Franke V, Vlahovicek K, Mason CE, Schubeler D: **Genomation: a toolkit to summarize, annotate and visualize genomic intervals**. *Bioinformatics* 2015, **31**(7):1127-1129.

22. Heerma van Voss MR, Brilliant JD, Vesuna F, Bol GM, van der Wall E, van Diest PJ, Raman V: **Combination treatment using DDX3 and PARP inhibitors induces synthetic lethality in BRCA1-proficient breast cancer**. *Med Oncol* 2017, **34**(3):33.

23. Saitoh Y, Setoguchi T, Nagata M, Tsuru A, Nakamura S, Nagano S, Ishidou Y, Nagao-Kitamoto H, Yokouchi M, Maeda S *et al*: **Combination of Hedgehog inhibitors and standard anticancer agents synergistically prevent osteosarcoma growth**. *Int J Oncol* 2016, **48**(1):235-242.

24. Zhou P, Qin J, Li Y, Li G, Wang Y, Zhang N, Chen P, Li C: **Combination therapy of PKCzeta and COX-2 inhibitors synergistically suppress melanoma metastasis**. *J Exp Clin Cancer Res* 2017, **36**(1):115.
