# Supplementary material for: The genomic architecture of EBV and infected gastric tissue from precursor lesions to carcinoma
Source: Genome Med. 2021 Sep 7;13:146. doi: 10.1186/s13073-021-00963-2 (PMC8422682; doi:10.1186/s13073-021-00963-2)
Supplement: Supplementary file 2 — Additional file 2. Full images of the western blotting results (Fig. S14-S15). [file 13073_2021_963_MOESM2_ESM.docx]

**Additional file 2**

**The genomic architecture of EBV and infected gastric tissue from precursor lesions to carcinoma**

Zhang-Hua Chen^1#^, Shu-Mei Yan^2,3#^, Xi-Xi Chen^1#^, Qi Zhang^2,4,8#^, Shang-Xin Liu^2#^, Yang Liu^1^, Yi-Ling Luo^2^, Chao Zhang^6,7^, Miao Xu^2^, Yi-Fan Zhao^1^, Li-Yun Huang^2,3^, Bin-Liu Liu^2^, Tian-Liang Xia^2^, Da-Zhi Xu^5^, Yao Liang^5^, Yong-Ming Chen^5^, Wei Wang^5^, Shu-Qiang Yuan^5^, Hui-Zhong Zhang^2,3^, Jing-Ping Yun^2,3^, Wei-Wei Zhai^9,10^, Mu-Sheng Zeng^2^, Fan Bai^1,11*^, Qian Zhong^2*^

^1^Biomedical Pioneering Innovation Center (BIOPIC), School of Life Sciences, Peking University, Beijing, China.

^2^State Key Laboratory of Oncology in South China, Collaborative Innovation Center for Cancer Medicine, Sun Yat-sen University Cancer Center, Guangzhou, China.

^3^Department of Pathology, Sun Yat-sen University Cancer Center, Guangzhou, China.

^4^Department of Oncology, Second Affiliated Hospital, Guangzhou Medical University, Guangzhou, China.

^5^Department of Gastric Surgery, Sun Yat-sen University Cancer Center, Guangzhou, China.

^6^Institute for Computational Biomedicine, Weill Cornell Medicine, New York, NY 10021, USA

^7^Division of Hematology/Oncology, Department of Medicine, Weill Cornell Medicine, New York, NY 10021, USA

^8^Department of Ultrasound, The Fifth Affiliated Hospital of Sun Yat-sen University, Zhuhai, China.

^9^Key Laboratory of Zoological Systematics and Evolution, Institute of Zoology, Chinese Academy of Sciences, Beijing, China.

^10^Center for Excellence in Animal Evolution and Genetics, Chinese Academy of Sciences, Kunming, China.

^11^Beijing Advanced Innovation Center for Genomics (ICG), Peking University, Beijing, China.

**
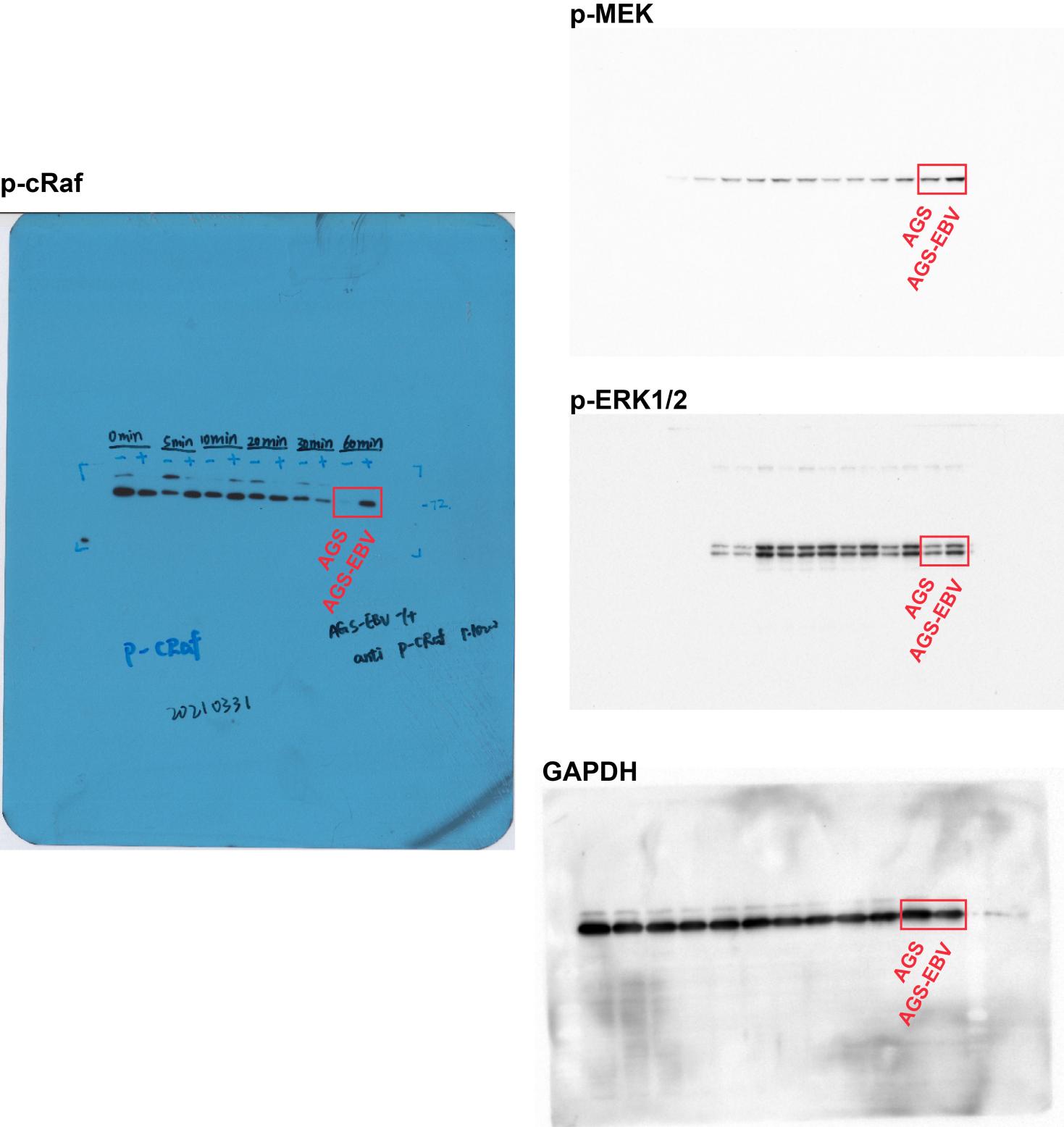
**

**Fig. S14**

**Full image of western blotting result of RAS pathway activity in AGS cells before and after EBV infection.**

**
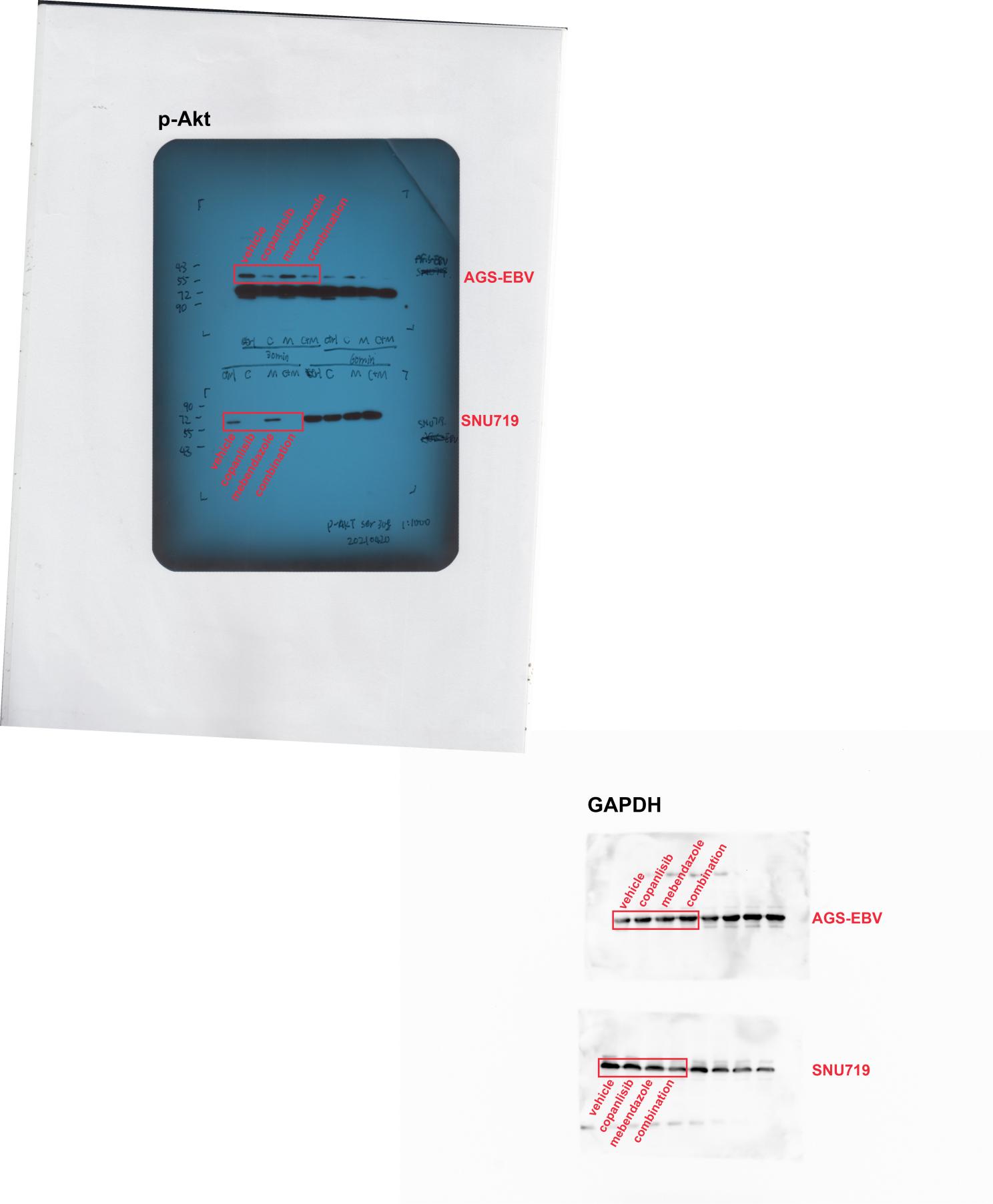
**

**Fig. S15**

**Full image of western blotting result of p-Akt levels in SNU719 and AGS-EBV cells under different treatments.**
